# Coagulative safety of epidural catheters after major upper gastrointestinal surgery: advanced and routine coagulation analysis in 38 patients

**DOI:** 10.1186/s13741-016-0053-0

**Published:** 2016-10-18

**Authors:** Owain Thomas, Hampus Rein, Karin Strandberg, Ulf Schött

**Affiliations:** 1 Faculty of Medicine, University of Lund, 22100 Lund, Sweden; 2 Department of Paediatric Anaesthesia and Intensive Care, SUS Lund University Hospital, 22185 Lund, Sweden; 3 Coagulation Laboratory, Department of Clinical Chemistry, Division of Laboratory Medicine, Skåne University Hospital, 21428 Malmö, Sweden; 4 Department of Anaesthesia and Intensive Care, SUS Lund University Hospital, 22185 Lund, Sweden; 5 Visby Hospital, 62155 Gotland, Sweden

**Keywords:** Blood coagulation factors, International normalized ratio, Partial thromboplastin time, Thromboelastometry, Platelet aggregation, Epidural analgesia, Postoperative complications, Hematoma, Safety

## Abstract

**Background:**

The risk of spinal haematoma in patients receiving epidural catheters is estimated using routine coagulation tests, but guidelines are inconsistent in their recommendations on what to do when results indicate slight hypocoagulation. Postoperative patients are prone to thrombosis, and thromboelastometry has previously shown hypercoagulation in this setting. We aimed to better understand perioperative haemostasis by comparing results from routine and advanced tests, hypothesizing that patients undergoing major upper gastrointestinal surgery would be deficient in vitamin K-dependent coagulation factors because of malnutrition, or hypocoagulative because of accumulation of low molecular weight heparin (LMWH).

**Methods:**

Thirty-eight patients receiving epidural analgesia for major upper gastrointestinal surgery were included. We took blood at the time of preoperative epidural catheterization and at catheter withdrawal. Prothrombin time-international normalized ratio (PT-INR), activated partial thromboplastin time (aPTT) and platelet count (Plc) were analysed, and also albumin, proteins induced by vitamin K absence (PIVKA-II), rotational thromboelastometry (ROTEM®), multiple electrode aggregometry (Multiplate®) and activities of factors II, VII, IX, X, XI, XII and XIII.

**Results:**

Postoperative coagulation was characterized by thrombocytosis and hyperfibrinogenaemia. Mean PT-INR increased significantly from 1.0 ± 0.1 to 1.2 ± 0.2 and mean aPTT increased significantly from 27 ± 3 to 30 ± 4 s. Activity of vitamin K-dependent factors did not decrease significantly: FIX and FX activity increased. FXII and FXIII decreased significantly. Mean Plc increased from 213 ± 153 × 10^6^/L while all mean ROTEM-MCFs (maximal clot firmnesses) especially FIBTEM-MCF increased significantly to above the reference interval. All mean ROTEM® clotting times were within their reference intervals both before and after surgery. ROTEM® (HEPTEM minus INTEM) results were spread around 0. There were significant correlations between routine tests and the expected coagulation factors, but not any of the viscoelastic parameters or PIVKA-II. Multiplate® area under curve and EXTEM-MCF correlated significantly to Plc as did EXTEM-MCF to fibrinogen, FIX, FX and FXIII; and FIBTEM-MCF to Plc, FII, FXI and FXIII.

**Conclusions:**

The increase in PT-INR may be caused by decreased postoperative FVII while the elevated aPTT may be caused by low FXII. The mild postoperative hypocoagulation indicated by routine tests is not consistent with thromboelastometry. The relevance of ROTEM® and Multiplate® in the context of moderately increased routine tests remains unclear.

Trial registration number is not applicable since this is not a clinical trial.

**Electronic supplementary material:**

The online version of this article (doi:10.1186/s13741-016-0053-0) contains supplementary material, which is available to authorized users.

## Background

Epidural anaesthesia is used extensively in modern practice and likely decreases morbidity and mortality after major surgery involving thoracotomy (van Lier et al. 2011). Neurological damage due to haemorrhage in the spinal canal is rare but well-described (De Tommaso et al. 2002; Horlocker et al. 2010a) and is most likely to occur at the times of insertion and removal of epidural catheters, when the incidences are estimated to be 1:4000–1:30,000 and 1:150,000–1:190,000 (Moen et al. 2004; Miyazaki et al. 2005; Pace et al. 2014). Certain types of patients are at greater risk of spinal haematoma than others: elderly women undergoing orthopaedic surgery are at particularly high risk (Moen et al. 2004). Coagulative deficits are often noted after major surgery (Anveden et al. 2010) and increase the risk of spinal haematoma, which may be caused by loss of coagulation factors (F) and platelets due to haemorrhage, systemic inflammation, disseminated intravascular coagulation (DIC), preoperative malnutrition, accumulation of thrombosis prophylaxis or deficiency of vitamin K due to prolonged antibiotic treatment (van Lier et al. 2011; Gogarten et al. 2010). Routine tests of coagulation such as prothrombin time-international normalized ratio (PT-INR), activated partial thromboplastin time (aPTT) and platelet count (Plc) are unreliable predictors of perioperative bleeding, and their usefulness in assessing the risk of spinal haemorrhage in the context of epidural catheterization is limited (Ganter and Hofer 2008; Breivik et al. 2010; Chee et al. 2008). Although many authors stress the importance of coagulation monitoring, both before inserting and epidural catheter and when the catheter needs to be removed, current international guidelines are vague concerning what constitutes a coagulation deficit and how these should be treated: both the European and American guidelines are primarily concerned with drug-induced hypocoagulation rather than spontaneously occurring deficits of coagulation (Davignon et al. 2008): Additional file 1 summarizes these (Gogarten et al. 2010; Breivik et al. 2010). A strategy employed at our institution when an epidural catheter is due for removal but aPTT or PT-INR are elevated, is postponement of treatment with low molecular weight heparins (LMWHs), administration of intravenous vitamin K and renewed testing the following day. Some recommend whole-blood tests such as viscoelasticity and platelet aggregometry (for example rotational thromboelastometry (ROTEM®) and Multiplate®), but how these complement routine tests and how to interpret inconsistencies between routine and whole-blood tests is unclear (Breivik et al. 2010; Thomas et al. 2013; Tanaka and Dietrich 2011).

The aim of our study was to describe and compare patterns in routine coagulation tests, thromboelastometry and platelet aggregation, coagulation factor activity and levels of PIVKA (proteins induced by vitamin K absence) before and after major surgery, to better understand how these tests reflect perioperative coagulation in the specific context of epidural catheterization. We hypothesized that preoperative malnutrition in patients with upper gastrointestinal pathology would lead to a depletion of vitamin K-dependent coagulation factors. Thereby, we hoped to be able to evaluate the appropriateness of the above described clinical strategy used when an epidural catheter is to be withdrawn in a patient with abnormal routine coagulation test results. We also hypothesized that thromboelastometry with a heparinase reagent (HEPTEM®) would indicate an accumulation of LMWH due to postoperative renal failure combined with thrombosis prophylaxis.

## Methods

### Participants

Thirty-eight patients were included in the study over a total period of 8 months in 2013 and 2014. Included in the study were patients scheduled for general anaesthesia and epidural analgesia for major upper gastrointestinal surgery but not liver resection. Patients completed a preoperative questionnaire covering general health, nutrition and medication, bleeding history and risk of haemorrhage. Those with deranged preoperative coagulation parameters, i.e. low blood Plc, increased PT-INR or prolonged aPTT, and those receiving therapeutic doses of LMWH were not included.

### Blood sampling

See Table 1 and Additional file 2 for a summary of the tests analysed and reagents used.

**Table 1:** Assays taken and reference intervals

Parameter	Reference interval
ROTEM EXTEM-MCF (maximal clot firmness)	50–72 mm
ROTEM EXTEM-CT (clotting time)	38–79 mm
ROTEM INTEM-MCF	50–72 mm
ROTEM INTEM-CT	100–240 s
ROTEM FIBTEM-MCF	9–25 mm
ROTEM HEPTEM-MCF	50–72 mm
ROTEM HEPTEM-CT	100–240 s
Multiplate® AUC (area under curve): ADPtest	57–113 U
Multiplate® AUC: COLtest	72–125 U
Multiplate® AUC: TRAPtest	84–128 U
Multiplate® AUC: ASPItest	71–115 U
P-FII	0.70–1.50 kIE/L
P-FVII	0.60–1.60 kIE/L
P-FX	0.70–1.52 kIE/L
P-FIX	0.70–1.30 kIE/L
P-FXI	0.60–1.30 kIE/L
P-FXII	1.07–1.50 kIE/L
P-FXIII	0.70–1.40 kIE/L
PT-INR	0.9–1.1
aPTT	26–33 s
Platelet count (Plc)	Women 165–387 Men 145–348 × 10^9^/L
P-Fibrinogen	2.0–4.0 mg/mL
D-dimer	<0.25 mg/L
Gamma-glutamyltransferase (GT)	0.2–1.9 μkat/L
C-reactive protein (CRP)	<3.0 mg/L
Bilirubin	5–25 μmol/L
Alkaline phosphatase (ALP)	0.6–1.8μkat/L
Creatinine	45–90 μmol/L
Haemoglobin (Hb)	Women 117–153 g/L Men 134–170
PIVKA-II (protein induced by vitamin K absence)	<2.0 mg/L

See Additional file 2 for a technical specification of the apparatuses and reagents used

*Preoperative* blood samples were taken immediately after placement of an arterial catheter in the operating room. Postoperative blood samples were taken on the surgical ward, from patients’ central venous catheters within 4 h of withdrawal of the patient’s epidural catheter when it was withdrawn as part of the patient’s routine care. Patients’ central venous catheters were not heparinized.

Routine analyses were run at our hospital’s Department of Clinical Chemistry, which is accredited by SWEDAC (Borås, Sweden). The following sampling tubes were filled on each occasion: one 4.5-mL citrate tube (0.129 M citrate, BD Vacutainer® systems, Plymouth, UK) for routine PT-INR, aPTT, fibrinogen and D-dimer; one 3-mL EDTA tube (K_2_EDTA, BD Vacutainer® systems, Plymouth, UK) for routine platelet count; one 3-mL lithium-heparin tube (LH PST, BD Vacutainer® systems, Plymouth, UK) for routine alkaline phosphatase (ALP), C-reactive protein (CRP), gamma-glutamyltransferase (GT) and creatinine; one 3-mL hirudinated blood tube for whole-blood multiple electrode platelet aggregometry (Multiplate®) at 37 °C according to the manufacturer’s instructions, at our patient-near laboratory approximately 30 min after sampling; one 1-mL heparinized syringe for ‘blood gas analysis’ including haemoglobin (Hb) (PICO 50, Radiometer medical ApS, Brønshøj, Denmark); two additional citrate tubes (3.2 % citrate, BD Vacutainer® systems, Plymouth, UK) for thromboelastometry using four reagents at 37 °C within 2 h of sampling, and for centrifugation at 2000 revolutions/min (rpm) for 20 min (Hettich Zentrifugen, 78532 Tuttlingen, Germany). Batches of 500 μL of the resultant plasma were pipetted into six micro tubes (Finnpipette™, Thermo Electron Corporation 1.5 mL, Sarstedt, Nümbrecht, Germany) and frozen to −80 °C awaiting analysis of coagulation factors and PIVKA-II in a batch at the coagulation laboratory. The following factors’ activities were determined with a clot-based one-stage method: FII, FVII, FIX, FX, FXI, FXII and FXIII.

### Markers of malnutrition

The plasma concentration of PIVKA-II was determined. PIVKA-II is hypocarboxylated prothrombin produced when there is a deficiency of vitamin K (Dituri et al. 2012). Each test was run in duplicate and the mean of the results used. Patients’ preoperative serum albumin was recorded from their notes.

### Statistical analysis

Primary data was compiled in a Microsoft® Excel spreadsheet then exported to the R statistics package (version 3.0.3, www.r-project.org) for analysis. See Additional file 3. Mean vales are presented as mean ± standard deviation. Wilcoxon’s signed ranked test for paired samples was used to determine the significance (*p* < 0.05) of differences between pre- and postoperative variables. Pearson’s (parametric) product–moment and Spearman’s (non-parametric) rank correlation test were used to calculate the significance of correlations between parameters. Two tests of correlation were used because non-parametric tests are more suitable for this rather small data set, but since PT-INR results are recorded to the nearest 0.1 units, there were many tied results making non-parametric tests using ranks unreliable. Correlations are presented as scatter graphs while original data are presented as boxplots and tables.

## Results

Epidemiological data is shown in Table 2. All the patients received a thoracic epidural catheter before induction of anaesthesia and were administered daily thrombosis prophylaxis in the form of 40 mg of subcutaneous enoxaparin (Klexane®, Sanofi) at 8.00 p.m. One of the patients was postoperatively given enoxaparin in therapeutic doses of 110 mg twice daily due to postoperative pulmonary embolism. None of the patients received platelet inhibitors during the study period; one patient had been anticoagulated with warfarin until 5 days before surgery and one until 2 months before surgery. Three received ‘bridging’: during the week before surgery LMWH was given in prophylactic doses instead of normal ASA or warfarin.

**Table 2:** Epidemiological data and indications for surgery

Epidemiological data	
Sex	27 males;11 females
Mean age	70.2 ± 6.9 years (range 57–82 years)
Mean duration of operation	10.4 h
Median perioperative blood loss	400 mL (range 50–4650 mL)
Mean perioperative fluid loss through surgical drains	430 ± 355 mL
Mean time from epidural catheterization to withdrawal	6.2 ± 3.2 days (range 2–15)
Included and preoperative tests taken, but postoperative tests not taken due to transfer to other hospital or researcher being absent.	5 patients
Indication for surgery	Number of patients
Gastric cancer	17
Oesophageal cancer	4
Gastrooesophageal cancer	2
Gallbladder cancer or cholangiocarcinoma	3
Pancreatic cancer	9
Tumour in ampulla of Vater	1
Cancer simultaneously involving upper gastrointestinal tract and colon	2

Table 3 shows routine laboratory results. Mean preoperative serum albumin was at the lower end of the reference interval and serum creatinine decreased slightly but significantly over the perioperative course.

**Table 3:** Routine laboratory results

	Preoperative results	Postoperative results	Reference range
PT-INR	1.0 ± 0.1	1.2 ± 0.2*	0.9–1.1
aPTT (s)	27 ± 3	30 ± 4*	26–33
Plc (× 10^9^/L)	213 ± 153	283 ± 153*	145–387
Fibrinogen (g/L)	3.2 ± 0.9	*5.8 ± 1.1*	2.0–4.0
CRP (mg/L)	*6.8 ± 9.4*	*92 ± 60*	<3.0
D-dimer (mg/L)	*0.25 ± 0.22*	*1.73 ± 1.05*	<0.25
Serum albumin (g/L)	36.6 ± 3.7	–	36–45
Serum creatinine (μmol/L)	73.4 ± 19	65.2 ± 18.3*	45–105
Serum bilirubin (μmol/L)	14 ± 22	10 ± 16*	5–25
ALP (μkat/L)	1.64 ± 1.1	2.30 ± 1.3*	0.6–1.8
Bilirubin (μmol/L)	14 ± 22	11 ± 16*	5–25
GT (μkat/L)	1.3 ± 2.0	2.1 ± 1.9*	0.2–1.9
Blood haemoglobin (g/L)	119 ± 16 g/L	*113 ± 12 g/L*	Men 134–170; women 117–153

Italics indicate results outside the reference interval. Results are presented as mean ± standard deviation

*ALP* alkaline phosphatase, *GT* gamma-glutamyltransferase, *PT-INR* prothrombin-international normalized ratio, *aPTT* activated partial thromboplastin time, *Plc* platelet count, *CRP* C-reactive protein

*Significant difference between pre- and postoperative values (*P* < 0.05)

### Viscoelastic and routine tests of coagulation

**Fig. 1:**
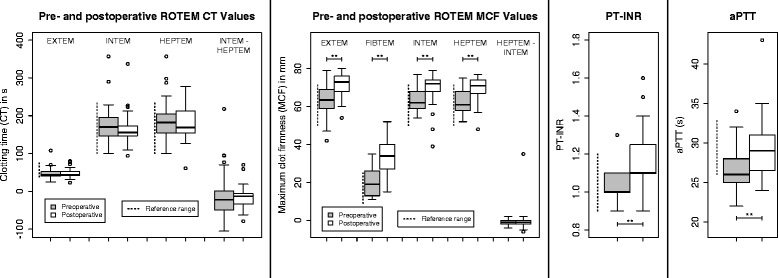
Pre- to postoperative changes in thromboelastometry (ROTEM®) and routine tests of coagulation. *PT-INR* prothrombin time-international normalized ratio, *aPTT* activated partial thromboplastin time. *Dashed lines* indicate reference intervals, *brackets* indicate significant differences in pre- and postoperative results *P < 0.05; **P < 0.01

See Fig. 1 and Table 3. All ROTEM® parameters’ mean maximal clot firmnesses (MCFs) were within the reference interval preoperatively and significantly greater than the reference interval postoperatively. FIBTEM-MCF indicated hypercoagulation more than the other measures. All the mean ROTEM® clotting times (CTs) were within their reference intervals, and there was no significant difference between pre- and postoperative results

The distributions of ROTEM® HEPTEM-INTEM CT and MCF were somewhat surprisingly centred around 0: results activated with the INTEM reagent and a heparinase often showed hypocoagulability compared to results from tests activated with the INTEM reagent alone.

Mean PT-INR increased significantly: all of the patients’ preoperative PT-INR values were within the reference interval, whereas eight exceeded it postoperatively: the maximum PT-INR was 1.6 in a patient whose epidural was removed on the second postoperative day after stomach resection. His aPTT was 26 s and PIVKA-II 12.5 mg/L. Coagulation factor activities for this patient were within the reference ranges other than FII (0.55 kIE/L), FXII (0.37 kIE/L) and FXIII (0.58 kIE/L).

**Fig. 2:**
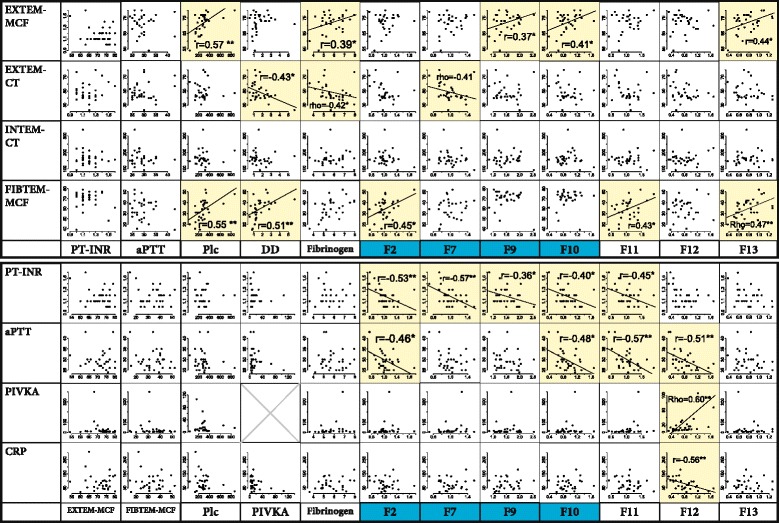
Correlations between routine, viscoelastic and advanced tests of coagulation and CRP. *Blue*: vitamin K-dependent factor. *Yellow*: significant correlation. *P < 0.05. **P < 0.01. *Dashed line*: only one of Spearman and Pearson’s tests is significant, the other is slightly more than *P* = 0.05

Mean aPTT increased significantly over the perioperative course. Still, only five of the patients’ postoperative aPTT values exceeded the upper reference limit. There was no correlation between age and postoperative aPTT nor were there significant correlations between PT-INR or aPTT and any of the viscoelastic parameters (see Fig. 2).

### Platelet results

**Fig. 3:**
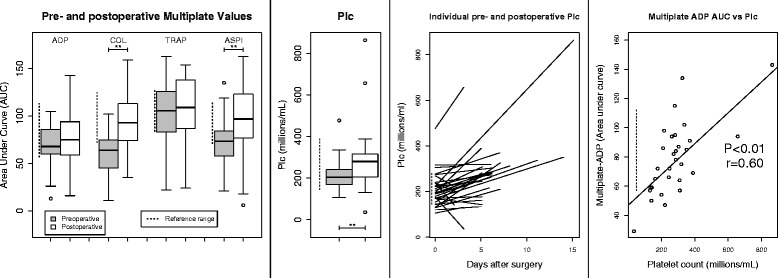
*Boxplots* show pre- to postoperative changes in platelet aggregometry (Multiplate®) and platelet count (Plc). *Dashed lines* indicate reference intervals, *brackets* indicate significant differences between pre- and postoperative results *P < 0.05; **P < 0.01

Mean Plc was significantly higher postoperatively: see Fig. 3 and Table 3. All the patients whose Plc was significantly lower on withdrawal of the epidural catheter than before the operation had their epidural withdrawn on the third postoperative day or earlier. One patient’s epidural catheter was accidentally withdrawn when Plc was 35 × 10^9^/L: see Additional file 4. All mean pre- and postoperative Multiplate® ADP-AUC (area under curve) results were within the reference range, correlated significantly to Plc with a correlation coefficient of 0.6 and increased significantly pre- to postoperatively.

There was a significant correlation between postoperative platelet count and ROTEM-MCF using both the EXTEM and FIBTEM reagents, but not between Plc and any ROTEM® CT results: see Fig. 2.

### Fibrinogen, CRP and PIVKA-II

**Fig. 4:**
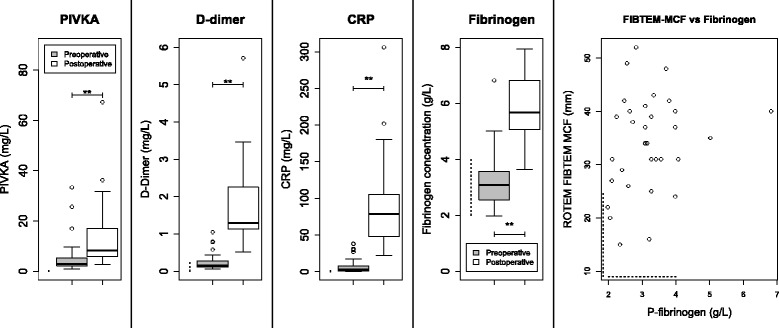
*Boxplots* show pre- to postoperative changes in PIVKA (protein induced by vitamin K absence), D-dimer, CRP and fibrinogen levels. Note the lack of correlation between postoperative fibrinogen and ROTEM® FIBTEM-MCF. Note that two PIVKA outliers (106 and 398 mg/L) lie above the axes’ limits. *Dashed lines* indicate reference intervals, *brackets* indicate significant differences between pre- and postoperative results *P < 0.05; **P < 0.01

See Figs. 1 and 4. Mean fibrinogen levels increased significantly, and although ROTEM® FIBTEM-MCF also increased, it did not correlate to postoperative fibrinogen concentration. There were weak significant correlations between postoperative fibrinogen and both EXTEM-MCF and EXTEM-CT but not FIBTEM-MCF: see Fig. 2.

Mean CRP increased significantly over the perioperative course, and while both mean pre- and postoperative PIVKA-II levels were greater than the manufacturer’s reference range, postoperative values were significantly greater than preoperative ones. There was no significant correlation between PIVKA-II and PT-INR or CRP.

Of the 15 patients whose postoperative PT-INR was equal to or greater than 1.2, the median postoperative PIVKA-II was 8.2 mg/L. The patient with the highest preoperative PIVKA-II of 33 had impaired bile drainage due to a stricture in ductus choledocus: bilirubin and ALP were both elevated. PIVKA-II increased to 106 mg/L in this patient, but all other test results were within the normal range. The patient who had been treated with warfarin until 5 days before operation also had biliary stasis and had an elevated preoperative PIVKA-II of 25 mg/L, but this had almost normalised to 6.8 mg/L when his epidural was withdrawn on the sixth postoperative day. The patient who had received warfarin until 2 months before the operation had a slightly elevated preoperative PIVKA-II of 2.8, which rose to 5.4 mg/L postoperatively. One patient, operated for stomach cancer, saw a dramatic rise in PIVKA-II levels from 4.7 to 398 mg/L in the absence of other remarkable test results.

### Advanced coagulation test results

**Fig. 5:**
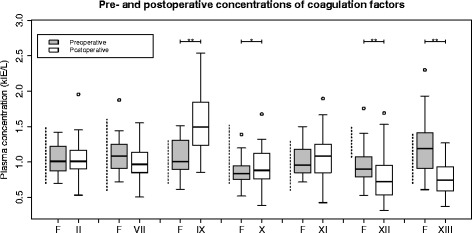
*Boxplots* show pre- to postoperative changes in coagulation factor activities. Factors II, VII, IX and X are vitamin K-dependent. *Dashed lines* indicate reference intervals, *brackets* indicate significant differences between pre- and postoperative results *P < 0.05; **P < 0.01

See Figs. 4 and 5. Mean FIX and FX concentrations increased significantly over the perioperative period, especially FIX whose mean postoperative concentration increased from to above its upper reference limit. Levels of FXII and FXIII decreased significantly: preoperative mean FXII was already below the reference interval, and decreased significantly to 0.80 ± 0.33 kIE/L postoperatively (reference range 1.07–1.50 kIE/L). Although factor XIII levels decreased significantly, both the pre- and postoperative mean levels were within the reference range.

Mean FII, FVII and FXI concentrations neither increased nor decreased significantly pre- to postoperatively, and their mean values were all within the reference ranges. There were no highly significant strong correlations between postoperative coagulation factor levels and other test results. The following factors demonstrate weak significant correlations to ROTEM® results: FII to FIBTEM-MCF; FVII to EXTEM-CT, FIX to EXTEM-MCF, FX to EXTEM-MCF, FXIII to EXTEM-MCF: see Fig. 2.

## Discussion

Our results give some insight into the normal changes in coagulation after major surgery, which is anything but a normal condition for the body: Unfortunately, our results do not allow us to come to a ‘blanket conclusion’ that patients are always hypercoagulable such that routine tests can be ignored. The changes in coagulation factors shown in this study do nevertheless provide some tentative explanations for the elevated routine tests of coagulation, and it would be reasonable to conclude that the risk of hypocoagulation due to thrombocytopenia or low levels of coagulation factors is greatest during the first days after major operations, after which a systemic inflammation and hyperfibrinogenaemia compensates for potential deficits due to deficiencies in coagulation factors and patients generally become hypercoagulable. The incongruence between routine coagulation tests and the other tests investigated in this study suggests that slightly elevated PT-INR and aPTT are unlikely to represent serious coagulopathy.

PT-INR and aPTT’s tendencies towards hypocoagulation are likely due to low levels of factors VII and XII, respectively, and despite low postoperative levels of factor XIII, whole-blood tests and Plc indicate normo- or moderate hypercoagulation. This is in agreement with previous work after hepatectomy by Louis et al. who found the same discrepancy between PT-INR and thrombelastography (TEG) and Elterman et al. who found that after a transient period of hypocoagulability, PT-INR stabilized around 1.3 from the fourth postoperative day (Louis et al. 2014; Elterman and Xiong 2015).

Interpretation of coagulation factor activity is difficult since their activities are interrelated: interactions rather than absolute levels of coagulation factors ultimately result in thrombin’s conversion of fibrinogen to fibrin and most coagulation factors are not themselves used up by coagulation. Changes in the concentration of individual factors do not therefore predictably increase or decrease thrombin and fibrin generation. For example, 50 % changes in the concentrations of most vitamin K-dependent factors were shown by Butenas et al. to have little effect on thrombin generation, and an increase in factor XI, which is required in the early stages of the intrinsic coagulation pathway, actually decreased thrombin generation if all other factor levels were unchanged (Butenas et al. 1999). Similarly, isolated variations in the concentrations of vitamin K-dependent factors II, VII and Z in warfarin-treated patients may have greater effects on both PT and thrombin generation than other factors.

### Acquired FXII deficiency explains elevated aPTT

The weak significant correlations between aPTT results and levels of FII, FX, FXI and FXII are theoretically consistent with these being factors involved in the intrinsic pathway. Factor XII is required for activation of coagulation, such that the low pre- and postoperative results that we observed (see Fig. 5) may be responsible for the prolonged aPTT. Synthesis of FXII is *decreased* by systemic inflammation, explaining the negative correlation between postoperative FXII and CRP (Citarella et al. 1997). Even patients with a complete deficiency of FXII do not have seriously dysfunctional coagulation since FXI can be activated by FIIa: the substantial increase in FIX seen in our patients ought to provide additional compensation. We therefore question the appropriateness of current guidelines’ insistence that a ‘normal’ aPTT is required for the safe withdrawal of an epidural catheter in the absence of other risk factors for haemorrhage (Gogarten et al. 2010; Breivik et al. 2010; Horlocker et al. 2010b). On the other hand, aPTT may be seen as a cheap and specific, yet insensitive, screening for excessive accumulation of LMWH, even though we actually saw no evidence of this in our patients—see below (Thomas et al. 2015).

### Acquired FVII deficiency may explain prolonged PT and ROTEM® EXTEM-CT

The significant yet weak negative correlations shown between FVII and both PT-INR and EXTEM-CT (see Fig. 2) imply that decreases in FVII cause elevations of these parameters: activation of FVII by exposure to tissue factor is responsible for initiation of the extrinsic pathway (Hoffman and Monroe 2001). Postoperative FVII levels in the group as a whole were, however, almost all within the reference interval and not quite significantly lower than preoperative results (*P* = 0.07).

Previous work has shown that PT-INR is not elevated until FII activity is less than 50 % of its normal level nor is it a good predictor of clinical haemorrhage (Segal and Dzik 2005); despite ROTEM® being frequently and enthusiastically described as a global measure of coagulation, Ninivaggi et al. found that it is unreliable in the detection of therapeutic vitamin K antagonist activity: in the context of this article, it would have low sensitivity and high specificity for patients with coagulopathy (Ninivaggi et al. 2014). Given that congenital deficiencies of FVII cause clinical bleeding disorders and both PT-INR and EXTEM-CT correlate to FVII activity in this patient group, we suggest that it is inappropriate to withdraw an epidural catheter when PT-INR or EXTEM-CT are above the levels seen in this study. As vitamin K is relatively non-toxic, we would recommend our current praxis of administering vitamin K and delaying withdrawal of the catheter until test results have normalized (Shearer 2009).

### Fibrinogen levels and ROTEM® FIBTEM do not correlate because of hyperfibrinogenaemia

An important finding in this study is that there was no correlation between fibrinogen levels and thromboelastometric measures of clot stability and that there was a strong hyperfibrinogenaemia. This demonstrates that the postoperative coagulative environment differs from that during operations or haemorrhage: previous work has found low FIBTEM-MCF to reflect low fibrinogen levels (Schochl et al. 2011; Ogawa et al. 2012). We ascribe the hyperfibrinogenaemia to inflammation caused by both the operative insult and the patients’ pre-existing cancer diagnoses (Cole et al. 2008; Fink-Neuboeck et al. 2016; Battistelli et al. 2008) and suggest that it is the most important factor contributing to postoperative hypercoagulation.

### Acquired deficiency of FXIII may be important and is detected by thromboelastometry but not routine tests

Levels of FXIII decreased significantly over the perioperative course and correlated significantly but weakly to ROTEM® EXTEM and FIBTEM-MCF, which is in agreement with previous preclinical and clinical studies: FXIII stabilizes the fibrin clot. This stage of coagulation is not detected by routine tests since it occurs after initiation (Theusinger et al. 2010, 2013; Korte et al. 2009). Since our ROTEM® results indicated postoperative hypercoagulation, we suggest that low FXIII activity was compensated for by the abundances of fibrinogen and platelets. Low FXIII combined with low fibrinogen and platelets could, however, constitute a dangerous coagulative environment for withdrawing an epidural catheter that would not be detected by PT-INR or aPTT. The lowest observed FXIII level in our study was 0.38 kIE/L (38 % of normal) in the patient who was also postoperatively thrombocytopenic. One case series of 63 patients with acquired FXIII deficiency presenting as clinical haemorrhage found FXIII activity to be reduced between 0 and 58 % (Tone et al. 2016). FXIII deficiency should therefore be considered before withdrawal of an epidural catheter in patients with a clinical tendency to bleeding, particularly if thromboelastometry indicates hypocoagulation.

### We saw no evidence of accumulation of LMWH

ROTEM® results did not indicate that an accumulation of LMWH could explain the elevated postoperative aPTT values. In retrospect, we regret that we did not measure anti-factor Xa activity as an additional test to exclude heparin effect. ROTEM® INTEM and HEPTEM can be used to detect heparin in vitro using blood from healthy volunteers (Mittermayr et al. 2005), but we question these tests’ validity in the setting of our study given that HEPTEM-CT was longer than INTEM-CT in many of our patients. We had hypothesized that the cause of accumulation of LMWH would be postoperative relative renal failure, which would not appear to be the case if we choose to believe that serum creatinine is an accurate measure of renal function despite its sources of error (Fliser 2008).

### Platelets and platelet aggregometry

An additional explanation for the inconsistency between routine and viscoelastic tests results is that routine tests do not measure thrombocytosis or the increase in platelet activity seen in this study. Coagulation factor-platelet interactions, particularly in the context of activated inflammation, are complicated: thrombin itself activates intracellular pathways in platelets via the PAR-1 and PAR-4 receptors and platelets bind to fibrinogen in the forming clot such that apparent decreases in protein-mediated coagulation as shown by PT-INR and aPTT may be counteracted not only by hyperfibrinogenaemia but also platelet hyperactivity (Franchi and Angiolillo 2015).

Is there a clinical role for platelet aggregometry when deciding to remove an epidural catheter? Given the correlation between Plc and Multiplate® results and that the postoperative course is characterized by thrombocytosis, we recommend screening with platelet count alone. Situations where platelet aggregometry would be appropriate before withdrawing an epidural catheter include after the administration of platelet inhibitors, for example monitoring ticagrelor’s effect if it had been given because of acute coronary syndrome; or if there was suspicion of platelet dysfunction. Platelet function testing may also be considered in clinical situations that are not following a course of normal recovery: platelet function may be abnormal after traumatic brain injury even in the presence of normal Plc (Nekludov et al. 2007).

### PIVKA-II results suggest a deficiency of vitamin K which is not consistent with the changes in coagulation factor activity

PIVKA-II levels suggest that our patients had a preoperative deficiency in vitamin K which worsened over the perioperative course, which is consistent with previous work from our own research group (Dauti et al. 2015). This is plausible since vitamin K requires bile for absorption in the small intestine: all our patients had pathology of the upper gastrointestinal tract. We note that the normal preoperative albumin levels observed do not suggest malnutrition, but also that albumin has a low sensitivity and specificity for clinical malnutrition and that many of our patients had lost weight (Covinsky et al. 2002). There were no significant decreases in the levels of activity of vitamin K-dependent coagulation factor activities in the group as a whole (see Fig. 5): in fact, FIX and FX actually increased significantly. We were surprised that we did not see significant correlations between PIVKA-II and any tests of coagulation or CRP, other than FXII which actually showed a positive correlation to PIVKA-II levels.

Previous work reaches conflicting conclusions regarding the relationship between malnutrition, deficiency of vitamin K and PT-INR. In one study, a week’s preoperative fast resulted in prolonged postoperative PT and a significant depletion of vitamin K-dependent clotting factors (Moriwaki and Sugiyama 2010). In another study, however, a low vitamin K diet 3 days before and after surgery in patients undergoing non-hepatic gastrointestinal surgery resulted in decreased levels of vitamin K, without any effect on PT (Usui et al. 1990). We would tentatively conclude that the patients in this study were deficient in vitamin K but that the clinical relevance of this is unclear. In addition to this, given that patients with extremely elevated PIVKA-II were not hypocoagulable, we do not think that the PIVKA-II test that we used can be used to identify patients who would benefit from supplementation of vitamin K.

## Conclusions

This study is novel in that we describe patterns of coagulation in the specific context of withdrawing epidural catheters from patients with serious pathology of the upper gastrointestinal tract and who have undergone major surgery. Although PIVKA-II results suggested deficiency of vitamin K, we could not demonstrate a clear correlation between PIVKA-II results and deficits of vitamin K-dependent coagulation factors. There is a clear discrepancy between routine tests, which indicate a tendency towards postoperative hypocoagulation which may be due to postoperative decreases in factors VII and XII, respectively, and thromboelastometry which indicates normo- or hypercoagulability in the postoperative period, explained by thrombocytosis and hyperfibrinogenaemia.

Can our results be used to make clinical praxis safer and more evidence-based? Given the small size of this study, we must be very cautious of drawing conclusions that could affect patient care. First, testing for platelet count remains mandatory due to the risk of transient postoperative thrombocytopenia during the first few days after operation and thereafter heparin-induced thrombocytopenia due to treatment with LMWH. Secondly, the appropriateness of our praxis of administering vitamin K to non-bleeding patients with slightly elevated routine tests of coagulation can certainly be questioned since normal postoperative thrombocytosis and hyperfibrinogenaemia appear to compensate for small relative deficits in coagulation factors: it is clear from this and other studies of postoperative coagulation, that patients who are recovering well after major surgery become hypercoagulable after 3–5 days and that the aPTT and PT-INR in the postoperative setting is generally somewhat higher than in healthy volunteers. Finally, can viscoelstic tests be used to sort patients with mildly elevated routine tests into those whose epidural can safely be removed, and those where withdrawal should be delayed or the coagulopathy treated? Due to thromboelastometry’s low sensitivity for moderate anticoagulation with LMWH and vitamin K antagonists, and the catastrophic nature of spinal haematoma, we would not recommend using normal thromboelastometric results as a carte blanche to ignore routine tests indicative of coagulopathy. Nevertheless without being able to quantify the risk/benefit ratio, we would feel confident withdrawing an epidural catheter from a patient with a PT-INR of 1.5 or aPTT slightly above the reference range after the fourth postoperative day, provided that the patient did not have thrombocytopenia, was recovering well and had no clinical bleeding. It would be imperative to withdraw the catheter following all the usual caveats of the guidelines (Horlocker et al. 2010a; Gogarten et al. 2010; Breivik et al. 2010).

We eagerly await new editions of guidelines for the withdrawal of epidural catheters and expect that there will be a tendency towards more aggressive monitoring of coagulation during the early postoperative period and a more relaxed attitude to screening with PT-INR and aPTT in patients who are recovering well and who do not have risk factors such as renal failure or continued haemorrhage.

### Limitations

This study should be seen as a pilot study examining multifactorial relationships between several aspects of coagulation and has therefore insufficient power to exclude significant patterns. Even accredited tests of coagulation are vulnerable to preanalytical error (Kamal et al. 2007), but thromboelastometry and platelet aggregometry in this study are particularly prone to such errors since they were run by clinicians or medical students. In addition to this, as with most studies investigating very rare complications, we were unable to measure any clinical end-points: the risk of spinal haematoma is so low that a prohibitively expensive multicentre approach would be necessary to prospectively take blood tests from patients with spinal haematoma. We are, however, proponents of prospectively collecting blood samples from patients who have been unfortunate enough to have experienced a coagulative complication of neuraxial blockade in order to run a full battery of routine, whole blood and advanced coagulation tests.

## Additional files

Additional file 1: Table S1. Summary of current international guidelines on routine coagulation results and platelet count for insertion and withdrawal of epidural catheters. (PDF 118 kb)
Additional file 2: Table S2. Assays taken, reference intervals, apparatuses and reagents used. (PDF 91 kb)
Additional file 3: Statistical calculations. Output from R showing results from all the statistical calculations used in the article. (PDF 270 kb)
Additional file 4: Case of patient with thrombocytopenia and low factor XIII. Table showing pre- and postoperative laboratory results for this patient. (PDF 170 kb)
Additional file 5: Raw data with explanation of field codes in first lines. (TXT 18 kb)

## Abbreviations

aPTT: Activated partial thromboplastin time
CRP: C-reactive protein
F: Coagulation factor(s)
LMWH: Low molecular weight heparin
PIVKA: Proteins induced by vitamin K absence
Plc: Platelet count
PT-INR: Prothrombin time-international normalized ratio
ROTEM®: Rotational thromboelastometry
