# Supplementary material for: Coagulative safety of epidural catheters after major upper gastrointestinal surgery: advanced and routine coagulation analysis in 38 patients
Source: Perioper Med (Lond). 2016 Oct 18;5:28. doi: 10.1186/s13741-016-0053-0 (PMC5067910; doi:10.1186/s13741-016-0053-0)
Supplement: Additional file 1: Table S1. — Summary of current international guidelines on routine coagulation results and platelet count for insertion and withdrawal of epidural catheters. (PDF 118 kb) [file 13741_2016_53_MOESM1_ESM.pdf]

Supplement 1: Major international guidelines from 2010: summary of specific requirements of routine tests of coagulation for insertion and withdrawal of epidural catheters

<table>
<tr><th></th><th>Insertion of epidural</th><th>Withdrawal</th></tr>
<tr><td colspan="3">PT-INR</td></tr>
<tr><td>SSAI (Scandinavian Society for Anaesthesia and Intensive Care). [10] *</td><td colspan="2">Comfort: ≤1.2.
Morbidity: <1.6.
Mortality: <1.8.</td></tr>
<tr><td>ASRA (American Society of Anaesthesia) [3] *</td><td>‘Normalized’ and 4-5 days after warfarin discontinued.</td><td><1.5 although 1.5-3 sometimes acceptable ‘with caution’</td></tr>
<tr><td>ESA (European Society of Anaesthesia) [8]</td><td>≤1.4</td><td>Warfarin should be administered only when catheter has been removed.</td></tr>
<tr><td colspan="3">aPTT</td></tr>
<tr><td>SSAI</td><td colspan="2">‘Should be within the normal range’</td></tr>
<tr><td>ASRA</td><td colspan="2">“It is not necessary to routinely check the aPTT or platelet count, unless the clinician is concerned about changes in these values after prolonged administration or in patients with many comorbidities that might influence the pharmacologic expression of subcutaneous UFH.”</td></tr>
<tr><td>ESA</td><td>‘aPTT should have normalized (after heparin).’</td><td>‘Normal aPTT’</td></tr>
<tr><td colspan="3">Platelet count (x10^{6})</td></tr>
<tr><td>SSAI</td><td colspan="2">Comfort: >100
Morbidity: >80
Mortality: >50</td></tr>
<tr><td>ASRA</td><td colspan="2">“Because heparin-induced thrombocytopenia may occur during heparin administration, we recommend that patients receiving heparin for more than 4 days have a platelet count assessed before neuraxial block and catheter removal.”</td></tr>
<tr><td>ESA</td><td colspan="2">Platelet count should be monitored if patient has received UFH or LMWH for 5 days or more. No threshold given.</td></tr>
</table>

*Note that the Nordic guidelines cover ’disturbed haemostasis’ while the ASRA and ESA Guidelines relate to patients on anticoagulative drugs. The Scandinavian guidelines recommend a ’benefit analysis’ of epidural catheterization and removal of epidural catheters: the requirements for test results are more stringent in procedures that are only for comfort compared to procedures that reduce the risk for morbidity or mortality.
