# Supplementary material for: Coagulative safety of epidural catheters after major upper gastrointestinal surgery: advanced and routine coagulation analysis in 38 patients
Source: Perioper Med (Lond). 2016 Oct 18;5:28. doi: 10.1186/s13741-016-0053-0 (PMC5067910; doi:10.1186/s13741-016-0053-0)
Supplement: Additional file 2: Table S2. — Assays taken, reference intervals, apparatuses and reagents used. (PDF 91 kb) [file 13741_2016_53_MOESM2_ESM.pdf]

S2 Supplementary Table: assays taken, reference intervals, apparatuses and reagents used

Apparatus/method	Reagent	Parameter	Reference interval
ROTEM according to manufacturer's instructions (Pentapharm, Munich, Germany). Reversal of coagulation using calcium chloride (StarTEM® reagent).	EXTEM: contains tissue factor (TF), i.e. initiates the extrinsic pathway	MCF (Maximal clot firmness)	50-72 mm
		CT (Clotting time)	38-79 mm
	INTEM: contains ellagic acid, i.e. initiates the intrinsic pathway	MCF	50-72 mm
		CT	100-240 s
	FIBTEM: contains TF and cytochalasin D, a platelet inhibitor.	MCF	9-25 mm
	HEPTEM: contains ellagic acid and heparinase.	MCF	50-72 mm
		CT	100-240 s
Multiplate® (Dynabyte GmbH, Münich, Germany)	ADPtest	AUC (Area under curve	57-113 U
	COLtest		72-125 U
	TRAPtest		84-128 U
	ASPItest		71-115 U
BCS-XP Coagulation analyzer (Siemens‡)	Thromborel S‡	P-FII	0.70–1.50 kIE/L
	Thromborel S‡	P-FVII	0.60–1.60 kIE/L
	Thromborel S‡	P-FX	0.70–1.52 kIE/L
	PTT-AutomateΔ	P-FIX	0.70–1.30 kIE/L
	PTT-AutomateΔ	P-FXI	0.60–1.30 kIE/L
	PTT-AutomateΔ	P-FXII	1.07–1.50 kIE/L
	Berichrome FXIII‡	P-FXIII	0.70–1.40 kIE/L
	Owren's PT – a combined thromboplastin reagent (MediRox, Nyköping, Sweden)*	PT-INR	0.9-1.1
	Actin FSL (Siemens‡)	aPTT	26-33s
CS-5100 (Siemens Healthcare Diagnostics, Marburg, Germany)	Dade Thrombin	P-Fibrinogen	2.0-4.0 mg/mL
	Medirox D-dimer	D-dimer	<0.25mg/L
	Cobas GTT (Roche)	Gamma-Glutamyltransferase (GT)	0.2-1.9µkat/L
	Cobas CRP-L3 (Roche)	C-reactive protein (CRP)	<3.0mg/L
	BilT3 Cobas (Roche)	Bilirubin	5-25µmol/L
	Cobas ALP2 (Roche)	Alkaline phosphatase (ALP)	0.6-1.8µkat/L
	CREP2 Cobas (Roche)	Creatinine	45-90µmol/L
Radiometer medical ApS, Brønshøj, Denmark		Haemoglobin (Hb)	Men: 134-170; women: 117-153 g/L.
Asserachrom ® PIVKA-II kit (StagoΔ).	An enzyme-linked immunosorbent assay using monoclonal antibody p1-2-b9.	PIVKA-II (Protein induced by vitamin K absence)	<2.0mg/L
Sysmex XE 5000 cell counter†		Platelet count (Plc)	Women: 165–387; men: 145–348 x 10^9/L.

*assay certified by Equalis, Uppsala, Sweden. ‡Siemens Healthcare Diagnostics, Marburg, Germany. †Sysmex Corp., Kobe, Japan. ΔStago, Asnières-sur-Seine, France.
