# Supplementary material for: Coagulative safety of epidural catheters after major upper gastrointestinal surgery: advanced and routine coagulation analysis in 38 patients
Source: Perioper Med (Lond). 2016 Oct 18;5:28. doi: 10.1186/s13741-016-0053-0 (PMC5067910; doi:10.1186/s13741-016-0053-0)
Supplement: Additional file 3: — Statistical calculations. Output from R showing results from all the statistical calculations used in the article. (PDF 270 kb) [file 13741_2016_53_MOESM3_ESM.pdf]

```
R version 3.2.4 (2016-03-10) -- "Very Secure Dishes"
Copyright (C) 2016 The R Foundation for Statistical Computing
Platform: x86_64-apple-darwin13.4.0 (64-bit)

R is free software and comes with ABSOLUTELY NO WARRANTY.
You are welcome to redistribute it under certain conditions.
Type 'license()' or 'licence()' for distribution details.

  Natural language support but running in an English locale

R is a collaborative project with many contributors.
Type 'contributors()' for more information and
'citation()' on how to cite R or R packages in publications.

Type 'demo()' for some demos, 'help()' for on-line help, or
'help.start()' for an HTML browser interface to help.
Type 'q()' to quit R.

During startup - Warning messages:
1: Setting LC_CTYPE failed, using "C"
2: Setting LC_COLLATE failed, using "C"
3: Setting LC_TIME failed, using "C"
4: Setting LC_MESSAGES failed, using "C"
5: Setting LC_MONETARY failed, using "C"
[R.app GUI 1.67 (7152) x86_64-apple-darwin13.4.0]

WARNING: You're using a non-UTF8 locale, therefore only ASCII characters will
work.
Please read R for Mac OS X FAQ (see Help) section 9 and adjust your system
preferences accordingly.
[Workspace restored from /Users/OwainThomas/Dropbox/RESEARCH/EPI/Statistik EPI
2014/.RData]
[History restored from /Users/OwainThomas/Dropbox/RESEARCH/EPI/Statistik EPI
2014/.Rapp.history]

2016-07-06 14:02:50.954 R[5666:3360988] *** WARNING: Method
convertPointFromBase: in class NSView is deprecated on 10.7 and later. It
should not be used in new applications.
> epi<-read.table(file="epiresultat.txt", header=TRUE,dec=",")
> epi$preopHEPTEMminusINTEMmcf<-epi$Prov1heptemMCF-epi$Prov1intemMCF
> epi$postopHEPTEMminusINTEMmcf<-epi$Prov2heptemMCF-epi$Prov2intemMCF
> mean(epi$PatAge)
[1] 70.23684
> sd(epi$PatAge)
[1] 6.94548
> min(epi$PatAge)
[1] 57
> max(epi$PatAge)
[1] 82
>
> sum(epi$PatSex)
[1] 27
> #MCF
> boxplot(epi$Prov1extemMCF,epi$Prov2extemMCF,-100, epi$Prov1fibtemMCF,epi
$Prov2fibtemMCF,-100, epi$Prov1intemMCF,epi$Prov2intemMCF,-100, epi
```

```
$Prov1heptemMCF,epi$Prov2heptemMCF,-100,epi$preopHEPTEMminusINTEMmcf,epi
$postopHEPTEMminusINTEMmcf, horizontal=FALSE, na.rm=TRUE, axes=TRUE, main="Pre-
and postoperative ROTEM MCF Values",cex.main=1.65,
ylim=c(-5,95),cex.axis=1.5,cex.lab=1.45, ylab="Maximum clot firmness (MCF) in
mm", boxwex=1, col=c(24,0,0,24,0,0,24,0,0,24,0,0,24,0), lty=1)
>  text(x=1.5,y=90, "EXTEM", pos=3, cex=1.2)
>  text(x=4.5,y=90, "FIBTEM", pos=3, cex=1.2)
>  text(x=7.5,y=90, "INTEM", pos=3, cex=1.2)
>  text(x=10.5,y=90, "HEPTEM", pos=3, cex=1.2)
>  text(x=13.5,y=90, "HEPTEM -", pos=3, cex=1.2)
>  text(x=13.5,y=85, "INTEM", pos=3, cex=1.2)
>  legend(0.7,7, c("Preoperative","Postoperative"), cex= 1,fill=c(24,0))
>  legend(5.7,5,lty=3,lwd=2,c("Reference range"))
>  arrows(0.4,50,,72,code=0,lwd=2,lty=3)
>  arrows(3.4,9,,25,code=0,lwd=2,lty=3)
>  arrows(6.4,50,,72,code=0,lwd=2,lty=3)
>  arrows(9.4,50,,72,code=0,lwd=2,lty=3)
>
> #hist(epi$Prov1extemMCF)
> #hist(epi$Prov2extemMCF)
> mean(epi$Prov1extemMCF, na.rm=TRUE)
[1] 64.05263
> sd(epi$Prov1extemMCF, na.rm=TRUE)
[1] 7.897818
> mean(epi$Prov2extemMCF, na.rm=TRUE)
[1] 71.66667
> sd(epi$Prov2extemMCF, na.rm=TRUE)
[1] 6.111806
>
> t.test(epi$Prov1extemMCF,epi$Prov2extemMCF,alternative =
c("two.sided"),paired = TRUE)

	Paired t-test

data:  epi$Prov1extemMCF and epi$Prov2extemMCF
t = -5.0837, df = 32, p-value = 1.558e-05
alternative hypothesis: true difference in means is not equal to 0
95 percent confidence interval:
 -11.247908  -4.812698
sample estimates:
mean of the differences
              -8.030303

> wilcox.test(epi$Prov1extemMCF,epi$Prov2extemMCF,alternative =
c("two.sided"),paired = TRUE)

	Wilcoxon signed rank test with continuity correction

data:  epi$Prov1extemMCF and epi$Prov2extemMCF
V = 40, p-value = 7.779e-05
alternative hypothesis: true location shift is not equal to 0

Warning messages:
1: In wilcox.test.default(epi$Prov1extemMCF, epi$Prov2extemMCF, alternative =
c("two.sided"),  :
  cannot compute exact p-value with ties
```

```
2: In wilcox.test.default(epi$Prov1extemMCF, epi$Prov2extemMCF, alternative =
c("two.sided"),  :
  cannot compute exact p-value with zeroes
> x<-1
> y<-85
> arrows(x,y,x+1,y, length=0.05, angle=90, code=1)
> arrows(x,y,x+1,y, length=0.05, angle=90, code=2)
> text(x+0.5,y-3,"**",pos=3, cex=1.4)
>
> #hist(epi$Prov1fibtemMCF)
> #hist(epi$Prov2fibtemMCF)
> mean(epi$Prov1fibtemMCF, na.rm=TRUE)
[1] 20.08108
> sd(epi$Prov1fibtemMCF, na.rm=TRUE)
[1] 7.166195
> mean(epi$Prov2fibtemMCF, na.rm=TRUE)
[1] 33.72727
> sd(epi$Prov2fibtemMCF, na.rm=TRUE)
[1] 9.155848
> t.test(epi$Prov1fibtemMCF,epi$Prov2fibtemMCF,alternative =
c("two.sided"),paired = TRUE)

    Paired t-test

data:  epi$Prov1fibtemMCF and epi$Prov2fibtemMCF
t = -6.8746, df = 31, p-value = 1.048e-07
alternative hypothesis: true difference in means is not equal to 0
95 percent confidence interval:
 -17.788756  -9.648744
sample estimates:
mean of the differences
              -13.71875

> wilcox.test(epi$Prov1fibtemMCF,epi$Prov2fibtemMCF,alternative =
c("two.sided"),paired = TRUE)

    Wilcoxon signed rank test with continuity correction

data:  epi$Prov1fibtemMCF and epi$Prov2fibtemMCF
V = 21, p-value = 5.753e-06
alternative hypothesis: true location shift is not equal to 0

Warning message:
In wilcox.test.default(epi$Prov1fibtemMCF, epi$Prov2fibtemMCF, alternative =
c("two.sided"),  :
  cannot compute exact p-value with ties
> x<-4
> y<-85
> arrows(x,y,x+1,y, length=0.05, angle=90, code=1)
> arrows(x,y,x+1,y, length=0.05, angle=90, code=2)
> text(x+0.5,y-3,"**",pos=3, cex=1.4)
>
> #hist(epi$Prov1intemMCF)
> #hist(epi$Prov2intemMCF)
> mean(epi$Prov1intemMCF, na.rm=TRUE)
[1] 63.28947
```

```
> sd(epi$Prov1intemMCF, na.rm=TRUE)
[1] 5.862883
> mean(epi$Prov2intemMCF, na.rm=TRUE)
[1] 69.54545
> sd(epi$Prov2intemMCF, na.rm=TRUE)
[1] 8.551648
> t.test(epi$Prov1intemMCF,epi$Prov2intemMCF,alternative =
c("two.sided"),paired = TRUE)

	Paired t-test

data:  epi$Prov1intemMCF and epi$Prov2intemMCF
t = -3.8044, df = 32, p-value = 0.0006047
alternative hypothesis: true difference in means is not equal to 0
95 percent confidence interval:
 -9.956928 -3.012769
sample estimates:
mean of the differences
              -6.484848

> wilcox.test(epi$Prov1intemMCF,epi$Prov2intemMCF,alternative =
c("two.sided"),paired = TRUE)

	Wilcoxon signed rank test with continuity correction

data:  epi$Prov1intemMCF and epi$Prov2intemMCF
V = 60.5, p-value = 0.0002462
alternative hypothesis: true location shift is not equal to 0

Warning messages:
1: In wilcox.test.default(epi$Prov1intemMCF, epi$Prov2intemMCF, alternative =
c("two.sided"),  :
  cannot compute exact p-value with ties
2: In wilcox.test.default(epi$Prov1intemMCF, epi$Prov2intemMCF, alternative =
c("two.sided"),  :
  cannot compute exact p-value with zeroes
> x<-7
> y<-85
> arrows(x,y,x+1,y, length=0.05, angle=90, code=1)
> arrows(x,y,x+1,y, length=0.05, angle=90, code=2)
> text(x+0.5,y-3,"**",pos=3, cex=1.4)
>
> #hist(epi$Prov1heptemMCF)
> #hist(epi$Prov2heptemMCF)
> mean(epi$Prov1heptemMCF, na.rm=TRUE)
[1] 62.18421
> sd(epi$Prov1heptemMCF, na.rm=TRUE)
[1] 5.834915
> mean(epi$Prov2heptemMCF, na.rm=TRUE)
[1] 69.40625
> sd(epi$Prov2heptemMCF, na.rm=TRUE)
[1] 6.399896
> t.test(epi$Prov1heptemMCF,epi$Prov2heptemMCF,alternative =
c("two.sided"),paired = TRUE)

	Paired t-test
```

```
data:  epi$Prov1heptemMCF and epi$Prov2heptemMCF
t = -6.3687, df = 31, p-value = 4.317e-07
alternative hypothesis: true difference in means is not equal to 0
95 percent confidence interval:
 -9.365446 -4.822054
sample estimates:
mean of the differences
               -7.09375

> wilcox.test(epi$Prov1heptemMCF,epi$Prov2heptemMCF,alternative =
c("two.sided"),paired = TRUE)

    Wilcoxon signed rank test with continuity correction

data:  epi$Prov1heptemMCF and epi$Prov2heptemMCF
V = 21.5, p-value = 2.327e-05
alternative hypothesis: true location shift is not equal to 0

Warning messages:
1: In wilcox.test.default(epi$Prov1heptemMCF, epi$Prov2heptemMCF, alternative =
c("two.sided"),  :
  cannot compute exact p-value with ties
2: In wilcox.test.default(epi$Prov1heptemMCF, epi$Prov2heptemMCF, alternative =
c("two.sided"),  :
  cannot compute exact p-value with zeroes
> x<-10
> y<-85
> arrows(x,y,x+1,y, length=0.05, angle=90, code=1)
> arrows(x,y,x+1,y, length=0.05, angle=90, code=2)
> text(x+0.5,y-3,"**",pos=3, cex=1.4)
>
> mean(epi$preopHEPTEMminusINTEMmcf, na.rm=TRUE)
[1] -1.105263
> sd(epi$preopHEPTEMminusINTEMmcf, na.rm=TRUE)
[1] 1.752056
> mean(epi$postopHEPTEMminusINTEMmcf, na.rm=TRUE)
[1] -0.15625
> sd(epi$postopHEPTEMminusINTEMmcf, na.rm=TRUE)
[1] 6.687057
> #hist(epi$preopHEPTEMminusINTEMmcf)
> epi$preopHEPTEMminusINTEMmcf
 [1]  0 -2 -3 -2  2 -2  0  1 -1 -4 -4  2 -1 -3 -3  1 -3 -2  1 -4  0  0  0 -1  0
[26]  0  1 -2 -4 -1  0 -1  1 -4 -1 -1 -2  0
> #hist(epi$postopHEPTEMminusINTEMmcf)
>
> t.test(epi$preopHEPTEMminusINTEMmcf,epi$postopHEPTEMminusINTEMmcf,alternative
= c("two.sided"),paired = TRUE)

    Paired t-test

data:  epi$preopHEPTEMminusINTEMmcf and epi$postopHEPTEMminusINTEMmcf
t = -0.55375, df = 31, p-value = 0.5837
alternative hypothesis: true difference in means is not equal to 0
95 percent confidence interval:
 -3.073271  1.760771
```

```
sample estimates:
mean of the differences
               -0.65625

> wilcox.test(epi$preopHEPTEMminusINTEMmcf,epi
$postopHEPTEMminusINTEMmcf,alternative = c("two.sided"),paired = TRUE)

    Wilcoxon signed rank test with continuity correction

data:  epi$preopHEPTEMminusINTEMmcf and epi$postopHEPTEMminusINTEMmcf
V = 156.5, p-value = 0.5819
alternative hypothesis: true location shift is not equal to 0

Warning messages:
1: In wilcox.test.default(epi$preopHEPTEMminusINTEMmcf, epi
$postopHEPTEMminusINTEMmcf,  :
  cannot compute exact p-value with ties
2: In wilcox.test.default(epi$preopHEPTEMminusINTEMmcf, epi
$postopHEPTEMminusINTEMmcf,  :
  cannot compute exact p-value with zeroes
>
>
>
> #CT
> boxplot(epi$Prov1extemCT,epi$Prov2extemCT,-1000, epi$Prov1intemCT,epi
$Prov2intemCT,-1000, epi$Prov1heptemCT,epi$Prov2heptemCT,-1000,epi
$preopINTEMminusHEPTEMct,epi$postopINTEMminusHEPTEMct, horizontal=FALSE,
na.rm=TRUE, axes=TRUE, main="Pre- and postoperative ROTEM CT
Values",cex.main=1.65,cex.axis=1.5,cex.lab=1.45, ylab="Clotting time (CT) in
s", boxwex=1, col=c(24,0,0,24,0,0,24,0,0,24,0,0,24,0), ylim=c(-102,400),lty=1)
Warning messages:
1: In is.na(x) : is.na() applied to non-(list or vector) of type 'NULL'
2: In is.na(x) : is.na() applied to non-(list or vector) of type 'NULL'
3: In is.na(x) : is.na() applied to non-(list or vector) of type 'NULL'
4: In is.na(x) : is.na() applied to non-(list or vector) of type 'NULL'
5: In is.na(x) : is.na() applied to non-(list or vector) of type 'NULL'
6: In is.na(x) : is.na() applied to non-(list or vector) of type 'NULL'
> text(x=1.5,y=380, "EXTEM", pos=3, cex=1.2)
> text(x=4.5,y=380, "INTEM", pos=3, cex=1.2)
> text(x=7.5,y=380, "HEPTEM", pos=3, cex=1.2)
> text(x=10.5,y=380, "INTEM -", pos=3, cex=1.2)
> text(x=10.5,y=355, "HEPTEM", pos=3, cex=1.2)
> legend(0.7,-20, c("Preoperative","Postoperative"), cex= 1,fill=c(24,0))
>
> legend(4.7,-25,lty=3,lwd=2,c("Reference range"))
> arrows(0.4,38,,79,code=0,lwd=2,lty=3)
> arrows(3.4,100,,240,code=0,lwd=2,lty=3)
> arrows(6.4,100,,240,code=0,lwd=2,lty=3)
>
> mean(epi$Prov1extemCT, na.rm=TRUE)
[1] 47.52632
> sd(epi$Prov1extemCT, na.rm=TRUE)
[1] 13.90652
> mean(epi$Prov2extemCT, na.rm=TRUE)
[1] 48.12121
> sd(epi$Prov2extemCT, na.rm=TRUE)
```

```
[1] 12.19825
>
> mean(epi$Prov1intemCT, na.rm=TRUE)
[1] 175.9474
> sd(epi$Prov1intemCT, na.rm=TRUE)
[1] 46.48158
> mean(epi$Prov2intemCT, na.rm=TRUE)
[1] 165.0303
> sd(epi$Prov2intemCT, na.rm=TRUE)
[1] 42.96982
>
> mean(epi$Prov1heptemCT, na.rm=TRUE)
[1] 187.7632
> sd(epi$Prov1heptemCT, na.rm=TRUE)
[1] 49.63675
> mean(epi$Prov2heptemCT, na.rm=TRUE)
[1] 181.7273
> sd(epi$Prov2heptemCT, na.rm=TRUE)
[1] 45.23016
>
> t.test(epi$Prov1extemCT,epi$Prov2extemCT,alternative = c("two.sided"),paired
= TRUE)

	Paired t-test

data:  epi$Prov1extemCT and epi$Prov2extemCT
t = -0.71131, df = 32, p-value = 0.482
alternative hypothesis: true difference in means is not equal to 0
95 percent confidence interval:
 -7.376024  3.557842
sample estimates:
mean of the differences
              -1.909091

> wilcox.test(epi$Prov1extemCT,epi$Prov2extemCT,alternative =
c("two.sided"),paired = TRUE)

	Wilcoxon signed rank test with continuity correction

data:  epi$Prov1extemCT and epi$Prov2extemCT
V = 178.5, p-value = 0.1117
alternative hypothesis: true location shift is not equal to 0

Warning messages:
1: In wilcox.test.default(epi$Prov1extemCT, epi$Prov2extemCT, alternative =
c("two.sided"),  :
  cannot compute exact p-value with ties
2: In wilcox.test.default(epi$Prov1extemCT, epi$Prov2extemCT, alternative =
c("two.sided"),  :
  cannot compute exact p-value with zeroes
> mean(epi$Prov1extemCT, na.rm=TRUE)
[1] 47.52632
> sd(epi$Prov1intemCT, na.rm=TRUE)
[1] 46.48158
>
> t.test(epi$Prov1intemCT,epi$Prov2intemCT,alternative = c("two.sided"),paired
```

```
= TRUE)

    Paired t-test

data:  epi$Prov1intemCT and epi$Prov2intemCT
t = 1.462, df = 32, p-value = 0.1535
alternative hypothesis: true difference in means is not equal to 0
95 percent confidence interval:
 -6.07791 36.98700
sample estimates:
mean of the differences 
               15.45455 

> wilcox.test(epi$Prov1intemCT,epi$Prov2intemCT,alternative =
c("two.sided"),paired = TRUE)

    Wilcoxon signed rank test with continuity correction

data:  epi$Prov1intemCT and epi$Prov2intemCT
V = 371.5, p-value = 0.1058
alternative hypothesis: true location shift is not equal to 0

Warning message:
In wilcox.test.default(epi$Prov1intemCT, epi$Prov2intemCT, alternative =
c("two.sided"),  :
  cannot compute exact p-value with ties
> mean(epi$Prov1extemCT, na.rm=TRUE)
[1] 47.52632
> 
> t.test(epi$Prov1heptemCT,epi$Prov2heptemCT,alternative =
c("two.sided"),paired = TRUE)

    Paired t-test

data:  epi$Prov1heptemCT and epi$Prov2heptemCT
t = 0.69247, df = 32, p-value = 0.4936
alternative hypothesis: true difference in means is not equal to 0
95 percent confidence interval:
 -17.23871  34.99629
sample estimates:
mean of the differences 
               8.878788 

> wilcox.test(epi$Prov1heptemCT,epi$Prov2heptemCT,alternative =
c("two.sided"),paired = TRUE)

    Wilcoxon signed rank test with continuity correction

data:  epi$Prov1heptemCT and epi$Prov2heptemCT
V = 334.5, p-value = 0.3391
alternative hypothesis: true location shift is not equal to 0

Warning message:
In wilcox.test.default(epi$Prov1heptemCT, epi$Prov2heptemCT, alternative =
c("two.sided"),  :
  cannot compute exact p-value with ties
```

```
>
>
> t.test(epi$Prov1CT,epi$Prov2fibtemCT,alternative = c("two.sided"),paired =
TRUE)
Error in t.test.default(epi$Prov1CT, epi$Prov2fibtemCT, alternative =
c("two.sided"),  :
  not enough 'x' observations
> wilcox.test(epi$Prov1fibtemCT,epi$Prov2fibtemCT,alternative =
c("two.sided"),paired = TRUE)

	Wilcoxon signed rank test with continuity correction

data:  epi$Prov1fibtemCT and epi$Prov2fibtemCT
V = 165, p-value = 0.1055
alternative hypothesis: true location shift is not equal to 0

Warning messages:
1: In wilcox.test.default(epi$Prov1fibtemCT, epi$Prov2fibtemCT, alternative =
c("two.sided"),  :
  cannot compute exact p-value with ties
2: In wilcox.test.default(epi$Prov1fibtemCT, epi$Prov2fibtemCT, alternative =
c("two.sided"),  :
  cannot compute exact p-value with zeroes
>
>
>
> t.test(epi$Prov1intemCT,epi$Prov2intemCT,alternative = c("two.sided"),paired
= TRUE)

	Paired t-test

data:  epi$Prov1intemCT and epi$Prov2intemCT
t = 1.462, df = 32, p-value = 0.1535
alternative hypothesis: true difference in means is not equal to 0
95 percent confidence interval:
 -6.07791 36.98700
sample estimates:
mean of the differences
               15.45455

> wilcox.test(epi$Prov1intemCT,epi$Prov2intemCT,alternative =
c("two.sided"),paired = TRUE)

	Wilcoxon signed rank test with continuity correction

data:  epi$Prov1intemCT and epi$Prov2intemCT
V = 371.5, p-value = 0.1058
alternative hypothesis: true location shift is not equal to 0

Warning message:
In wilcox.test.default(epi$Prov1intemCT, epi$Prov2intemCT, alternative =
c("two.sided"),  :
  cannot compute exact p-value with ties
>
>
>
```

```
> t.test(epi$Prov1heptemCT,epi$Prov2heptemCT,alternative =
c("two.sided"),paired = TRUE)

	Paired t-test

data:  epi$Prov1heptemCT and epi$Prov2heptemCT
t = 0.69247, df = 32, p-value = 0.4936
alternative hypothesis: true difference in means is not equal to 0
95 percent confidence interval:
 -17.23871  34.99629
sample estimates:
mean of the differences
               8.878788

> wilcox.test(epi$Prov1heptemCT,epi$Prov2heptemCT,alternative =
c("two.sided"),paired = TRUE)

	Wilcoxon signed rank test with continuity correction

data:  epi$Prov1heptemCT and epi$Prov2heptemCT
V = 334.5, p-value = 0.3391
alternative hypothesis: true location shift is not equal to 0

Warning message:
In wilcox.test.default(epi$Prov1heptemCT, epi$Prov2heptemCT, alternative =
c("two.sided"),  :
  cannot compute exact p-value with ties
>
>
> t.test(epi$preopINTEMminusHEPTEMct,epi$postopINTEMminusHEPTEMct,alternative =
c("two.sided"),paired = TRUE)
Error in t.test.default(epi$preopINTEMminusHEPTEMct, epi
$postopINTEMminusHEPTEMct,  :
  'y' is missing for paired test
> wilcox.test(epi$preopINTEMminusHEPTEMct,epi
$postopINTEMminusHEPTEMct,alternative = c("two.sided"),paired = TRUE)
Error in wilcox.test.default(epi$preopINTEMminusHEPTEMct, epi
$postopINTEMminusHEPTEMct,  :
  'x' must be numeric
>
>
>
> #MultiplateADP
> boxplot(epi$Prov1multiplateADP,epi$Prov2multiplateADP,-100, epi
$Prov1multiplateCOL,epi$Prov2multiplateCOL,-100, epi$Prov1multiplateTRAP,epi
$Prov2multiplateTRAP,-100, epi$Prov1multiplateASPI,epi$Prov2multiplateASPI,
horizontal=FALSE, na.rm=TRUE, axes=TRUE, main="Pre- and postoperative
Multiplate Values",cex.main=1.65, ylim=c(-20,180),cex.axis=1.5,cex.lab=1.45,
ylab="Area Under Curve (AUC)", boxwex=1,
col=c(24,0,0,24,0,0,24,0,0,24,0,0,24,0), lty=1)
> text(x=1.5,y=173, "ADP", pos=3, cex=1.2)
> text(x=4.5,y=173, "COL", pos=3, cex=1.2)
> text(x=7.5,y=173, "TRAP", pos=3, cex=1.2)
> text(x=10.5,y=173, "ASPI", pos=3, cex=1.2)
>
> legend(0.7,0, c("Preoperative","Postoperative"), cex= 1,fill=c(24,0))
```

```
> legend(5.7,0,lty=3,lwd=2,c("Reference range"))
> arrows(0.4,57,,113,code=0,lwd=2,lty=3)
> arrows(3.4,72,,125,code=0,lwd=2,lty=3)
> arrows(6.4,84,,128,code=0,lwd=2,lty=3)
> arrows(9.4,71,,115,code=0,lwd=2,lty=3)
>
> t.test(epi$Prov1multiplateADP,epi$Prov2multiplateADP,alternative =
c("two.sided"),paired = TRUE)

    Paired t-test

data:  epi$Prov1multiplateADP and epi$Prov2multiplateADP
t = -1.5086, df = 32, p-value = 0.1412
alternative hypothesis: true difference in means is not equal to 0
95 percent confidence interval:
 -23.146033   3.449064
sample estimates:
mean of the differences
              -9.848485

> wilcox.test(epi$Prov1multiplateADP,epi$Prov2multiplateADP,alternative =
c("two.sided"),paired = TRUE)

    Wilcoxon signed rank test with continuity correction

data:  epi$Prov1multiplateADP and epi$Prov2multiplateADP
V = 200, p-value = 0.235
alternative hypothesis: true location shift is not equal to 0

Warning messages:
1: In wilcox.test.default(epi$Prov1multiplateADP, epi$Prov2multiplateADP,  :
  cannot compute exact p-value with ties
2: In wilcox.test.default(epi$Prov1multiplateADP, epi$Prov2multiplateADP,  :
  cannot compute exact p-value with zeroes
>
> t.test(epi$Prov1multiplateCOL,epi$Prov2multiplateCOL,alternative =
c("two.sided"),paired = TRUE)

    Paired t-test

data:  epi$Prov1multiplateCOL and epi$Prov2multiplateCOL
t = -5.1807, df = 32, p-value = 1.175e-05
alternative hypothesis: true difference in means is not equal to 0
95 percent confidence interval:
 -45.97478 -20.02522
sample estimates:
mean of the differences
                    -33

> wilcox.test(epi$Prov1multiplateCOL,epi$Prov2multiplateCOL,alternative =
c("two.sided"),paired = TRUE)

    Wilcoxon signed rank test with continuity correction

data:  epi$Prov1multiplateCOL and epi$Prov2multiplateCOL
V = 37.5, p-value = 2.372e-05
```

```
alternative hypothesis: true location shift is not equal to 0

Warning messages:
1: In wilcox.test.default(epi$Prov1multiplateCOL, epi$Prov2multiplateCOL,  :
  cannot compute exact p-value with ties
2: In wilcox.test.default(epi$Prov1multiplateCOL, epi$Prov2multiplateCOL,  :
  cannot compute exact p-value with zeroes
> 
> x<-4
> y<-170
> arrows(x,y,x+1,y, length=0.05, angle=90, code=1)
> arrows(x,y,x+1,y, length=0.05, angle=90, code=2)
> text(x+0.5,y-6,"**",pos=3, cex=1.4)
> 
> t.test(epi$Prov1multiplateTRAP,epi$Prov2multiplateTRAP,alternative = 
c("two.sided"),paired = TRUE)

	Paired t-test

data:  epi$Prov1multiplateTRAP and epi$Prov2multiplateTRAP
t = -1.0839, df = 32, p-value = 0.2865
alternative hypothesis: true difference in means is not equal to 0
95 percent confidence interval:
 -24.430100   7.460403
sample estimates:
mean of the differences 
              -8.484848 

> wilcox.test(epi$Prov1multiplateTRAP,epi$Prov2multiplateTRAP,alternative = 
c("two.sided"),paired = TRUE)

	Wilcoxon signed rank test with continuity correction

data:  epi$Prov1multiplateTRAP and epi$Prov2multiplateTRAP
V = 231.5, p-value = 0.3861
alternative hypothesis: true location shift is not equal to 0

Warning message:
In wilcox.test.default(epi$Prov1multiplateTRAP, epi$Prov2multiplateTRAP,  :
  cannot compute exact p-value with ties
> 
> t.test(epi$Prov1multiplateASPI,epi$Prov2multiplateASPI,alternative = 
c("two.sided"),paired = TRUE)

	Paired t-test

data:  epi$Prov1multiplateASPI and epi$Prov2multiplateASPI
t = -3.1759, df = 32, p-value = 0.003298
alternative hypothesis: true difference in means is not equal to 0
95 percent confidence interval:
 -37.801125  -8.259481
sample estimates:
mean of the differences 
               -23.0303 

> wilcox.test(epi$Prov1multiplateASPI,epi$Prov2multiplateASPI,alternative = 
```

```
c("two.sided"),paired = TRUE)

	Wilcoxon signed rank test with continuity correction

data:  epi$Prov1multiplateASPI and epi$Prov2multiplateASPI
V = 124.5, p-value = 0.005454
alternative hypothesis: true location shift is not equal to 0

Warning message:
In wilcox.test.default(epi$Prov1multiplateASPI, epi$Prov2multiplateASPI,  :
  cannot compute exact p-value with ties
> x<-10
> y<-170
> arrows(x,y,x+1,y, length=0.05, angle=90, code=1)
> arrows(x,y,x+1,y, length=0.05, angle=90, code=2)
> text(x+0.5,y-6,"**",pos=3, cex=1.4)
>
> mean(epi$Prov1multiplateADP, na.rm=TRUE)
[1] 68.10526
> sd(epi$Prov1multiplateADP, na.rm=TRUE)
[1] 21.04023
> mean(epi$Prov2multiplateADP, na.rm=TRUE)
[1] 77.39394
> sd(epi$Prov2multiplateADP, na.rm=TRUE)
[1] 27.00456
>
> mean(epi$Prov1multiplateCOL, na.rm=TRUE)
[1] 62.26316
> sd(epi$Prov1multiplateCOL, na.rm=TRUE)
[1] 21.94918
> mean(epi$Prov2multiplateCOL, na.rm=TRUE)
[1] 94.30303
> sd(epi$Prov2multiplateCOL, na.rm=TRUE)
[1] 30.3526
>
> mean(epi$Prov1multiplateTRAP, na.rm=TRUE)
[1] 102.8684
> sd(epi$Prov1multiplateTRAP, na.rm=TRUE)
[1] 31.77979
> mean(epi$Prov2multiplateTRAP, na.rm=TRUE)
[1] 108.9697
> sd(epi$Prov2multiplateTRAP, na.rm=TRUE)
[1] 32.84194
>
> mean(epi$Prov1multiplateASPI, na.rm=TRUE)
[1] 72.10526
> sd(epi$Prov1multiplateASPI, na.rm=TRUE)
[1] 23.98174
> mean(epi$Prov2multiplateASPI, na.rm=TRUE)
[1] 94.09091
> sd(epi$Prov2multiplateASPI, na.rm=TRUE)
[1] 38.98186
>
> hist(epi$Prov1multiplateADP)
> hist(epi$Prov1multiplateCOL)
> hist(epi$Prov1multiplateTRAP)
```

```
> hist(epi$Prov1multiplateASPI)
>
> t.test(epi$Prov1multiplateTRAP,epi$Prov2multiplateTRAP,alternative =
c("two.sided"),paired = TRUE)

	Paired t-test

data:  epi$Prov1multiplateTRAP and epi$Prov2multiplateTRAP
t = -1.0839, df = 32, p-value = 0.2865
alternative hypothesis: true difference in means is not equal to 0
95 percent confidence interval:
 -24.430100   7.460403
sample estimates:
mean of the differences
              -8.484848

> wilcox.test(epi$Prov1multiplateTRAP,epi$Prov2multiplateTRAP,alternative =
c("two.sided"),paired = TRUE)

	Wilcoxon signed rank test with continuity correction

data:  epi$Prov1multiplateTRAP and epi$Prov2multiplateTRAP
V = 231.5, p-value = 0.3861
alternative hypothesis: true location shift is not equal to 0

Warning message:
In wilcox.test.default(epi$Prov1multiplateTRAP, epi$Prov2multiplateTRAP,  :
  cannot compute exact p-value with ties
>
> t.test(epi$Prov1multiplateASPI,epi$Prov2multiplateASPI,alternative =
c("two.sided"),paired = TRUE)

	Paired t-test

data:  epi$Prov1multiplateASPI and epi$Prov2multiplateASPI
t = -3.1759, df = 32, p-value = 0.003298
alternative hypothesis: true difference in means is not equal to 0
95 percent confidence interval:
 -37.801125  -8.259481
sample estimates:
mean of the differences
               -23.0303

> wilcox.test(epi$Prov1multiplateASPI,epi$Prov2multiplateASPI,alternative =
c("two.sided"),paired = TRUE)

	Wilcoxon signed rank test with continuity correction

data:  epi$Prov1multiplateASPI and epi$Prov2multiplateASPI
V = 124.5, p-value = 0.005454
alternative hypothesis: true location shift is not equal to 0

Warning message:
In wilcox.test.default(epi$Prov1multiplateASPI, epi$Prov2multiplateASPI,  :
  cannot compute exact p-value with ties
> x<-10
```

```
> y<-170
> arrows(x,y,x+1,y, length=0.05, angle=90, code=1)
> arrows(x,y,x+1,y, length=0.05, angle=90, code=2)
> text(x+0.5,y-6,"**",pos=3, cex=1.4)
>
> mean(epi$Prov1multiplateADP, na.rm=TRUE)
[1] 68.10526
> sd(epi$Prov1multiplateADP, na.rm=TRUE)
[1] 21.04023
> mean(epi$Prov2multiplateADP, na.rm=TRUE)
[1] 77.39394
> sd(epi$Prov2multiplateADP, na.rm=TRUE)
[1] 27.00456
>
> mean(epi$Prov1multiplateCOL, na.rm=TRUE)
[1] 62.26316
> sd(epi$Prov1multiplateCOL, na.rm=TRUE)
[1] 21.94918
> mean(epi$Prov2multiplateCOL, na.rm=TRUE)
[1] 94.30303
> sd(epi$Prov2multiplateCOL, na.rm=TRUE)
[1] 30.3526
>
> mean(epi$Prov1multiplateTRAP, na.rm=TRUE)
[1] 102.8684
> sd(epi$Prov1multiplateTRAP, na.rm=TRUE)
[1] 31.77979
> mean(epi$Prov2multiplateTRAP, na.rm=TRUE)
[1] 108.9697
> sd(epi$Prov2multiplateTRAP, na.rm=TRUE)
[1] 32.84194
>
> mean(epi$Prov1multiplateASPI, na.rm=TRUE)
[1] 72.10526
> sd(epi$Prov1multiplateASPI, na.rm=TRUE)
[1] 23.98174
> mean(epi$Prov2multiplateASPI, na.rm=TRUE)
[1] 94.09091
> sd(epi$Prov2multiplateASPI, na.rm=TRUE)
[1] 38.98186
>
> hist(epi$Prov1multiplateADP)
> hist(epi$Prov1multiplateCOL)
> hist(epi$Prov1multiplateTRAP)
> hist(epi$Prov1multiplateASPI)
>
>
>
>
> #rutinPK
> dev.new(width=3)
> boxplot(epi$Prov1rutinPK,epi$Prov2rutinPK, horizontal=FALSE, na.rm=TRUE,
axes=TRUE, main="PT-INR",cex.main=1.65,
ylim=c(0.8,1.8),cex.axis=1.5,cex.lab=1.45, xlim=c(0,2.5),ylab="PT-INR",
boxwex=1, col=c(24,0), lty=1)
> #legend(0.1,1.8, c("Preoperative","Postoperative"), cex= 1,fill=c(24,0))
```

```
> arrows(0.25,0.9,,1.2,code=0,lwd=2,lty=3)
>
> t.test(epi$Prov1rutinPK,epi$Prov2rutinPK,alternative = c("two.sided"),paired
= TRUE)

    Paired t-test

data:  epi$Prov1rutinPK and epi$Prov2rutinPK
t = -5.6176, df = 30, p-value = 4.077e-06
alternative hypothesis: true difference in means is not equal to 0
95 percent confidence interval:
 -0.20673186 -0.09649394
sample estimates:
mean of the differences
             -0.1516129

> wilcox.test(epi$Prov1rutinPK,epi$Prov2rutinPK,alternative =
c("two.sided"),paired = TRUE)

    Wilcoxon signed rank test with continuity correction

data:  epi$Prov1rutinPK and epi$Prov2rutinPK
V = 0, p-value = 3.92e-05
alternative hypothesis: true location shift is not equal to 0

Warning messages:
1: In wilcox.test.default(epi$Prov1rutinPK, epi$Prov2rutinPK, alternative =
c("two.sided"),  :
  cannot compute exact p-value with ties
2: In wilcox.test.default(epi$Prov1rutinPK, epi$Prov2rutinPK, alternative =
c("two.sided"),  :
  cannot compute exact p-value with zeroes
> x<-1
> y<-0.83
> arrows(x,y,x+1,y, length=0.05, angle=90, code=1)
> arrows(x,y,x+1,y, length=0.05, angle=90, code=2)
> text(x+0.5,0.8,"**",pos=3, cex=1.4)
>
>
>
> #rutinAPTT
> dev.new(width=3)
> boxplot(epi$Prov1rutinAPTT,epi$Prov2rutinAPTT, horizontal=FALSE, na.rm=TRUE,
axes=TRUE, main="aPTT",cex.main=1.65, ylim=c(19,max(epi$Prov1rutinAPTT,epi
$Prov2rutinAPTT,na.rm=TRUE)),cex.axis=1.5,cex.lab=1.45,
xlim=c(0,2.5),ylab="aPTT (s)", boxwex=1, col=c(24,0), lty=1)
> arrows(0.25,26,,33,code=0,lwd=2,lty=3)
>
> t.test(epi$Prov1rutinAPTT,epi$Prov2rutinAPTT,alternative =
c("two.sided"),paired = TRUE)

    Paired t-test

data:  epi$Prov1rutinAPTT and epi$Prov2rutinAPTT
t = -2.8253, df = 29, p-value = 0.008461
alternative hypothesis: true difference in means is not equal to 0
```

```
95 percent confidence interval:
 -4.3671924 -0.6994742
sample estimates:
mean of the differences
              -2.533333

> wilcox.test(epi$Prov1rutinAPTT,epi$Prov2rutinAPTT,alternative =
c("two.sided"),paired = TRUE)

    Wilcoxon signed rank test with continuity correction

data:  epi$Prov1rutinAPTT and epi$Prov2rutinAPTT
V = 89.5, p-value = 0.005653
alternative hypothesis: true location shift is not equal to 0

Warning messages:
1: In wilcox.test.default(epi$Prov1rutinAPTT, epi$Prov2rutinAPTT, alternative =
c("two.sided"),  :
  cannot compute exact p-value with ties
2: In wilcox.test.default(epi$Prov1rutinAPTT, epi$Prov2rutinAPTT, alternative =
c("two.sided"),  :
  cannot compute exact p-value with zeroes
> x<-1
> y<-20.5
> arrows(x,y,x+1,y, length=0.05, angle=90, code=1)
> arrows(x,y,x+1,y, length=0.05, angle=90, code=2)
> text(x+0.5,y-0.7,"**",pos=3, cex=1.4)
>
> #rutinCRP
> dev.new(width=3)
> boxplot(epi$Prov1rutinCRP,epi$Prov2rutinCRP, horizontal=FALSE, na.rm=TRUE,
axes=TRUE, main="CRP",cex.main=1.65,cex.axis=1.5,cex.lab=1.45,
xlim=c(0,2.5),ylab="CRP (mg/L)", boxwex=1, col=c(24,0), lty=1)
> arrows(0.25,0,,3,code=0,lwd=2,lty=3)
> t.test(epi$Prov1rutinCRP,epi$Prov2rutinCRP,alternative =
c("two.sided"),paired = TRUE)

    Paired t-test

data:  epi$Prov1rutinCRP and epi$Prov2rutinCRP
t = -7.5742, df = 29, p-value = 2.38e-08
alternative hypothesis: true difference in means is not equal to 0
95 percent confidence interval:
 -107.688  -61.896
sample estimates:
mean of the differences
                -84.792

> wilcox.test(epi$Prov1rutinCRP,epi$Prov2rutinCRP,alternative =
c("two.sided"),paired = TRUE)

    Wilcoxon signed rank test

data:  epi$Prov1rutinCRP and epi$Prov2rutinCRP
V = 0, p-value = 1.863e-09
alternative hypothesis: true location shift is not equal to 0
```

```
> x<-1
> y<-250
> arrows(x,y,x+1,y, length=0.05, angle=90, code=1)
> arrows(x,y,x+1,y, length=0.05, angle=90, code=2)
> text(x+0.5,y-1,"**",pos=3, cex=1.4)
>
>
>
> #rutinTPK
> dev.new(width=3)
> boxplot(epi$Prov1rutinTPK,epi$Prov2rutinTPK, horizontal=FALSE, na.rm=TRUE,
axes=TRUE, main="Plc",cex.main=1.65, ylim=c(-50,max(epi$Prov1rutinTPK,epi
$Prov2rutinTPK,na.rm=TRUE)),cex.axis=1.5,cex.lab=1.45, xlim=c(0,2.5),ylab="Plc
(millions/mL)", boxwex=1, col=c(24,0), lty=1)
> arrows(0.25,145,,387,code=0,lwd=2,lty=3)
>
> t.test(epi$Prov1rutinTPK,epi$Prov2rutinTPK,alternative =
c("two.sided"),paired = TRUE)

	Paired t-test

data:  epi$Prov1rutinTPK and epi$Prov2rutinTPK
t = -3.0816, df = 30, p-value = 0.004385
alternative hypothesis: true difference in means is not equal to 0
95 percent confidence interval:
 -122.2372  -24.7951
sample estimates:
mean of the differences
              -73.51613

> wilcox.test(epi$Prov1rutinTPK,epi$Prov2rutinTPK,alternative =
c("two.sided"),paired = TRUE)

	Wilcoxon signed rank test with continuity correction

data:  epi$Prov1rutinTPK and epi$Prov2rutinTPK
V = 80.5, p-value = 0.001065
alternative hypothesis: true location shift is not equal to 0

Warning message:
In wilcox.test.default(epi$Prov1rutinTPK, epi$Prov2rutinTPK, alternative =
c("two.sided"),  :
  cannot compute exact p-value with ties
> x<-1
> y<--40
> arrows(x,y,x+1,y, length=0.05, angle=90, code=1)
> arrows(x,y,x+1,y, length=0.05, angle=90, code=2)
> text(x+0.5,y-30,"**",pos=3, cex=1.4)
>
> #Boxplot PIVKA
> hist(epi$PIVKAMpreop)
> hist(epi$PIVKAMpostop)
> dev.new(width=3)
> boxplot(epi$PIVKAMpreop,epi$PIVKAMpostop, horizontal=FALSE, na.rm=TRUE,
axes=TRUE, main="PIVKA",cex.main=1.65, ylim=c(0,89),cex.axis=1.5,cex.lab=1.45,
```

```
xlim=c(0,2.5),ylab="PIVKA (mg/L)", boxwex=1, col=c(24,0), lty=1)
> arrows(0.25,0,,0.2,code=0,lwd=2,lty=3)
>
> t.test(epi$PIVKAMpreop,epi$PIVKAMpostop,alternative = c("two.sided"),paired =
TRUE)

    Paired t-test

data:  epi$PIVKAMpreop and epi$PIVKAMpostop
t = -1.8204, df = 30, p-value = 0.07868
alternative hypothesis: true difference in means is not equal to 0
95 percent confidence interval:
 -49.134869   2.821966
sample estimates:
mean of the differences
              -23.15645

> wilcox.test(epi$PIVKAMpreop,epi$PIVKAMpostop,alternative =
c("two.sided"),paired = TRUE)

    Wilcoxon signed rank test

data:  epi$PIVKAMpreop and epi$PIVKAMpostop
V = 25, p-value = 8.419e-07
alternative hypothesis: true location shift is not equal to 0

> x<-1
> y<-70
> arrows(x,y,x+1,y, length=0.05, angle=90, code=1)
> arrows(x,y,x+1,y, length=0.05, angle=90, code=2)
> text(x+0.5,y-3,"**",pos=3, cex=1.4)
> legend(0.1,90, c("Preoperative","Postoperative"), cex= 1,fill=c(24,0))
>
> #Boxplot DDimer
> mean(epi$DDpreop, na.rm=TRUE)
[1] 0.2471081
> sd(epi$DDpreop, na.rm=TRUE)
[1] 0.224601
> mean(epi$DDpostop, na.rm=TRUE)
[1] 1.733645
> sd(epi$DDpostop, na.rm=TRUE)
[1] 1.04771
>
> hist(epi$DDpreop)
> hist(epi$DDpostop)
> dev.new(width=3)
> boxplot(epi$DDpreop,epi$DDpostop, horizontal=FALSE, na.rm=TRUE, axes=TRUE,
main="D-dimer",cex.main=1.65, ylim=c(0,6),cex.axis=1.5,cex.lab=1.45,
xlim=c(0,2.5),ylab="D-Dimer (mg/L)", boxwex=1, col=c(24,0), lty=1,plot=TRUE)
> arrows(0.25,0,,0.25,code=0,lwd=2,lty=3)
>
> t.test(epi$DDpreop,epi$DDpostop,alternative = c("two.sided"),paired = TRUE)

    Paired t-test

data:  epi$DDpreop and epi$DDpostop
```

```
t = -8.1538, df = 30, p-value = 4.213e-09
alternative hypothesis: true difference in means is not equal to 0
95 percent confidence interval:
 -1.841091 -1.103554
sample estimates:
mean of the differences
              -1.472323

> wilcox.test(epi$DDpreop,epi$DDpostop,alternative = c("two.sided"),paired =
TRUE)

    Wilcoxon signed rank test

data:  epi$DDpreop and epi$DDpostop
V = 0, p-value = 9.313e-10
alternative hypothesis: true location shift is not equal to 0

> x<-1
> y<-5
> arrows(x,y,x+1,y, length=0.05, angle=90, code=1)
> arrows(x,y,x+1,y, length=0.05, angle=90, code=2)
> text(x+0.5,y-0.2,"**",pos=3, cex=1.4)
> #legend(0.1,90, c("Preoperative","Postoperative"), cex= 1,fill=c(24,0))
>
>
>
>
> ###Before and after diagrams
> #Before and after diagrams for EXTEM-MCF
> preop<-epi$Prov1extemMCF
> postop<-epi$Prov2extemMCF
> preopdata<-t(matrix(preop))
> postopdata<-t(matrix(postop))
> withdrawaltime<-t(matrix(epi$withdrawaltime))
> meanwithdrawaltime<-mean(epi$withdrawaltime, na.rm=TRUE)
> y<-rbind(preop, postop)
> beforeandafterdata<-rbind(withdrawaltime, preopdata, postopdata)
> beforeandafterdata
         [,1]      [,2]      [,3]      [,4] [,5]     [,6]      [,7]      [,8]
[1,] 11.13194  6.118056  8.145833  3.107639  3.1 10.07292  7.104167  6.100694
[2,] 56.00000 63.000000 73.000000 75.000000 66.0 67.00000 42.000000 63.000000
[3,] 71.00000 76.000000 74.000000 75.000000 77.0 73.00000 73.000000 73.000000
          [,9]     [,10]     [,11]    [,12]     [,13]     [,14] [,15]
[1,]  2.201389  5.145833  5.069444 14.09722  5.131944  6.180556    NA
[2,] 75.000000 61.000000 63.000000 57.00000 65.000000 69.000000    55
[3,] 75.000000 68.000000 77.000000 74.00000 66.000000 70.000000    NA
         [,16]     [,17]    [,18]     [,19] [,20]     [,21]     [,22]
[1,]  2.121528  5.194444 15.15278  5.909722    NA  5.111111  4.958333
[2,] 47.000000 59.000000 61.00000 54.000000    64 60.000000 64.000000
[3,] 63.000000 69.000000 80.00000 68.000000    NA 71.000000 64.000000
         [,23]     [,24]     [,25] [,26]     [,27]     [,28] [,29] [,30]
[1,]  5.145833  2.097222  2.180556    NA  5.059028  3.138889    NA    NA
[2,] 69.000000 58.000000 66.000000    74 79.000000 58.000000    67    74
[3,] 72.000000 65.000000 63.000000    NA 60.000000 54.000000    NA    NA
        [,31]     [,32]     [,33]     [,34]     [,35]     [,36]     [,37]
[1,] 11.17361  6.222222  6.180556  7.104167  6.291667  5.027778  7.076389
```

```
[2,] 59.00000 67.000000 61.000000 62.000000 71.000000 63.000000 72.000000
[3,] 80.00000 76.000000 77.000000 75.000000 77.000000 73.000000 79.000000
         [,38]
[1,]  6.097222
[2,] 75.000000
[3,] 77.000000
> ylimits<-c(0.9*(min(preop,postop,na.rm=TRUE)),max(preop,postop, na.rm=TRUE))
> xlimits<-c(0,max(epi$withdrawaltime, na.rm=TRUE))
> xlabel<-"Days after surgery"
> plot(c(0,beforeandafterdata[1,1]),c(beforeandafterdata[2,1],
beforeandafterdata[3,1]),
type="l",col="black",xlim=xlimits,ylim=ylimits,ann=TRUE,axes=TRUE,xlab=xlabel,
main="Individual pre- and postoperative ROTEM-EXTEM-MCF",ylab="Maximum clot
firmness (mm)",  cex.main=1.3, cex.lab=1.4,lwd=1)
> for (x in 2:38) {lines(c(0,beforeandafterdata[1,x]),
c(beforeandafterdata[2,x], beforeandafterdata[3,x]), type="l", col="black",
lwd=1)}
>
> #normal range - enter manually!
> lines(c(-.4,-.4), c(50,72), type="l", lwd=6, lty=3)
>
> #Average pre and postoperative values at t=0 and t=mean withdrawal time
> preopmean<-mean(epi$Prov1extemMCF, na.rm=TRUE)
> postopmean<-mean(epi$Prov2extemMCF, na.rm=TRUE)
> lines(c(0, meanwithdrawaltime), c(preopmean, postopmean), type="l", lwd=6,
lty=4)
>
> #Significance value for pre- to postop change - see t.test or Wilcoxon's.
> text(8,78,"P<0.01", cex=2)
>
>
>
> #Before and after diagrams for EXTEM-CT
> preop<-epi$Prov1extemCT
> postop<-epi$Prov2extemCT
> preopdata<-t(matrix(preop))
> postopdata<-t(matrix(postop))
> withdrawaltime<-t(matrix(epi$withdrawaltime))
> meanwithdrawaltime<-mean(epi$withdrawaltime, na.rm=TRUE)
> y<-rbind(preop, postop)
> beforeandafterdata<-rbind(withdrawaltime, preopdata, postopdata)
> beforeandafterdata
         [,1]      [,2]      [,3]      [,4] [,5]     [,6]       [,7]      [,8]
[1,] 11.13194  6.118056  8.145833  3.107639  3.1 10.07292   7.104167  6.100694
[2,] 46.00000 46.000000 44.000000 51.000000 47.0 41.00000 108.000000 25.000000
[3,] 23.00000 43.000000 40.000000 43.000000 37.0 40.00000  51.000000 42.000000
          [,9]     [,10]     [,11]    [,12]     [,13]     [,14] [,15]
[1,]  2.201389  5.145833  5.069444 14.09722  5.131944  6.180556    NA
[2,] 36.000000 33.000000 49.000000 32.00000 42.000000 44.000000    36
[3,] 38.000000 43.000000 31.000000 44.00000 42.000000 42.000000    NA
         [,16]     [,17]    [,18]     [,19] [,20]     [,21]     [,22]
[1,]  2.121528  5.194444 15.15278  5.909722    NA  5.111111  4.958333
[2,] 45.000000 38.000000 41.00000 44.000000    44 42.000000 38.000000
[3,] 57.000000 42.000000 47.00000 41.000000    NA 41.000000 48.000000
         [,23]     [,24]     [,25] [,26]     [,27]     [,28] [,29] [,30]
[1,]  5.145833  2.097222  2.180556    NA  5.059028  3.138889    NA    NA
```

```
[2,] 52.000000 52.000000 43.000000    70 57.000000 54.000000    68    63
[3,] 59.000000 44.000000 51.000000    NA 61.000000 63.000000    NA    NA
        [,31]     [,32]     [,33]     [,34]     [,35]     [,36]     [,37]
[1,] 11.17361  6.222222  6.180556  7.104167  6.291667  5.027778  7.076389
[2,] 40.00000 39.000000 47.000000 42.000000 41.000000 47.000000 56.000000
[3,] 46.00000 53.000000 44.000000 48.000000 80.000000 55.000000 72.000000
         [,38]
[1,]  6.097222
[2,] 63.000000
[3,] 77.000000
> ylimits<-c(0.9*(min(preop,postop,na.rm=TRUE)),max(preop,postop, na.rm=TRUE))
> xlimits<-c(0,max(epi$withdrawaltime, na.rm=TRUE))
> xlabel<-"Days after surgery"
> plot(c(0,beforeandafterdata[1,1]),c(beforeandafterdata[2,1],
beforeandafterdata[3,1]),
type="l",col="black",xlim=xlimits,ylim=ylimits,ann=TRUE,axes=TRUE,xlab=xlabel,
main="Individual pre- and postoperative ROTEM-EXTEM-CT",ylab="Clotting time
(s)",  cex.main=1.3, cex.lab=1.4,lwd=1)
> for (x in 2:38) {lines(c(0,beforeandafterdata[1,x]),
c(beforeandafterdata[2,x], beforeandafterdata[3,x]), type="l", col="black",
lwd=1)}
>
> #normal range - enter manually!
> lines(c(-.4,-.4), c(38,79), type="l", lwd=6, lty=3)
>
> #Average pre and postoperative values at t=0 and t=mean withdrawal time
> preopmean<-mean(epi$Prov1extemCT, na.rm=TRUE)
> postopmean<-mean(epi$Prov2extemCT, na.rm=TRUE)
> lines(c(0, meanwithdrawaltime), c(preopmean, postopmean), type="l", lwd=6,
lty=4)
>
> #Significance value for pre- to postop change - see t.test or Wilcoxon's.
> text(8,60,"N/S", cex=2)
>
>
> #Before and after diagrams for INTEM-MCF
> preop<-epi$Prov1intemMCF
> postop<-epi$Prov2intemMCF
> preopdata<-t(matrix(preop))
> postopdata<-t(matrix(postop))
> withdrawaltime<-t(matrix(epi$withdrawaltime))
> meanwithdrawaltime<-mean(epi$withdrawaltime, na.rm=TRUE)
> y<-rbind(preop, postop)
> beforeandafterdata<-rbind(withdrawaltime, preopdata, postopdata)
> beforeandafterdata
         [,1]      [,2]      [,3]      [,4] [,5]     [,6]      [,7]      [,8]
[1,] 11.13194  6.118056  8.145833  3.107639  3.1 10.07292  7.104167  6.100694
[2,] 60.00000 61.000000 75.000000 77.000000 62.0 65.00000 56.000000 61.000000
[3,] 74.00000 75.000000 79.000000 74.000000 69.0 77.00000 73.000000 73.000000
          [,9]     [,10]     [,11]    [,12]     [,13]     [,14] [,15]
[1,]  2.201389  5.145833  5.069444 14.09722  5.131944  6.180556    NA
[2,] 74.000000 61.000000 63.000000 59.00000 64.000000 71.000000    56
[3,] 73.000000 69.000000 73.000000 76.00000 64.000000 68.000000    NA
         [,16]     [,17]    [,18]     [,19] [,20]     [,21]     [,22]
[1,]  2.121528  5.194444 15.15278  5.909722    NA  5.111111  4.958333
[2,] 60.000000 57.000000 61.00000 54.000000    65 60.000000 63.000000
```

```
[3,] 61.000000 68.000000 77.00000 69.000000        NA 71.000000 71.000000
          [,23]     [,24]     [,25] [,26]     [,27]     [,28] [,29] [,30]
[1,]  5.145833  2.097222  2.180556    NA  5.059028  3.138889    NA    NA
[2,] 68.000000 59.000000 62.000000    70 58.000000 57.000000    64    69
[3,] 71.000000 64.000000 62.000000    NA 56.000000 48.000000    NA    NA
         [,31]     [,32]     [,33]     [,34]     [,35]     [,36]     [,37]
[1,] 11.17361  6.222222  6.180556  7.104167  6.291667  5.027778  7.076389
[2,] 58.00000 66.000000 62.000000 56.000000 70.000000 59.000000 71.000000
[3,] 76.00000 74.000000 73.000000 72.000000 77.000000 72.000000 77.000000
          [,38]
[1,]  6.097222
[2,] 71.000000
[3,] 39.000000
> ylimits<-c(0.9*(min(preop,postop,na.rm=TRUE)),max(preop,postop, na.rm=TRUE))
> xlimits<-c(0,max(epi$withdrawaltime, na.rm=TRUE))
> xlabel<-"Days after surgery"
> plot(c(0,beforeandafterdata[1,1]),c(beforeandafterdata[2,1],
beforeandafterdata[3,1]),
type="l",col="black",xlim=xlimits,ylim=ylimits,ann=TRUE,axes=TRUE,xlab=xlabel,
main="Individual pre- and postoperative ROTEM-INTEM-MCF",ylab="Maximum clot
firmness (mm)",  cex.main=1.3, cex.lab=1.4,lwd=1)
>
> for (x in 2:38) {lines(c(0,beforeandafterdata[1,x]),
c(beforeandafterdata[2,x], beforeandafterdata[3,x]), type="l", col="black",
lwd=1)}
>
> #normal range - enter manually!
> lines(c(-.4,-.4), c(50,72), type="l", lwd=6, lty=3)
>
> #Average pre and postoperative values at t=0 and t=mean withdrawal time
> preopmean<-mean(epi$Prov1intemMCF, na.rm=TRUE)
> postopmean<-mean(epi$Prov2intemMCF, na.rm=TRUE)
> lines(c(0, meanwithdrawaltime), c(preopmean, postopmean), type="l", lwd=6,
lty=4)
>
> #Significance value for pre- to postop change - see t.test or Wilcoxon's.
> text(8,65,"P<0.01", cex=2)
>
>
>
> #Before and after diagrams for INTEM-CT
> preop<-epi$Prov1intemCT
> postop<-epi$Prov2intemCT
> preopdata<-t(matrix(preop))
> postopdata<-t(matrix(postop))
> withdrawaltime<-t(matrix(epi$withdrawaltime))
> meanwithdrawaltime<-mean(epi$withdrawaltime, na.rm=TRUE)
> y<-rbind(preop, postop)
> beforeandafterdata<-rbind(withdrawaltime, preopdata, postopdata)
> beforeandafterdata
          [,1]       [,2]       [,3]       [,4]  [,5]      [,6]       [,7]
[1,]  11.13194   6.118056   8.145833   3.107639   3.1  10.07292   7.104167
[2,] 161.00000 290.000000 171.000000 145.000000 357.0 211.00000 188.000000
[3,] 178.00000 159.000000 153.000000 142.000000 173.0 147.00000 110.000000
           [,8]       [,9]      [,10]      [,11]     [,12]      [,13]
[1,]   6.100694   2.201389   5.145833   5.069444  14.09722   5.131944
```

```
[2,] 175.000000 157.000000 177.000000 161.000000 137.00000 152.000000
[3,] 134.000000 152.000000 131.000000 164.000000 205.00000 171.000000
          [,14] [,15]      [,16]      [,17]     [,18]      [,19] [,20]
[1,]   6.180556    NA   2.121528   5.194444  15.15278   5.909722    NA
[2,] 195.000000   144 227.000000 202.000000 193.00000 229.000000   141
[3,] 227.000000    NA 156.000000 161.000000 212.00000 182.000000    NA
          [,21]      [,22]      [,23]      [,24]      [,25] [,26]      [,27]
[1,]   5.111111   4.958333   5.145833   2.097222   2.180556    NA   5.059028
[2,] 134.000000 147.000000 121.000000 199.000000 198.000000   170 158.000000
[3,] 200.000000 122.000000 167.000000 132.000000 223.000000    NA 168.000000
          [,28] [,29] [,30]     [,31]      [,32]      [,33]      [,34]
[1,]   3.138889    NA    NA  11.17361   6.222222   6.180556   7.104167
[2,] 159.000000   125   150 184.00000 181.000000 177.000000 133.000000
[3,] 147.000000    NA    NA 147.00000 150.000000 337.000000 148.000000
          [,35]      [,36]      [,37]      [,38]
[1,]   6.291667   5.027778   7.076389   6.097222
[2,] 204.000000 185.000000 148.000000 100.000000
[3,] 165.000000 133.000000 156.000000  94.000000
> ylimits<-c(0.9*(min(preop,postop,na.rm=TRUE)),max(preop,postop, na.rm=TRUE))
> xlimits<-c(0,max(epi$withdrawaltime, na.rm=TRUE))
> xlabel<-"Days after surgery"
> plot(c(0,beforeandafterdata[1,1]),c(beforeandafterdata[2,1],
beforeandafterdata[3,1]),
type="l",col="black",xlim=xlimits,ylim=ylimits,ann=TRUE,axes=TRUE,xlab=xlabel,
main="Individual pre- and postoperative ROTEM-INTEM-CT",ylab="Clotting time
(s)",  cex.main=1.3, cex.lab=1.4,lwd=1)
> for (x in 2:38) {lines(c(0,beforeandafterdata[1,x]),
c(beforeandafterdata[2,x], beforeandafterdata[3,x]), type="l", col="black",
lwd=1)}
>
> #normal range - enter manually!
> lines(c(-.4,-.4), c(100,240), type="l", lwd=6, lty=3)
>
> #Average pre and postoperative values at t=0 and t=mean withdrawal time
> preopmean<-mean(epi$Prov1intemCT, na.rm=TRUE)
> postopmean<-mean(epi$Prov2intemCT, na.rm=TRUE)
> lines(c(0, meanwithdrawaltime), c(preopmean, postopmean), type="l", lwd=6,
lty=4)
>
> #Significance value for pre- to postop change - see t.test or Wilcoxon's.
> text(8,220,"N/S", cex=2)
>
>
>
> #Before and after diagrams for HEPTEM-MCF
> preop<-epi$Prov1heptemMCF
> postop<-epi$Prov2heptemMCF
> preopdata<-t(matrix(preop))
> postopdata<-t(matrix(postop))
> withdrawaltime<-t(matrix(epi$withdrawaltime))
> meanwithdrawaltime<-mean(epi$withdrawaltime, na.rm=TRUE)
> y<-rbind(preop, postop)
> beforeandafterdata<-rbind(withdrawaltime, preopdata, postopdata)
> beforeandafterdata
         [,1]      [,2]      [,3]      [,4] [,5]     [,6]      [,7]      [,8]
[1,] 11.13194  6.118056  8.145833  3.107639  3.1 10.07292  7.104167  6.100694
```

```
[2,] 60.00000 59.000000 72.000000 75.000000 64.0 63.00000 56.000000 62.000000
[3,] 71.00000 69.000000 75.000000 75.000000 70.0 75.00000 70.000000 70.000000
          [,9]     [,10]     [,11]    [,12]     [,13]     [,14] [,15]
[1,]  2.201389  5.145833  5.069444 14.09722  5.131944  6.180556    NA
[2,] 73.000000 57.000000 59.000000 61.00000 63.000000 68.000000    53
[3,] 75.000000        NA 73.000000 74.00000 63.000000 67.000000    NA
         [,16]     [,17]    [,18]     [,19] [,20]     [,21]     [,22]
[1,]  2.121528  5.194444 15.15278  5.909722    NA  5.111111  4.958333
[2,] 61.000000 54.000000 59.00000 55.000000    61 60.000000 63.000000
[3,] 57.000000 66.000000 76.00000 67.000000    NA 71.000000 70.000000
         [,23]     [,24]     [,25] [,26]     [,27]     [,28] [,29] [,30]
[1,]  5.145833  2.097222  2.180556    NA  5.059028  3.138889    NA    NA
[2,] 68.000000 58.000000 62.000000    70 59.000000 55.000000    60    68
[3,] 71.000000 63.000000 62.000000    NA 58.000000 48.000000    NA    NA
        [,31]     [,32]     [,33]     [,34]     [,35]     [,36]     [,37]
[1,] 11.17361  6.222222  6.180556  7.104167  6.291667  5.027778  7.076389
[2,] 58.00000 65.000000 63.000000 52.000000 69.000000 58.000000 69.000000
[3,] 71.00000 72.000000 74.000000 70.000000 76.000000 71.000000 77.000000
         [,38]
[1,]  6.097222
[2,] 71.000000
[3,] 74.000000
> ylimits<-c(0.9*(min(preop,postop,na.rm=TRUE)),max(preop,postop, na.rm=TRUE))
> xlimits<-c(0,max(epi$withdrawaltime, na.rm=TRUE))
> xlabel<-"Days after surgery"
> plot(c(0,beforeandafterdata[1,1]),c(beforeandafterdata[2,1],
beforeandafterdata[3,1]),
type="l",col="black",xlim=xlimits,ylim=ylimits,ann=TRUE,axes=TRUE,xlab=xlabel,
main="Individual pre- and postoperative ROTEM-HEPTEM-MCF",ylab="Maximum clot
firmness (mm)",  cex.main=1.3, cex.lab=1.4,lwd=1)
>
> for (x in 2:38) {lines(c(0,beforeandafterdata[1,x]),
c(beforeandafterdata[2,x], beforeandafterdata[3,x]), type="l", col="black",
lwd=1)}
>
> #normal range - enter manually!
> lines(c(-.4,-.4), c(50,72), type="l", lwd=6, lty=3)
>
> #Average pre and postoperative values at t=0 and t=mean withdrawal time
> preopmean<-mean(epi$Prov1heptemMCF, na.rm=TRUE)
> postopmean<-mean(epi$Prov2heptemMCF, na.rm=TRUE)
> lines(c(0, meanwithdrawaltime), c(preopmean, postopmean), type="l", lwd=6,
lty=4)
>
> #Significance value for pre- to postop change - see t.test or Wilcoxon's.
> text(8,64,"P<0.01", cex=2)
>
>
>
> #Before and after diagrams for HEPTEM-CT
> preop<-epi$Prov1heptemCT
> postop<-epi$Prov2heptemCT
> preopdata<-t(matrix(preop))
> postopdata<-t(matrix(postop))
> withdrawaltime<-t(matrix(epi$withdrawaltime))
> meanwithdrawaltime<-mean(epi$withdrawaltime, na.rm=TRUE)
```

```
> y<-rbind(preop, postop)
> beforeandafterdata<-rbind(withdrawaltime, preopdata, postopdata)
> beforeandafterdata
         [,1]       [,2]       [,3]       [,4]  [,5]      [,6]       [,7]
[1,]  11.13194   6.118056   8.145833   3.107639   3.1  10.07292   7.104167
[2,] 188.00000 357.000000 223.000000 225.000000 139.0 252.00000 197.000000
[3,] 184.00000 166.000000 162.000000 151.000000 211.0 169.00000 127.000000
           [,8]       [,9]      [,10]      [,11]     [,12]      [,13]
[1,]   6.100694   2.201389   5.145833   5.069444  14.09722   5.131944
[2,] 199.000000 174.000000 178.000000 220.000000 169.00000 185.000000
[3,] 145.000000 133.000000  61.000000 207.000000 241.00000 205.000000
          [,14] [,15]      [,16]      [,17]     [,18]      [,19] [,20]
[1,]   6.180556    NA   2.121528   5.194444  15.15278   5.909722    NA
[2,] 125.000000   177 132.000000 287.000000 298.00000 135.000000   190
[3,] 261.000000    NA 184.000000 223.000000 212.00000 228.000000    NA
          [,21]      [,22]      [,23]      [,24]      [,25] [,26]      [,27]
[1,]   5.111111   4.958333   5.145833   2.097222   2.180556    NA   5.059028
[2,] 183.000000 153.000000 182.000000 150.000000 219.000000   155 157.000000
[3,] 229.000000 130.000000 216.000000 163.000000 250.000000    NA 159.000000
          [,28] [,29] [,30]     [,31]      [,32]      [,33]      [,34]
[1,]   3.138889    NA    NA  11.17361   6.222222   6.180556   7.104167
[2,] 199.000000   143   180 183.00000 162.000000 100.000000 182.000000
[3,] 160.000000    NA    NA 147.00000 199.000000 278.000000 158.000000
          [,35]      [,36]      [,37]      [,38]
[1,]   6.291667   5.027778   7.076389   6.097222
[2,] 219.000000 205.000000 159.000000 154.000000
[3,] 165.000000 146.000000 154.000000 173.000000
> ylimits<-c(0.9*(min(preop,postop,na.rm=TRUE)),max(preop,postop, na.rm=TRUE))
> xlimits<-c(0,max(epi$withdrawaltime, na.rm=TRUE))
> xlabel<-"Days after surgery"
> plot(c(0,beforeandafterdata[1,1]),c(beforeandafterdata[2,1],
beforeandafterdata[3,1]),
type="l",col="black",xlim=xlimits,ylim=ylimits,ann=TRUE,axes=TRUE,xlab=xlabel,
main="Individual pre- and postoperative ROTEM-HEPTEM-CT",ylab="Clotting time
(s)",  cex.main=1.3, cex.lab=1.4,lwd=1)
> for (x in 2:38) {lines(c(0,beforeandafterdata[1,x]),
c(beforeandafterdata[2,x], beforeandafterdata[3,x]), type="l", col="black",
lwd=1)}
>
> #normal range - enter manually!
> lines(c(-.4,-.4), c(100,240), type="l", lwd=6, lty=3)
>
> #Average pre and postoperative values at t=0 and t=mean withdrawal time
> preopmean<-mean(epi$Prov1heptemCT, na.rm=TRUE)
> postopmean<-mean(epi$Prov2heptemCT, na.rm=TRUE)
> lines(c(0, meanwithdrawaltime), c(preopmean, postopmean), type="l", lwd=6,
lty=4)
>
> #Significance value for pre- to postop change - see t.test or Wilcoxon's.
> text(7,220,"N/S", cex=2)
>
>
> #Before and after diagrams for FIBTEM-MCF
> preop<-epi$Prov1fibtemMCF
> postop<-epi$Prov2fibtemMCF
> preopdata<-t(matrix(preop))
```

```
> postopdata<-t(matrix(postop))
> withdrawaltime<-t(matrix(epi$withdrawaltime))
> meanwithdrawaltime<-mean(epi$withdrawaltime, na.rm=TRUE)
> y<-rbind(preop, postop)
> beforeandafterdata<-rbind(withdrawaltime, preopdata, postopdata)
> beforeandafterdata
         [,1]      [,2]      [,3]      [,4] [,5]     [,6]      [,7]      [,8]
[1,] 11.13194  6.118056  8.145833  3.107639  3.1 10.07292  7.104167  6.100694
[2,] 11.00000 20.000000 35.000000 26.000000 22.0 19.00000 12.000000 15.000000
[3,] 43.00000 37.000000 40.000000 39.000000 37.0 40.00000 52.000000 34.000000
          [,9]     [,10]     [,11]    [,12]     [,13]     [,14] [,15]
[1,]  2.201389  5.145833  5.069444 14.09722  5.131944  6.180556    NA
[2,] 34.000000 25.000000 21.000000 13.00000 22.000000 26.000000    17
[3,] 35.000000 31.000000 41.000000 40.00000 31.000000 24.000000    NA
         [,16]     [,17]    [,18]     [,19] [,20]     [,21]     [,22]
[1,]  2.121528  5.194444 15.15278  5.909722    NA  5.111111  4.958333
[2,] 13.000000 13.000000 17.00000 11.000000    19 19.000000 13.000000
[3,] 15.000000 25.000000 48.00000 31.000000    NA 29.000000 26.000000
         [,23]     [,24]     [,25] [,26]     [,27]     [,28] [,29] [,30]
[1,]  5.145833  2.097222  2.180556    NA  5.059028  3.138889    NA    NA
[2,] 28.000000 11.000000 16.000000    29 30.000000 13.000000    17    26
[3,] 31.000000 22.000000 24.000000    NA 16.000000 20.000000    NA    NA
        [,31]     [,32]     [,33]     [,34]     [,35]     [,36]     [,37]
[1,] 11.17361  6.222222  6.180556  7.104167  6.291667  5.027778  7.076389
[2,] 16.00000        NA 18.000000 13.000000 32.000000 20.000000 17.000000
[3,] 49.00000 39.000000 42.000000 27.000000 38.000000 31.000000 34.000000
         [,38]
[1,]  6.097222
[2,] 34.000000
[3,] 42.000000
> ylimits<-c(0.9*(min(preop,postop,na.rm=TRUE)),max(preop,postop, na.rm=TRUE))
> xlimits<-c(0,max(epi$withdrawaltime, na.rm=TRUE))
> xlabel<-"Days after surgery"
> plot(c(0,beforeandafterdata[1,1]),c(beforeandafterdata[2,1],
beforeandafterdata[3,1]),
type="l",col="black",xlim=xlimits,ylim=ylimits,ann=TRUE,axes=TRUE,xlab=xlabel,
main="Individual pre- and postoperative ROTEM-INTEM-MCF",ylab="Maximum clot
firmness (mm)",  cex.main=1.3, cex.lab=1.4,lwd=1)
>
> for (x in 2:38) {lines(c(0,beforeandafterdata[1,x]),
c(beforeandafterdata[2,x], beforeandafterdata[3,x]), type="l", col="black",
lwd=1)}
>
> #normal range - enter manually!
> lines(c(-.4,-.4), c(9,25), type="l", lwd=6, lty=3)
>
> #Average pre and postoperative values at t=0 and t=mean withdrawal time
> preopmean<-mean(epi$Prov1fibtemMCF, na.rm=TRUE)
> postopmean<-mean(epi$Prov2fibtemMCF, na.rm=TRUE)
> lines(c(0, meanwithdrawaltime), c(preopmean, postopmean), type="l", lwd=6,
lty=4)
>
> #Significance value for pre- to postop change - see t.test or Wilcoxon's.
> text(8,64,"P<0.01", cex=2)
>
>
```

```
> #Before and after diagrams for HEPTEM-INTEM-MCF
> preop<-epi$preopHEPTEMminusINTEMmcf
> postop<-epi$postopHEPTEMminusINTEMmcf
> preopdata<-t(matrix(preop))
> postopdata<-t(matrix(postop))
> withdrawaltime<-t(matrix(epi$withdrawaltime))
> meanwithdrawaltime<-mean(epi$withdrawaltime, na.rm=TRUE)
> y<-rbind(preop, postop)
> beforeandafterdata<-rbind(withdrawaltime, preopdata, postopdata)
> beforeandafterdata
         [,1]      [,2]      [,3]      [,4] [,5]     [,6]      [,7]      [,8]
[1,] 11.13194  6.118056  8.145833  3.107639  3.1 10.07292  7.104167  6.100694
[2,]  0.00000 -2.000000 -3.000000 -2.000000  2.0 -2.00000  0.000000  1.000000
[3,] -3.00000 -6.000000 -4.000000  1.000000  1.0 -2.00000 -3.000000 -3.000000
          [,9]     [,10]     [,11]    [,12]     [,13]     [,14] [,15]
[1,]  2.201389  5.145833  5.069444 14.09722  5.131944  6.180556    NA
[2,] -1.000000 -4.000000 -4.000000  2.00000 -1.000000 -3.000000    -3
[3,]  2.000000        NA  0.000000 -2.00000 -1.000000 -1.000000    NA
         [,16]     [,17]    [,18]     [,19] [,20]    [,21]     [,22]    [,23]
[1,]  2.121528  5.194444 15.15278  5.909722    NA 5.111111  4.958333 5.145833
[2,]  1.000000 -3.000000 -2.00000  1.000000    -4 0.000000  0.000000 0.000000
[3,] -4.000000 -2.000000 -1.00000 -2.000000    NA 0.000000 -1.000000 0.000000
         [,24]    [,25] [,26]    [,27]     [,28] [,29] [,30]    [,31]
[1,]  2.097222 2.180556    NA 5.059028  3.138889    NA    NA 11.17361
[2,] -1.000000 0.000000     0 1.000000 -2.000000    -4    -1  0.00000
[3,] -1.000000 0.000000    NA 2.000000  0.000000    NA    NA -5.00000
         [,32]    [,33]     [,34]     [,35]     [,36]     [,37]     [,38]
[1,]  6.222222 6.180556  7.104167  6.291667  5.027778  7.076389  6.097222
[2,] -1.000000 1.000000 -4.000000 -1.000000 -1.000000 -2.000000  0.000000
[3,] -2.000000 1.000000 -2.000000 -1.000000 -1.000000  0.000000 35.000000
> ylimits<-c(1.1*(min(preop,postop,na.rm=TRUE)),max(preop,postop, na.rm=TRUE))
> xlimits<-c(0,max(epi$withdrawaltime, na.rm=TRUE))
> xlabel<-"Days after surgery"
> plot(c(0,beforeandafterdata[1,1]),c(beforeandafterdata[2,1],
beforeandafterdata[3,1]),
type="l",col="black",xlim=xlimits,ylim=ylimits,ann=TRUE,axes=TRUE,xlab=xlabel,
main="Individual pre- and postoperative ROTEM-HEPTEM-INTEM-MCF",ylab="Maximum
clot firmness (mm)",  cex.main=1.3, cex.lab=1.4,lwd=1)
>
> for (x in 2:38) {lines(c(0,beforeandafterdata[1,x]),
c(beforeandafterdata[2,x], beforeandafterdata[3,x]), type="l", col="black",
lwd=1)}
>
> #normal range - enter manually!
> #there are no normal ranges for HEPTEM-INTEM. lines(c(-.4,-.4), c(50,72),
type="l", lwd=6, lty=3)
>
> #Average pre and postoperative values at t=0 and t=mean withdrawal time
> preopmean<-mean(epi$preopHEPTEMminusINTEMmcf, na.rm=TRUE)
> postopmean<-mean(epi$postopHEPTEMminusINTEMmcf, na.rm=TRUE)
> lines(c(0, meanwithdrawaltime), c(preopmean, postopmean), type="l", lwd=6,
lty=4)
>
> #Significance value for pre- to postop change - see t.test or Wilcoxon's.
> text(8,3,"N/S", cex=2)
>
```

```
>
>
> #Before and after diagrams for INTEM-HEPTEM-CT
> preop<-epi$preopINTEMminusHEPTEMct
> postop<-epi$postopINTEMminusHEPTEMct
> preopdata<-t(matrix(preop))
Error in matrix(preop) : 'data' must be of a vector type, was 'NULL'
> postopdata<-t(matrix(postop))
Error in matrix(postop) : 'data' must be of a vector type, was 'NULL'
> withdrawaltime<-t(matrix(epi$withdrawaltime))
> meanwithdrawaltime<-mean(epi$withdrawaltime, na.rm=TRUE)
> y<-rbind(preop, postop)
> beforeandafterdata<-rbind(withdrawaltime, preopdata, postopdata)
> ylimits<-c(1.1*(min(preop,postop,na.rm=TRUE)),max(preop,postop, na.rm=TRUE))
Warning messages:
1: In min(preop, postop, na.rm = TRUE) :
  no non-missing arguments to min; returning Inf
2: In max(preop, postop, na.rm = TRUE) :
  no non-missing arguments to max; returning -Inf
> xlimits<-c(0,max(epi$withdrawaltime, na.rm=TRUE))
> xlabel<-"Days after surgery"
> plot(c(0,beforeandafterdata[1,1]),c(beforeandafterdata[2,1],
beforeandafterdata[3,1]),
type="l",col="black",xlim=xlimits,ylim=ylimits,ann=TRUE,axes=TRUE,xlab=xlabel,
main="Individual pre- and postoperative ROTEM-INTEM-HEPTEM-CT",ylab="Clotting
time (s)",  cex.main=1.3, cex.lab=1.4,lwd=1)
Error in plot.window(...) : need finite 'ylim' values
> for (x in 2:38) {lines(c(0,beforeandafterdata[1,x]),
c(beforeandafterdata[2,x], beforeandafterdata[3,x]), type="l", col="black",
lwd=1)}
>
> #normal range - enter manually!
> ## there are no normal values here. lines(c(-.4,-.4), c(100,240), type="l",
lwd=6, lty=3)
>
> #Average pre and postoperative values at t=0 and t=mean withdrawal time
> preopmean<-mean(epi$preopINTEMminusHEPTEMct, na.rm=TRUE)
Warning message:
In mean.default(epi$preopINTEMminusHEPTEMct, na.rm = TRUE) :
  argument is not numeric or logical: returning NA
> postopmean<-mean(epi$postopINTEMminusHEPTEMct, na.rm=TRUE)
Warning message:
In mean.default(epi$postopINTEMminusHEPTEMct, na.rm = TRUE) :
  argument is not numeric or logical: returning NA
> lines(c(0, meanwithdrawaltime), c(preopmean, postopmean), type="l", lwd=6,
lty=4)
>
> #Significance value for pre- to postop change - see t.test or Wilcoxon's.
> text(7,13,"N/S", cex=2)
>
>
>
>
>
> ##Multiplate before and after diagrams
> #Before and after diagrams for multiplateADP
```

```
> preop<-epi$Prov1multiplateADP
> postop<-epi$Prov2multiplateADP
> preopdata<-t(matrix(preop))
> postopdata<-t(matrix(postop))
> withdrawaltime<-t(matrix(epi$withdrawaltime))
> meanwithdrawaltime<-mean(epi$withdrawaltime, na.rm=TRUE)
> y<-rbind(preop, postop)
> beforeandafterdata<-rbind(withdrawaltime, preopdata, postopdata)
> beforeandafterdata
          [,1]      [,2]      [,3]      [,4] [,5]     [,6]       [,7]
[1,]  11.13194  6.118056  8.145833  3.107639  3.1 10.07292   7.104167
[2,]  63.00000 63.000000 63.000000 81.000000 89.0 67.00000  13.000000
[3,] 114.00000 84.000000 16.000000 94.000000 57.0 61.00000 134.000000
          [,8]      [,9]     [,10]     [,11]    [,12]     [,13]     [,14]
[1,]  6.100694  2.201389  5.145833  5.069444 14.09722  5.131944  6.180556
[2,] 79.000000 76.000000 48.000000 94.000000 26.00000 41.000000 82.000000
[3,] 94.000000 57.000000 65.000000 66.000000 85.00000 59.000000 82.000000
     [,15]     [,16]     [,17]     [,18]     [,19] [,20]     [,21]     [,22]
[1,]    NA  2.121528  5.194444  15.15278  5.909722    NA  5.111111  4.958333
[2,]    46 60.000000 54.000000  67.00000 72.000000    62 62.000000 86.000000
[3,]    NA 47.000000 64.000000 143.00000 98.000000    NA 95.000000 72.000000
         [,23]     [,24]     [,25] [,26]     [,27]     [,28] [,29] [,30]
[1,]  5.145833  2.097222  2.180556    NA  5.059028  3.138889    NA    NA
[2,] 69.000000 95.000000 89.000000    82 77.000000 60.000000    81    88
[3,] 72.000000 50.000000 54.000000    NA 59.000000 29.000000    NA    NA
         [,31]     [,32]      [,33]     [,34]     [,35]     [,36]     [,37]
[1,]  11.17361  6.222222   6.180556  7.104167  6.291667  5.027778  7.076389
[2,]  38.00000 86.000000  95.000000 51.000000 86.000000 29.000000 63.000000
[3,] 102.00000 87.000000 115.000000 86.000000 78.000000 69.000000 91.000000
          [,38]
[1,]   6.097222
[2,] 105.000000
[3,]  75.000000
> ylimits<-c(0.9*(min(preop,postop,na.rm=TRUE)),max(preop,postop, na.rm=TRUE))
> xlimits<-c(0,max(epi$withdrawaltime, na.rm=TRUE))
> xlabel<-"Days after surgery"
> plot(c(0,beforeandafterdata[1,1]),c(beforeandafterdata[2,1],
beforeandafterdata[3,1]),
type="l",col="black",xlim=xlimits,ylim=ylimits,ann=TRUE,axes=TRUE,xlab=xlabel,
main="Individual pre- and postoperative Multiplate ADP",ylab="Area under curve
(AUC)",  cex.main=1.3, cex.lab=1.4,lwd=1)
>
> for (x in 2:38) {lines(c(0,beforeandafterdata[1,x]),
c(beforeandafterdata[2,x], beforeandafterdata[3,x]), type="l", col="black",
lwd=1)}
>
> #normal range - enter manually!
> lines(c(-.4,-.4), c(57,113), type="l", lwd=6, lty=3)
>
> #Average pre and postoperative values at t=0 and t=mean withdrawal time
> preopmean<-mean(epi$Prov1multiplateADP, na.rm=TRUE)
> postopmean<-mean(epi$Prov2multiplateADP, na.rm=TRUE)
> lines(c(0, meanwithdrawaltime), c(preopmean, postopmean), type="l", lwd=6,
lty=4)
>
> #Significance value for pre- to postop change - see t.test or Wilcoxon's.
```

```
> text(8,72,"N/S", cex=2)
>
>
>
> #Before and after diagrams for multiplateCOL
> preop<-epi$Prov1multiplateCOL
> postop<-epi$Prov2multiplateCOL
> preopdata<-t(matrix(preop))
> postopdata<-t(matrix(postop))
> withdrawaltime<-t(matrix(epi$withdrawaltime))
> meanwithdrawaltime<-mean(epi$withdrawaltime, na.rm=TRUE)
> y<-rbind(preop, postop)
> beforeandafterdata<-rbind(withdrawaltime, preopdata, postopdata)
> beforeandafterdata
          [,1]      [,2]      [,3]       [,4] [,5]      [,6]       [,7]
[1,]  11.13194  6.118056  8.145833   3.107639  3.1  10.07292   7.104167
[2,]  66.00000 64.000000 40.000000 100.000000 66.0  67.00000  11.000000
[3,] 131.00000 96.000000 81.000000 132.000000 76.0 153.00000 152.000000
           [,8]       [,9]     [,10]     [,11]     [,12]     [,13]     [,14]
[1,]   6.100694   2.201389  5.145833  5.069444  14.09722  5.131944  6.180556
[2,]  94.000000 100.000000 60.000000 70.000000  42.00000 45.000000 72.000000
[3,] 101.000000 113.000000 71.000000 70.000000 118.00000 64.000000 76.000000
     [,15]     [,16]     [,17]     [,18]     [,19] [,20]     [,21]     [,22]
[1,]    NA  2.121528  5.194444  15.15278  5.909722    NA  5.111111  4.958333
[2,]    40 61.000000 25.000000  41.00000 56.000000    64 54.000000 75.000000
[3,]    NA 53.000000 64.000000 159.00000 93.000000    NA 98.000000 73.000000
         [,23]     [,24]     [,25] [,26]     [,27]     [,28] [,29] [,30]
[1,]  5.145833  2.097222  2.180556    NA  5.059028  3.138889    NA    NA
[2,] 42.000000 76.000000 69.000000    86 57.000000 46.000000    67    86
[3,] 74.000000 75.000000 75.000000    NA 52.000000 35.000000    NA    NA
         [,31]      [,32]     [,33]      [,34]      [,35]     [,36]      [,37]
[1,]  11.17361   6.222222  6.180556   7.104167   6.291667  5.027778   7.076389
[2,]  46.00000  52.000000 88.000000  35.000000  98.000000 38.000000  65.000000
[3,] 106.00000 108.000000 98.000000 104.000000 129.000000 79.000000 115.000000
          [,38]
[1,]   6.097222
[2,] 102.000000
[3,]  88.000000
> ylimits<-c(0.9*(min(preop,postop,na.rm=TRUE)),max(preop,postop, na.rm=TRUE))
> xlimits<-c(0,max(epi$withdrawaltime, na.rm=TRUE))
> xlabel<-"Days after surgery"
> plot(c(0,beforeandafterdata[1,1]),c(beforeandafterdata[2,1],
beforeandafterdata[3,1]),
type="l",col="black",xlim=xlimits,ylim=ylimits,ann=TRUE,axes=TRUE,xlab=xlabel,
main="Individual pre- and postoperative Multiplate COL",ylab="Area under curve
(AUC)",  cex.main=1.3, cex.lab=1.4,lwd=1)
>
> for (x in 2:38) {lines(c(0,beforeandafterdata[1,x]),
c(beforeandafterdata[2,x], beforeandafterdata[3,x]), type="l", col="black",
lwd=1)}
>
> #normal range - enter manually!
> lines(c(-.4,-.4), c(72,125), type="l", lwd=6, lty=3)
>
> #Average pre and postoperative values at t=0 and t=mean withdrawal time
> preopmean<-mean(epi$Prov1multiplateCOL, na.rm=TRUE)
```

```
> postopmean<-mean(epi$Prov2multiplateCOL, na.rm=TRUE)
> lines(c(0, meanwithdrawaltime), c(preopmean, postopmean), type="l", lwd=6,
lty=4)
>
> #Significance value for pre- to postop change - see t.test or Wilcoxon's.
> text(9,72,"P<0.01", cex=2)
>
>
> #Before and after diagrams for multiplateTRAP
> preop<-epi$Prov1multiplateTRAP
> postop<-epi$Prov2multiplateTRAP
> preopdata<-t(matrix(preop))
> postopdata<-t(matrix(postop))
> withdrawaltime<-t(matrix(epi$withdrawaltime))
> meanwithdrawaltime<-mean(epi$withdrawaltime, na.rm=TRUE)
> y<-rbind(preop, postop)
> beforeandafterdata<-rbind(withdrawaltime, preopdata, postopdata)
> beforeandafterdata
          [,1]       [,2]       [,3]       [,4]  [,5]      [,6]       [,7]
[1,]  11.13194   6.118056   8.145833   3.107639   3.1  10.07292   7.104167
[2,] 112.00000 115.000000 102.000000 129.000000 119.0 101.00000  22.000000
[3,] 150.00000 138.000000 135.000000 152.000000  97.0 153.00000 150.000000
           [,8]       [,9]     [,10]      [,11]     [,12]     [,13]      [,14]
[1,]   6.100694   2.201389  5.145833   5.069444  14.09722  5.131944   6.180556
[2,] 128.000000 126.000000 96.000000 124.000000  79.00000 80.000000  83.000000
[3,] 120.000000 102.000000 79.000000  93.000000 104.00000 89.000000 143.000000
     [,15]     [,16]     [,17]     [,18]      [,19] [,20]      [,21]
[1,]    NA  2.121528  5.194444  15.15278   5.909722    NA   5.111111
[2,]    75 92.000000 38.000000  79.00000  99.000000   101 113.000000
[3,]    NA 24.000000 82.000000 154.00000 114.000000    NA 108.000000
          [,22]     [,23]      [,24]      [,25] [,26]     [,27]      [,28]
[1,]   4.958333  5.145833   2.097222   2.180556    NA  5.059028   3.138889
[2,] 132.000000 71.000000 130.000000 105.000000   163 91.000000 113.000000
[3,] 103.000000 76.000000  71.000000  73.000000    NA 78.000000  41.000000
     [,29] [,30]     [,31]      [,32]      [,33]      [,34]      [,35]
[1,]    NA    NA  11.17361   6.222222   6.180556   7.104167   6.291667
[2,]   117   137  85.00000 138.000000 129.000000  62.000000 126.000000
[3,]    NA    NA 109.00000 145.000000 119.000000 124.000000 139.000000
          [,36]      [,37]      [,38]
[1,]   5.027778   7.076389   6.097222
[2,]  31.000000 106.000000 160.000000
[3,] 121.000000  87.000000 123.000000
> ylimits<-c(0.9*(min(preop,postop,na.rm=TRUE)),max(preop,postop, na.rm=TRUE))
> xlimits<-c(0,max(epi$withdrawaltime, na.rm=TRUE))
> xlabel<-"Days after surgery"
> plot(c(0,beforeandafterdata[1,1]),c(beforeandafterdata[2,1],
beforeandafterdata[3,1]),
type="l",col="black",xlim=xlimits,ylim=ylimits,ann=TRUE,axes=TRUE,xlab=xlabel,
main="Individual pre- and postoperative Multiplate TRAP",ylab="Area under curve
(AUC)",  cex.main=1.3, cex.lab=1.4,lwd=1)
>
> for (x in 2:38) {lines(c(0,beforeandafterdata[1,x]),
c(beforeandafterdata[2,x], beforeandafterdata[3,x]), type="l", col="black",
lwd=1)}
>
> #normal range - enter manually!
```

```
> lines(c(-.4,-.4), c(84,128), type="l", lwd=6, lty=3)
>
> #Average pre and postoperative values at t=0 and t=mean withdrawal time
> preopmean<-mean(epi$Prov1multiplateTRAP, na.rm=TRUE)
> postopmean<-mean(epi$Prov2multiplateTRAP, na.rm=TRUE)
> lines(c(0, meanwithdrawaltime), c(preopmean, postopmean), type="l", lwd=6,
lty=4)
>
> #Significance value for pre- to postop change - see t.test or Wilcoxon's.
> text(9,110,"N/S", cex=2)
>
>
>
>
> #Before and after diagrams for multiplateASPI
> preop<-epi$Prov1multiplateASPI
> postop<-epi$Prov2multiplateASPI
> preopdata<-t(matrix(preop))
> postopdata<-t(matrix(postop))
> withdrawaltime<-t(matrix(epi$withdrawaltime))
> meanwithdrawaltime<-mean(epi$withdrawaltime, na.rm=TRUE)
> y<-rbind(preop, postop)
> beforeandafterdata<-rbind(withdrawaltime, preopdata, postopdata)
> beforeandafterdata
          [,1]      [,2]       [,3]       [,4] [,5]      [,6]       [,7]
[1,]  11.13194  6.118056   8.145833   3.107639  3.1  10.07292   7.104167
[2,]  82.00000 81.000000  79.000000 103.000000 68.0  75.00000  48.000000
[3,] 130.00000 97.000000 123.000000 129.000000 85.0 144.00000 148.000000
          [,8]      [,9]     [,10]     [,11]     [,12]     [,13]      [,14]
[1,]  6.100694  2.201389  5.145833  5.069444  14.09722  5.131944   6.180556
[2,] 90.000000 58.000000 74.000000 83.000000  63.00000 68.000000  84.000000
[3,] 99.000000 55.000000 57.000000 87.000000 123.00000 81.000000 106.000000
     [,15]     [,16]     [,17]     [,18]     [,19] [,20]     [,21]      [,22]
[1,]    NA  2.121528  5.194444  15.15278  5.909722    NA  5.111111   4.958333
[2,]    52 66.000000 24.000000  35.00000 71.000000    76 70.000000  99.000000
[3,]    NA 21.000000  6.000000 163.00000 77.000000    NA 95.000000 107.000000
         [,23]     [,24]     [,25] [,26]     [,27]     [,28] [,29] [,30]
[1,]  5.145833  2.097222  2.180556    NA  5.059028  3.138889    NA    NA
[2,] 21.000000 88.000000 91.000000   135 64.000000 73.000000    77    55
[3,] 63.000000 78.000000 75.000000    NA 18.000000 27.000000    NA    NA
         [,31]      [,32]      [,33]     [,34]      [,35]      [,36]
[1,]  11.17361   6.222222   6.180556  7.104167   6.291667   5.027778
[2,]  93.00000  34.000000  99.000000 56.000000  75.000000  39.000000
[3,] 120.00000 125.000000 115.000000 83.000000 143.000000 109.000000
          [,37]      [,38]
[1,]   7.076389   6.097222
[2,]  72.000000 119.000000
[3,] 120.000000  96.000000
> ylimits<-c(0.9*(min(preop,postop,na.rm=TRUE)),max(preop,postop, na.rm=TRUE))
> xlimits<-c(0,max(epi$withdrawaltime, na.rm=TRUE))
> xlabel<-"Days after surgery"
> plot(c(0,beforeandafterdata[1,1]),c(beforeandafterdata[2,1],
beforeandafterdata[3,1]),
type="l",col="black",xlim=xlimits,ylim=ylimits,ann=TRUE,axes=TRUE,xlab=xlabel,
main="Individual pre- and postoperative Multiplate ASPI",ylab="Area under curve
(AUC)",  cex.main=1.3, cex.lab=1.4,lwd=1)
```

```
>
> for (x in 2:38) {lines(c(0,beforeandafterdata[1,x]),
c(beforeandafterdata[2,x], beforeandafterdata[3,x]), type="l", col="black",
lwd=1)}
>
> #normal range - enter manually!
> lines(c(-.4,-.4), c(71,115), type="l", lwd=6, lty=3)
>
> #Average pre and postoperative values at t=0 and t=mean withdrawal time
> preopmean<-mean(epi$Prov1multiplateASPI, na.rm=TRUE)
> postopmean<-mean(epi$Prov2multiplateASPI, na.rm=TRUE)
> lines(c(0, meanwithdrawaltime), c(preopmean, postopmean), type="l", lwd=6,
lty=4)
>
> #Significance value for pre- to postop change - see t.test or Wilcoxon's.
> text(9,90,"P<0.01", cex=2)
>
>
>
>
>
> #Before and after diagrams for PK
> preop<-epi$Prov1rutinPK
> postop<-epi$Prov2rutinPK
> preopdata<-t(matrix(preop))
> postopdata<-t(matrix(postop))
> withdrawaltime<-t(matrix(epi$withdrawaltime))
> meanwithdrawaltime<-mean(epi$withdrawaltime, na.rm=TRUE)
> y<-rbind(preop, postop)
> beforeandafterdata<-rbind(withdrawaltime, preopdata, postopdata)
> beforeandafterdata
         [,1]     [,2]     [,3]     [,4] [,5]     [,6]     [,7]     [,8]
[1,] 11.13194 6.118056 8.145833 3.107639  3.1 10.07292 7.104167 6.100694
[2,]  1.00000       NA 1.100000 1.100000  1.1  1.00000 1.300000 1.100000
[3,]  1.20000 1.100000 1.100000 1.200000  1.3  1.20000 1.300000 1.300000
         [,9]    [,10]    [,11]    [,12]    [,13]    [,14] [,15]    [,16]
[1,] 2.201389 5.145833 5.069444 14.09722 5.131944 6.180556    NA 2.121528
[2,] 0.900000 0.900000 1.000000  1.00000 1.000000 1.100000   0.9 1.000000
[3,] 1.000000 1.200000 1.200000  1.40000 1.100000 1.100000    NA 1.600000
        [,17]    [,18]    [,19] [,20]    [,21]    [,22]    [,23]    [,24]
[1,] 5.194444 15.15278 5.909722    NA 5.111111 4.958333 5.145833 2.097222
[2,] 1.100000  1.00000 1.000000     1 1.000000 1.000000 1.100000 0.900000
[3,] 1.100000  1.20000 1.100000    NA 1.100000 1.100000 1.200000 1.300000
        [,25] [,26]    [,27]    [,28] [,29] [,30]    [,31]    [,32]    [,33]
[1,] 2.180556    NA 5.059028 3.138889    NA    NA 11.17361 6.222222 6.180556
[2,]       NA   0.9 1.000000 1.100000    NA   1.1  1.00000 1.000000 1.100000
[3,] 1.000000   0.9 1.100000 1.500000   1.1    NA  1.00000 1.000000 1.100000
        [,34]    [,35]    [,36]    [,37]    [,38]
[1,] 7.104167 6.291667 5.027778 7.076389 6.097222
[2,]       NA 1.100000 1.100000 1.100000 0.900000
[3,] 1.000000 1.300000 1.100000 1.400000 1.000000
> ylimits<-c(0.9*(min(preop,postop,na.rm=TRUE)),max(preop,postop, na.rm=TRUE))
> xlimits<-c(0,max(epi$withdrawaltime, na.rm=TRUE))
> xlabel<-"Days after surgery"
> plot(c(0,beforeandafterdata[1,1]),c(beforeandafterdata[2,1],
beforeandafterdata[3,1]),
```

```
type="l",col="black",xlim=xlimits,ylim=ylimits,ann=TRUE,axes=TRUE,xlab=xlabel,
main="Individual pre- and postoperative PT-INR",ylab="PT-INR",  cex.main=1.3,
cex.lab=1.4,lwd=1)
>
> for (x in 2:38) {lines(c(0,beforeandafterdata[1,x]),
c(beforeandafterdata[2,x], beforeandafterdata[3,x]), type="l", col="black",
lwd=1)}
>
> #normal range - enter manually!
> lines(c(-.4,-.4), c(0.9,1.2), type="l", lwd=6, lty=3)
>
> #Average pre and postoperative values at t=0 and t=mean withdrawal time
> preopmean<-mean(epi$Prov1rutinPK, na.rm=TRUE)
> postopmean<-mean(epi$Prov2rutinPK, na.rm=TRUE)
> lines(c(0, meanwithdrawaltime), c(preopmean, postopmean), type="l", lwd=6,
lty=4)
>
> t.test(epi$Prov1rutinPK,epi$Prov2rutinPK, alternative =
c("two.sided"),paired=TRUE)

    Paired t-test

data:  epi$Prov1rutinPK and epi$Prov2rutinPK
t = -5.6176, df = 30, p-value = 4.077e-06
alternative hypothesis: true difference in means is not equal to 0
95 percent confidence interval:
 -0.20673186 -0.09649394
sample estimates:
mean of the differences
             -0.1516129

> wilcox.test(epi$Prov1rutinPK,epi$Prov2rutinPK, alternative =
c("two.sided"),paired=TRUE)

    Wilcoxon signed rank test with continuity correction

data:  epi$Prov1rutinPK and epi$Prov2rutinPK
V = 0, p-value = 3.92e-05
alternative hypothesis: true location shift is not equal to 0

Warning messages:
1: In wilcox.test.default(epi$Prov1rutinPK, epi$Prov2rutinPK, alternative =
c("two.sided"),  :
  cannot compute exact p-value with ties
2: In wilcox.test.default(epi$Prov1rutinPK, epi$Prov2rutinPK, alternative =
c("two.sided"),  :
  cannot compute exact p-value with zeroes
>
> #Significance value for pre- to postop change - see t.test or Wilcoxon's.
> text(8,1.45,"P<0.01", cex=2)
>
>
>
>
>
> #Before and after diagrams for APTT
```

```
> preop<-epi$Prov1rutinAPTT
> postop<-epi$Prov2rutinAPTT
> preopdata<-t(matrix(preop))
> postopdata<-t(matrix(postop))
> withdrawaltime<-t(matrix(epi$withdrawaltime))
> meanwithdrawaltime<-mean(epi$withdrawaltime, na.rm=TRUE)
> y<-rbind(preop, postop)
> beforeandafterdata<-rbind(withdrawaltime, preopdata, postopdata)
> beforeandafterdata
         [,1]      [,2]      [,3]      [,4] [,5]     [,6]      [,7]      [,8]
[1,] 11.13194  6.118056  8.145833  3.107639  3.1 10.07292  7.104167  6.100694
[2,] 26.00000        NA 34.000000 34.000000 29.0       NA 26.000000 26.000000
[3,] 30.00000 29.000000 33.000000 29.000000 35.0 32.00000 26.000000 34.000000
          [,9]     [,10]     [,11]    [,12]     [,13]     [,14] [,15]
[1,]  2.201389  5.145833  5.069444 14.09722  5.131944  6.180556    NA
[2,] 24.000000 26.000000 27.000000 24.00000 31.000000 26.000000    26
[3,] 25.000000 25.000000 35.000000 28.00000 30.000000 28.000000    NA
         [,16]     [,17]    [,18]     [,19] [,20]     [,21]     [,22]
[1,]  2.121528  5.194444 15.15278  5.909722    NA  5.111111  4.958333
[2,] 26.000000 30.000000 27.00000 31.000000    25 26.000000 27.000000
[3,] 43.000000 29.000000 26.00000 25.000000    NA 27.000000 30.000000
         [,23]     [,24]     [,25] [,26]     [,27]     [,28] [,29] [,30]
[1,]  5.145833  2.097222  2.180556    NA  5.059028  3.138889    NA    NA
[2,] 25.000000 25.000000        NA    29 24.000000 27.000000    NA    27
[3,] 27.000000 29.000000 28.000000    28 27.000000 32.000000    33    NA
        [,31]     [,32]     [,33]     [,34]     [,35]     [,36]     [,37]
[1,] 11.17361  6.222222  6.180556  7.104167  6.291667  5.027778  7.076389
[2,] 32.00000 22.000000 24.000000        NA 27.000000 24.000000 28.000000
[3,] 29.00000 24.000000 26.000000 25.000000 30.000000 26.000000 43.000000
         [,38]
[1,]  6.097222
[2,] 26.000000
[3,] 30.000000
> ylimits<-c(0.9*(min(preop,postop,na.rm=TRUE)),max(preop,postop, na.rm=TRUE))
> xlimits<-c(0,max(epi$withdrawaltime, na.rm=TRUE))
> xlabel<-"Days after surgery"
> plot(c(0,beforeandafterdata[1,1]),c(beforeandafterdata[2,1],
beforeandafterdata[3,1]),
type="l",col="black",xlim=xlimits,ylim=ylimits,ann=TRUE,axes=TRUE,xlab=xlabel,
main="Individual pre- and postoperative aPTT",ylab="aPTT (s)",  cex.main=1.3,
cex.lab=1.4,lwd=1)
>
> for (x in 2:38) {lines(c(0,beforeandafterdata[1,x]),
c(beforeandafterdata[2,x], beforeandafterdata[3,x]), type="l", col="black",
lwd=1)}
>
> #normal range - enter manually!
> lines(c(-.4,-.4), c(28,36), type="l", lwd=6, lty=3)
>
> #Average pre and postoperative values at t=0 and t=mean withdrawal time
> preopmean<-mean(epi$Prov1rutinAPTT, na.rm=TRUE)
> postopmean<-mean(epi$Prov2rutinAPTT, na.rm=TRUE)
> lines(c(0, meanwithdrawaltime), c(preopmean, postopmean), type="l", lwd=6,
lty=4)
>
> t.test(epi$Prov1rutinAPTT,epi$Prov2rutinAPTT, alternative =
```

```
c("two.sided"),paired=TRUE)

	Paired t-test

data:  epi$Prov1rutinAPTT and epi$Prov2rutinAPTT
t = -2.8253, df = 29, p-value = 0.008461
alternative hypothesis: true difference in means is not equal to 0
95 percent confidence interval:
 -4.3671924 -0.6994742
sample estimates:
mean of the differences
              -2.533333

> wilcox.test(epi$Prov1rutinAPTT,epi$Prov2rutinAPTT, alternative =
c("two.sided"),paired=TRUE)

	Wilcoxon signed rank test with continuity correction

data:  epi$Prov1rutinAPTT and epi$Prov2rutinAPTT
V = 89.5, p-value = 0.005653
alternative hypothesis: true location shift is not equal to 0

Warning messages:
1: In wilcox.test.default(epi$Prov1rutinAPTT, epi$Prov2rutinAPTT, alternative =
c("two.sided"),  :
  cannot compute exact p-value with ties
2: In wilcox.test.default(epi$Prov1rutinAPTT, epi$Prov2rutinAPTT, alternative =
c("two.sided"),  :
  cannot compute exact p-value with zeroes
>
> #Significance value for pre- to postop change - see t.test or Wilcoxon's.
> text(8,35,"P<0.01", cex=2)
>
>
>
>
>
> #Before and after diagrams for TPK
> dev.new(width=5)
> preop<-epi$Prov1rutinTPK
> postop<-epi$Prov2rutinTPK
> preopdata<-t(matrix(preop))
> postopdata<-t(matrix(postop))
> withdrawaltime<-t(matrix(epi$withdrawaltime))
> meanwithdrawaltime<-mean(epi$withdrawaltime, na.rm=TRUE)
> y<-rbind(preop, postop)
> beforeandafterdata<-rbind(withdrawaltime, preopdata, postopdata)
> beforeandafterdata
          [,1]       [,2]       [,3]       [,4]  [,5]     [,6]       [,7]
[1,]  11.13194   6.118056   8.145833   3.107639   3.1  10.07292   7.104167
[2,] 229.00000 224.000000 205.000000 477.000000 105.0 202.00000 131.000000
[3,]        NA 286.000000 312.000000 657.000000 131.0        NA 325.000000
           [,8]       [,9]      [,10]      [,11]     [,12]      [,13]
[1,]   6.100694   2.201389   5.145833   5.069444  14.09722   5.131944
[2,] 189.000000 305.000000 145.000000 221.000000 170.00000 169.000000
[3,] 262.000000 310.000000 162.000000 244.000000 351.00000 142.000000
```

```
          [,14] [,15]     [,16]     [,17]    [,18]     [,19] [,20]
[1,]   6.180556    NA  2.121528  5.194444 15.15278  5.909722    NA
[2,] 240.000000   132 269.000000 212.000000 224.00000 142.000000   159
[3,] 273.000000    NA 221.000000 309.000000 864.00000 216.000000    NA
          [,21]     [,22]     [,23]     [,24]     [,25] [,26]     [,27]
[1,]   5.111111   4.958333   5.145833   2.097222   2.180556    NA   5.059028
[2,] 153.000000 221.000000 171.000000 238.000000 237.000000   336 119.000000
[3,] 282.000000 248.000000 177.000000 140.000000 200.000000    NA 134.000000
          [,28] [,29] [,30]     [,31]     [,32]     [,33]     [,34]
[1,]   3.138889    NA    NA  11.17361   6.222222   6.180556   7.104167
[2,] 175.000000   251   293 179.00000 274.000000 194.000000 129.000000
[3,]  35.000000    NA    NA 337.00000 303.000000 280.000000 210.000000
          [,35]     [,36]     [,37]     [,38]
[1,]   6.291667   5.027778   7.076389   6.097222
[2,] 189.000000 196.000000 277.000000 316.000000
[3,] 286.000000 389.000000 370.000000 319.000000
> ylimits<-c(0.9*(min(preop,postop,na.rm=TRUE)),max(preop,postop, na.rm=TRUE))
> xlimits<-c(0,max(epi$withdrawaltime, na.rm=TRUE))
> xlabel<-"Days after surgery"
> plot(c(0,beforeandafterdata[1,1]),c(beforeandafterdata[2,1],
beforeandafterdata[3,1]),
type="l",col="black",xlim=xlimits,ylim=ylimits,ann=TRUE,axes=TRUE,xlab=xlabel,
main="Individual pre- and postoperative Plc",ylab="Plc (millions/ml)",
cex.main=1.3, cex.lab=1.4,lwd=1)
>
> for (x in 2:38) {lines(c(0,beforeandafterdata[1,x]),
c(beforeandafterdata[2,x], beforeandafterdata[3,x]), type="l", col="black",
lwd=1)}
>
> #normal range - enter manually!
> lines(c(-.4,-.4), c(145,280), type="l", code=0,lwd=2,lty=3)
Warning message:
In plot.xy(xy.coords(x, y), type = type, ...) :
  "code" is not a graphical parameter
>
> #Average pre and postoperative values at t=0 and t=mean withdrawal time
> preopmean<-mean(epi$Prov1rutinTPK, na.rm=TRUE)
> postopmean<-mean(epi$Prov2rutinTPK, na.rm=TRUE)
> lines(c(0, meanwithdrawaltime), c(preopmean, postopmean), type="l", lwd=6,
lty=4)
>
> t.test(epi$Prov1rutinTPK,epi$Prov2rutinTPK, alternative =
c("two.sided"),paired=TRUE)

	Paired t-test

data:  epi$Prov1rutinTPK and epi$Prov2rutinTPK
t = -3.0816, df = 30, p-value = 0.004385
alternative hypothesis: true difference in means is not equal to 0
95 percent confidence interval:
 -122.2372  -24.7951
sample estimates:
mean of the differences
              -73.51613

> wilcox.test(epi$Prov1rutinTPK,epi$Prov2rutinTPK, alternative =
```

```
c("two.sided"),paired=TRUE)

    Wilcoxon signed rank test with continuity correction

data:  epi$Prov1rutinTPK and epi$Prov2rutinTPK
V = 80.5, p-value = 0.001065
alternative hypothesis: true location shift is not equal to 0

Warning message:
In wilcox.test.default(epi$Prov1rutinTPK, epi$Prov2rutinTPK, alternative =
c("two.sided"),  :
  cannot compute exact p-value with ties
>
> #Significance value for pre- to postop change - see t.test or Wilcoxon's.
> text(8,400,"P<0.01", cex=2)
>
>
> ###Some means and SD's from the data
> mean(epi$Alb, na.rm=TRUE)
[1] 36.60714
> sd(epi$Alb, na.rm=TRUE)
[1] 3.715227
>
> mean(epi$withdrawaltime, na.rm=TRUE)
[1] 6.180324
> sd(epi$withdrawaltime, na.rm=TRUE)
[1] 3.152811
>
> mean(epi$oplangd, na.rm=TRUE)
[1] 10.34211
> sd(epi$oplangd, na.rm=TRUE)
[1] 11.75719
>
> mean(epi$PEROPbloedning, na.rm=TRUE)
[1] 661.1111
> sd(epi$PEROPbloedning, na.rm=TRUE)
[1] 784.5027
>
> mean(epi$Prov1rutinBIL, na.rm=TRUE)
[1] 14.05263
> sd(epi$Prov1rutinBIL, na.rm=TRUE)
[1] 21.78016
> mean(epi$Prov2rutinBIL, na.rm=TRUE)
[1] 10.51613
> sd(epi$Prov2rutinBIL, na.rm=TRUE)
[1] 16.47801
> t.test(epi$Prov1rutinBIL, epi$Prov2rutinBIL, alternative =
c("two.sided"),paired=TRUE)

    Paired t-test

data:  epi$Prov1rutinBIL and epi$Prov2rutinBIL
t = 2.5067, df = 30, p-value = 0.01783
alternative hypothesis: true difference in means is not equal to 0
95 percent confidence interval:
 0.6634288 6.4978615
```

```
sample estimates:
mean of the differences
               3.580645

> wilcox.test(epi$Prov1rutinBIL, epi$Prov2rutinBIL, alternative =
c("two.sided"),paired=TRUE)

    Wilcoxon signed rank test with continuity correction

data:  epi$Prov1rutinBIL and epi$Prov2rutinBIL
V = 258.5, p-value = 0.009943
alternative hypothesis: true location shift is not equal to 0

Warning messages:
1: In wilcox.test.default(epi$Prov1rutinBIL, epi$Prov2rutinBIL, alternative =
c("two.sided"),  :
  cannot compute exact p-value with ties
2: In wilcox.test.default(epi$Prov1rutinBIL, epi$Prov2rutinBIL, alternative =
c("two.sided"),  :
  cannot compute exact p-value with zeroes
>
> mean(epi$Prov1rutinGT, na.rm=TRUE)
[1] 1.306757
> sd(epi$Prov1rutinGT, na.rm=TRUE)
[1] 1.997771
> mean(epi$Prov2rutinGT, na.rm=TRUE)
[1] 2.11
> sd(epi$Prov2rutinGT, na.rm=TRUE)
[1] 1.872609
> t.test(epi$Prov1rutinGT, epi$Prov2rutinGT, alternative =
c("two.sided"),paired=TRUE)

    Paired t-test

data:  epi$Prov1rutinGT and epi$Prov2rutinGT
t = -2.4204, df = 30, p-value = 0.02177
alternative hypothesis: true difference in means is not equal to 0
95 percent confidence interval:
 -1.6427357 -0.1391998
sample estimates:
mean of the differences
             -0.8909677

> wilcox.test(epi$Prov1rutinGT, epi$Prov2rutinGT, alternative =
c("two.sided"),paired=TRUE)

    Wilcoxon signed rank test with continuity correction

data:  epi$Prov1rutinGT and epi$Prov2rutinGT
V = 95, p-value = 0.004833
alternative hypothesis: true location shift is not equal to 0

Warning messages:
1: In wilcox.test.default(epi$Prov1rutinGT, epi$Prov2rutinGT, alternative =
c("two.sided"),  :
  cannot compute exact p-value with ties
```

```
2: In wilcox.test.default(epi$Prov1rutinGT, epi$Prov2rutinGT, alternative =
c("two.sided"),  :
  cannot compute exact p-value with zeroes
>
> mean(epi$Prov1rutinCRP, na.rm=TRUE)
[1] 6.784211
> sd(epi$Prov1rutinCRP, na.rm=TRUE)
[1] 9.436312
> mean(epi$Prov2rutinCRP, na.rm=TRUE)
[1] 91.96667
> sd(epi$Prov2rutinCRP, na.rm=TRUE)
[1] 59.82762
> t.test(epi$Prov1rutinCRP, epi$Prov2rutinCRP, alternative =
c("two.sided"),paired=TRUE)

	Paired t-test

data:  epi$Prov1rutinCRP and epi$Prov2rutinCRP
t = -7.5742, df = 29, p-value = 2.38e-08
alternative hypothesis: true difference in means is not equal to 0
95 percent confidence interval:
 -107.688  -61.896
sample estimates:
mean of the differences
                -84.792

> wilcox.test(epi$Prov1rutinCRP, epi$Prov2rutinCRP, alternative =
c("two.sided"),paired=TRUE)

	Wilcoxon signed rank test

data:  epi$Prov1rutinCRP and epi$Prov2rutinCRP
V = 0, p-value = 1.863e-09
alternative hypothesis: true location shift is not equal to 0

>
> mean(epi$Prov1rutinKREA, na.rm=TRUE)
[1] 73.37838
> sd(epi$Prov1rutinKREA, na.rm=TRUE)
[1] 19.00928
> mean(epi$Prov2rutinKREA, na.rm=TRUE)
[1] 65.22581
> sd(epi$Prov2rutinKREA, na.rm=TRUE)
[1] 18.27149
> t.test(epi$Prov1rutinKREA, epi$Prov2rutinKREA, alternative =
c("two.sided"),paired=TRUE)

	Paired t-test

data:  epi$Prov1rutinKREA and epi$Prov2rutinKREA
t = 3.0497, df = 29, p-value = 0.004856
alternative hypothesis: true difference in means is not equal to 0
95 percent confidence interval:
 1.602930 8.130403
sample estimates:
mean of the differences
```

```
               4.866667

> wilcox.test(epi$Prov1rutinKREA, epi$Prov2rutinKREA, alternative =
c("two.sided"),paired=TRUE)

	Wilcoxon signed rank test with continuity correction

data:  epi$Prov1rutinKREA and epi$Prov2rutinKREA
V = 318, p-value = 0.009062
alternative hypothesis: true location shift is not equal to 0

Warning messages:
1: In wilcox.test.default(epi$Prov1rutinKREA, epi$Prov2rutinKREA, alternative =
c("two.sided"),  :
  cannot compute exact p-value with ties
2: In wilcox.test.default(epi$Prov1rutinKREA, epi$Prov2rutinKREA, alternative =
c("two.sided"),  :
  cannot compute exact p-value with zeroes
> 
> mean(epi$PEROPbloedning, na.rm=TRUE)
[1] 661.1111
> sd(epi$PEROPbloedning, na.rm=TRUE)
[1] 784.5027
> 
> mean(epi$DRAENmaengd, na.rm=TRUE)
[1] 430.2273
> sd(epi$DRAENmaengd, na.rm=TRUE)
[1] 355.2178
> 
> ####Hb
> mean(epi$Prov1rutinHB, na.rm=TRUE)
[1] 119.5
> sd(epi$Prov1rutinHB, na.rm=TRUE)
[1] 16.14922
> 
> mean(epi$Prov2rutinHB, na.rm=TRUE)
[1] 112.7576
> sd(epi$Prov2rutinHB, na.rm=TRUE)
[1] 11.9714
> min(epi$Prov2rutinHB, na.rm=TRUE)
[1] 87
> max(epi$Prov2rutinHB, na.rm=TRUE)
[1] 147
> 
> ###Correlations
> #Correlation between withdrawal time and EXTEM-MCF
> plot(epi$withdrawaltime, epi$Prov2extemMCF, xlab="Day of epidural
withdrawal", ylab = "EXTEM-MCF (mm)", abline(lm(epi$Prov2extemMCF~epi
$withdrawaltime)),cex.main=1.3, cex.lab=1.4, ylim=c(0,80))
> cor.test(epi$withdrawaltime, epi$Prov2extemMCF, use="complete.obs",
method="pearson")

	Pearson's product-moment correlation

data:  epi$withdrawaltime and epi$Prov2extemMCF
t = 2.9901, df = 31, p-value = 0.005423
```

```
alternative hypothesis: true correlation is not equal to 0
95 percent confidence interval:
 0.1549956 0.7023547
sample estimates:
      cor
0.4731287

> cor.test(epi$withdrawaltime, epi$Prov2extemMCF, use="complete.obs",
method="spearman")

	Spearman's rank correlation rho

data:  epi$withdrawaltime and epi$Prov2extemMCF
S = 2929, p-value = 0.0024
alternative hypothesis: true rho is not equal to 0
sample estimates:
      rho
0.5105266

Warning message:
In cor.test.default(epi$withdrawaltime, epi$Prov2extemMCF, use =
"complete.obs",  :
  Cannot compute exact p-value with ties
> text(x=12, y=60, "P<0.01", pos=3, cex=2)
> text(x=12, y=55, "r=0.47", pos=3, cex=2)
>
> #Correlation between withdrawal time and FIBTEM-MCF
> plot(epi$withdrawaltime, epi$Prov2fibtemMCF, xlab="Day of epidural
withdrawal", ylab = "FIBTEM-MCF (mm)", abline(lm(epi$Prov2fibtemMCF~epi
$withdrawaltime)),cex.main=1.3, cex.lab=1.4, ylim=c(0,80))
> cor.test(epi$withdrawaltime, epi$Prov2fibtemMCF, use="complete.obs",
method="pearson")

	Pearson's product-moment correlation

data:  epi$withdrawaltime and epi$Prov2fibtemMCF
t = 4.3392, df = 31, p-value = 0.0001412
alternative hypothesis: true correlation is not equal to 0
95 percent confidence interval:
 0.3439931 0.7910728
sample estimates:
      cor
0.6147076

> cor.test(epi$withdrawaltime, epi$Prov2fibtemMCF, use="complete.obs",
method="spearman")

	Spearman's rank correlation rho

data:  epi$withdrawaltime and epi$Prov2fibtemMCF
S = 2088.6, p-value = 4.093e-05
alternative hypothesis: true rho is not equal to 0
sample estimates:
      rho
0.6509734
```

```
Warning message:
In cor.test.default(epi$withdrawaltime, epi$Prov2fibtemMCF, use =
"complete.obs",  :
  Cannot compute exact p-value with ties
> text(x=12, y=70, "P<0.01", pos=3, cex=2)
> text(x=12, y=65, "r=0.61", pos=3, cex=2)
>
> #Correlation between withdrawal time and INTEM-MCF
> plot(epi$withdrawaltime, epi$Prov2intemMCF, xlab="Day of epidural
withdrawal", ylab = "INTEM-MCF (mm)", abline(lm(epi$Prov2intemMCF~epi
$withdrawaltime)),cex.main=1.3, cex.lab=1.4, ylim=c(0,80))
> cor.test(epi$withdrawaltime, epi$Prov2intemMCF, use="complete.obs",
method="pearson")

    Pearson's product-moment correlation

data:  epi$withdrawaltime and epi$Prov2intemMCF
t = 2.678, df = 31, p-value = 0.01174
alternative hypothesis: true correlation is not equal to 0
95 percent confidence interval:
 0.1058989 0.6761435
sample estimates:
      cor
0.4334494

> cor.test(epi$withdrawaltime, epi$Prov2intemMCF, use="complete.obs",
method="spearman")

    Spearman's rank correlation rho

data:  epi$withdrawaltime and epi$Prov2intemMCF
S = 1875.2, p-value = 1.023e-05
alternative hypothesis: true rho is not equal to 0
sample estimates:
      rho
0.6866393

Warning message:
In cor.test.default(epi$withdrawaltime, epi$Prov2intemMCF, use =
"complete.obs",  :
  Cannot compute exact p-value with ties
> text(x=12, y=60, "P<0.05", pos=3, cex=2)
> text(x=12, y=55, "r=0.43", pos=3, cex=2)
>
> #Correlation between withdrawal time and HEPTEM-MCF
> plot(epi$withdrawaltime, epi$Prov2heptemMCF, xlab="Day of epidural
withdrawal", ylab = "HEPTEM-MCF (mm)", abline(lm(epi$Prov2heptemMCF~epi
$withdrawaltime)),cex.main=1.3, cex.lab=1.4, ylim=c(0,80))
> cor.test(epi$withdrawaltime, epi$Prov2heptemMCF, use="complete.obs",
method="pearson")

    Pearson's product-moment correlation

data:  epi$withdrawaltime and epi$Prov2heptemMCF
t = 2.8901, df = 30, p-value = 0.007092
alternative hypothesis: true correlation is not equal to 0
```

```
95 percent confidence interval:
 0.1409112 0.7012560
sample estimates:
      cor 
0.4666751 

> cor.test(epi$withdrawaltime, epi$Prov2heptemMCF, use="complete.obs", 
method="spearman")

	Spearman's rank correlation rho

data:  epi$withdrawaltime and epi$Prov2heptemMCF
S = 2716.7, p-value = 0.003411
alternative hypothesis: true rho is not equal to 0
sample estimates:
      rho 
0.5020796 

Warning message:
In cor.test.default(epi$withdrawaltime, epi$Prov2heptemMCF, use = 
"complete.obs",  :
  Cannot compute exact p-value with ties
> text(x=15, y=70, "**", pos=3, cex=2)
> text(x=12, y=60, "P<0.01", pos=3, cex=2)
> text(x=12, y=55, "r=0.47", pos=3, cex=2)
> 
> #Correlation between withdrawal time and Prov2multiplateADP
> plot(epi$withdrawaltime, epi$Prov2multiplateADP, xlab="Day of epidural 
withdrawal", ylab = "Multiplate-ADP (Area under curve)", abline(lm(epi
$Prov2heptemMCF~epi$withdrawaltime)),cex.main=1.3, cex.lab=1.4,ylim=c(30,160))
> cor.test(epi$withdrawaltime, epi$Prov2multiplateADP, use="complete.obs", 
method="pearson")

	Pearson's product-moment correlation

data:  epi$withdrawaltime and epi$Prov2multiplateADP
t = 3.6191, df = 31, p-value = 0.00104
alternative hypothesis: true correlation is not equal to 0
95 percent confidence interval:
 0.2481091 0.7482973
sample estimates:
      cor 
0.5449964 

> cor.test(epi$withdrawaltime, epi$Prov2multiplateADP, use="complete.obs", 
method="spearman")

	Spearman's rank correlation rho

data:  epi$withdrawaltime and epi$Prov2multiplateADP
S = 2357.4, p-value = 0.0001855
alternative hypothesis: true rho is not equal to 0
sample estimates:
     rho 
0.606053 
```

```
Warning message:
In cor.test.default(epi$withdrawaltime, epi$Prov2multiplateADP,  :
  Cannot compute exact p-value with ties
> text(x=13, y=60, "P<0.01", pos=3, cex=2)
> text(x=13, y=50, "r=0.54", pos=3, cex=2)
>
>
> #Correlation between withdrawal time and Prov2multiplateCOL
> plot(epi$withdrawaltime, epi$Prov2multiplateCOL, xlab="Day of epidural
withdrawal", ylab = "Multiplate-COL (Area under curve)", abline(lm(epi
$Prov2multiplateCOL~epi$withdrawaltime)),cex.main=1.3,
cex.lab=1.4,ylim=c(30,160))
> cor.test(epi$withdrawaltime, epi$Prov2multiplateCOL, use="complete.obs",
method="pearson")

    Pearson's product-moment correlation

data:  epi$withdrawaltime and epi$Prov2multiplateCOL
t = 4.3567, df = 31, p-value = 0.0001344
alternative hypothesis: true correlation is not equal to 0
95 percent confidence interval:
 0.3461759 0.7919982
sample estimates:
      cor
0.6162468

> cor.test(epi$withdrawaltime, epi$Prov2multiplateCOL, use="complete.obs",
method="spearman")

    Spearman's rank correlation rho

data:  epi$withdrawaltime and epi$Prov2multiplateCOL
S = 2142.8, p-value = 5.659e-05
alternative hypothesis: true rho is not equal to 0
sample estimates:
      rho
0.6419196

Warning message:
In cor.test.default(epi$withdrawaltime, epi$Prov2multiplateCOL,  :
  Cannot compute exact p-value with ties
> text(x=13, y=60, "P<0.01", pos=3, cex=2)
> text(x=13, y=50, "r=0.61", pos=3, cex=2)
>
> #Correlation between withdrawal time and Prov2multiplateTRAP
> plot(epi$withdrawaltime, epi$Prov2multiplateCOL, xlab="Day of epidural
withdrawal", ylab = "Multiplate-TRAP (Area under curve)", abline(lm(epi
$Prov2multiplateTRAP~epi$withdrawaltime)),cex.main=1.3,
cex.lab=1.4,ylim=c(30,160))
> cor.test(epi$withdrawaltime, epi$Prov2multiplateTRAP, use="complete.obs",
method="pearson")

    Pearson's product-moment correlation

data:  epi$withdrawaltime and epi$Prov2multiplateTRAP
t = 3.5038, df = 31, p-value = 0.001419
```

```
alternative hypothesis: true correlation is not equal to 0
95 percent confidence interval:
 0.2316643 0.7405179
sample estimates:
      cor
0.5326132

> cor.test(epi$withdrawaltime, epi$Prov2multiplateTRAP, use="complete.obs",
method="spearman")

	Spearman's rank correlation rho

data:  epi$withdrawaltime and epi$Prov2multiplateTRAP
S = 2114.2, p-value = 4.777e-05
alternative hypothesis: true rho is not equal to 0
sample estimates:
      rho
0.6466901

Warning message:
In cor.test.default(epi$withdrawaltime, epi$Prov2multiplateTRAP,  :
  Cannot compute exact p-value with ties
> text(x=13, y=60, "P<0.01", pos=3, cex=2)
> text(x=13, y=50, "r=0.53", pos=3, cex=2)
>
> #Correlation between withdrawal time and Prov2multiplateASPI
> plot(epi$withdrawaltime, epi$Prov2multiplateCOL, xlab="Day of epidural
withdrawal", ylab = "Multiplate-ASPI (Area under curve)", abline(lm(epi
$Prov2multiplateASPI~epi$withdrawaltime)),cex.main=1.3,
cex.lab=1.4,ylim=c(30,160))
> cor.test(epi$withdrawaltime, epi$Prov2multiplateASPI, use="complete.obs",
method="pearson")

	Pearson's product-moment correlation

data:  epi$withdrawaltime and epi$Prov2multiplateASPI
t = 4.3702, df = 31, p-value = 0.0001293
alternative hypothesis: true correlation is not equal to 0
95 percent confidence interval:
 0.3478537 0.7927082
sample estimates:
      cor
0.6174285

> cor.test(epi$withdrawaltime, epi$Prov2multiplateASPI, use="complete.obs",
method="spearman")

	Spearman's rank correlation rho

data:  epi$withdrawaltime and epi$Prov2multiplateASPI
S = 1882.3, p-value = 1.075e-05
alternative hypothesis: true rho is not equal to 0
sample estimates:
      rho
0.6854468
```

```
Warning message:
In cor.test.default(epi$withdrawaltime, epi$Prov2multiplateASPI,  :
  Cannot compute exact p-value with ties
> text(x=13, y=60, "P<0.01", pos=3, cex=2)
> text(x=13, y=50, "r=0.61", pos=3, cex=2)
>
> #Correlation between Plc and multiplate ADP
> dev.new(width=5)
> plot(epi$Prov2rutinTPK, epi$Prov2multiplateADP,main="Multiplate ADP AUC vs
Plc", xlab="Platelet count (millions/mL)", ylab = "Multiplate-ADP (Area under
curve)", abline(lm(epi$Prov2multiplateADP~epi$Prov2rutinTPK)),cex.main=1.3,
cex.lab=1.4,ylim=c(30,160))
> cor.test(epi$Prov2rutinTPK, epi$Prov2multiplateADP, use="complete.obs",
method="pearson")

	Pearson's product-moment correlation

data:  epi$Prov2rutinTPK and epi$Prov2multiplateADP
t = 4.0675, df = 29, p-value = 0.0003329
alternative hypothesis: true correlation is not equal to 0
95 percent confidence interval:
 0.3158253 0.7886309
sample estimates:
      cor
0.6027158

> cor.test(epi$Prov2rutinTPK, epi$Prov2multiplateADP, use="complete.obs",
method="spearman")

	Spearman's rank correlation rho

data:  epi$Prov2rutinTPK and epi$Prov2multiplateADP
S = 2328.7, p-value = 0.00214
alternative hypothesis: true rho is not equal to 0
sample estimates:
      rho
0.5305094

Warning message:
In cor.test.default(epi$Prov2rutinTPK, epi$Prov2multiplateADP, use =
"complete.obs",  :
  Cannot compute exact p-value with ties
> text(x=700, y=80, "P<0.01", pos=3, cex=2)
> text(x=700, y=70, "r=0.60", pos=3, cex=2)
> arrows(50,57,,113,code=0,lwd=2,lty=3)
>
>
>
> #Correlation between Plc and multiplate COL
> plot(epi$Prov2rutinTPK, epi$Prov2multiplateCOL, xlab="Platelet count
(millions/mL)", ylab = "Multiplate-COL (Area under curve)", abline(lm(epi
$Prov2multiplateCOL~epi$Prov2rutinTPK)),cex.main=1.3,
cex.lab=1.4,ylim=c(30,160))
> cor.test(epi$Prov2rutinTPK, epi$Prov2multiplateCOL, use="complete.obs",
method="pearson")
```

```
	Pearson's product-moment correlation

data:  epi$Prov2rutinTPK and epi$Prov2multiplateCOL
t = 5.9174, df = 29, p-value = 1.998e-06
alternative hypothesis: true correlation is not equal to 0
95 percent confidence interval:
 0.5220598 0.8667749
sample estimates:
      cor 
0.7395863 

> cor.test(epi$Prov2rutinTPK, epi$Prov2multiplateCOL, use="complete.obs", 
method="spearman")

	Spearman's rank correlation rho

data:  epi$Prov2rutinTPK and epi$Prov2multiplateCOL
S = 1362.2, p-value = 3.902e-06
alternative hypothesis: true rho is not equal to 0
sample estimates:
      rho 
0.7253656 

Warning message:
In cor.test.default(epi$Prov2rutinTPK, epi$Prov2multiplateCOL, use = 
"complete.obs",  :
  Cannot compute exact p-value with ties
> text(x=700, y=100, "P<0.01", pos=3, cex=2)
> text(x=700, y=90, "r=0.74", pos=3, cex=2)
> 
> #Correlation between Plc and multiplate TRAP
> plot(epi$Prov2rutinTPK, epi$Prov2multiplateTRAP, xlab="Platelet count 
(millions/mL)", ylab = "Multiplate-TRAP (Area under curve)", abline(lm(epi
$Prov2multiplateTRAP~epi$Prov2rutinTPK)),cex.main=1.3, 
cex.lab=1.4,ylim=c(30,160))
> cor.test(epi$Prov2rutinTPK, epi$Prov2multiplateTRAP, use="complete.obs", 
method="pearson")

	Pearson's product-moment correlation

data:  epi$Prov2rutinTPK and epi$Prov2multiplateTRAP
t = 4.1616, df = 29, p-value = 0.0002575
alternative hypothesis: true correlation is not equal to 0
95 percent confidence interval:
 0.3282701 0.7938231
sample estimates:
     cor 
0.611483 

> cor.test(epi$Prov2rutinTPK, epi$Prov2multiplateTRAP, use="complete.obs", 
method="spearman")

	Spearman's rank correlation rho

data:  epi$Prov2rutinTPK and epi$Prov2multiplateTRAP
S = 1792.7, p-value = 0.0001108
```

```
alternative hypothesis: true rho is not equal to 0
sample estimates:
      rho 
0.6385724 

Warning message:
In cor.test.default(epi$Prov2rutinTPK, epi$Prov2multiplateTRAP,  :
  Cannot compute exact p-value with ties
> text(x=700, y=100, "P<0.01", pos=3, cex=2)
> text(x=700, y=90, "r=0.61", pos=3, cex=2)
> 
> 
> #Correlation between Plc and multiplate ASPI
> plot(epi$Prov2rutinTPK, epi$Prov2multiplateASPI, xlab="Platelet count 
(millions/mL)", ylab = "Multiplate-ASPI (Area under curve)", abline(lm(epi
$Prov2multiplateASPI~epi$Prov2rutinTPK)),cex.main=1.3, 
cex.lab=1.4,ylim=c(30,160))
> cor.test(epi$Prov2rutinTPK, epi$Prov2multiplateASPI, use="complete.obs", 
method="pearson")

	Pearson's product-moment correlation

data:  epi$Prov2rutinTPK and epi$Prov2multiplateASPI
t = 4.2389, df = 29, p-value = 0.0002084
alternative hypothesis: true correlation is not equal to 0
95 percent confidence interval:
 0.3383307 0.7979716
sample estimates:
      cor 
0.6185201 

> cor.test(epi$Prov2rutinTPK, epi$Prov2multiplateASPI, use="complete.obs", 
method="spearman")

	Spearman's rank correlation rho

data:  epi$Prov2rutinTPK and epi$Prov2multiplateASPI
S = 1444.9, p-value = 8.137e-06
alternative hypothesis: true rho is not equal to 0
sample estimates:
      rho 
0.7086821 

Warning message:
In cor.test.default(epi$Prov2rutinTPK, epi$Prov2multiplateASPI,  :
  Cannot compute exact p-value with ties
> text(x=700, y=100, "P<0.01", pos=3, cex=2)
> text(x=700, y=90, "r=0.62", pos=3, cex=2)
> 
> 
> #Correlation between d-dimer and FIBTEM-Li60
> plot(epi$DDpostop, epi$Prov2fibtemLI60, xlab="Postoperative d-dimer (mg/L)", 
ylab = "FIBTEM-Li60", abline(lm(epi$Prov2fibtemLI60 ~epi
$DDpostop)),cex.main=1.3, cex.lab=1.4)
> cor.test(epi$DDpostop, epi$Prov2fibtemLI60, use="complete.obs", 
method="pearson")
```

```
    Pearson's product-moment correlation

data:  epi$DDpostop and epi$Prov2fibtemLI60
t = -3.6528, df = 28, p-value = 0.001057
alternative hypothesis: true correlation is not equal to 0
95 percent confidence interval:
 -0.7706403 -0.2613083
sample estimates:
       cor
-0.5680962

> cor.test(epi$DDpostop, epi$Prov2fibtemLI60, use="complete.obs",
method="spearman")

    Spearman's rank correlation rho

data:  epi$DDpostop and epi$Prov2fibtemLI60
S = 6870.7, p-value = 0.002678
alternative hypothesis: true rho is not equal to 0
sample estimates:
       rho
-0.5285284

Warning message:
In cor.test.default(epi$DDpostop, epi$Prov2fibtemLI60, use = "complete.obs",  :
  Cannot compute exact p-value with ties
>
> text(x=4.5, y=98, "P<0.01", pos=3, cex=2)
> text(x=4.5, y=97, "r=-0.56", pos=3, cex=2)
>
> #Correlation between d-dimer and EXTEM-Li60
> plot(epi$DDpostop, epi$Prov2extemLI60, xlab="Postoperative d-dimer", ylab =
"EXTEM-Li60", abline(lm(epi$Prov2extemLI60 ~epi$DDpostop)),cex.main=1.3,
cex.lab=1.4)
> cor.test(epi$DDpostop, epi$Prov2extemLI60, use="complete.obs",
method="pearson")

    Pearson's product-moment correlation

data:  epi$DDpostop and epi$Prov2extemLI60
t = -1.8044, df = 29, p-value = 0.08157
alternative hypothesis: true correlation is not equal to 0
95 percent confidence interval:
 -0.60404504  0.04128158
sample estimates:
       cor
-0.3177058

> cor.test(epi$DDpostop, epi$Prov2extemLI60, use="complete.obs",
method="spearman")

    Spearman's rank correlation rho

data:  epi$DDpostop and epi$Prov2extemLI60
S = 5277.7, p-value = 0.7321
```

```
alternative hypothesis: true rho is not equal to 0
sample estimates:
        rho
-0.06405276

Warning message:
In cor.test.default(epi$DDpostop, epi$Prov2extemLI60, use = "complete.obs",  :
  Cannot compute exact p-value with ties
> text(x=4.5, y=93, "N/S", pos=3, cex=2)
>
> #Correlation between d-dimer and INTEM-Li60
> plot(epi$DDpostop, epi$Prov2intemLI60, xlab="Postoperative d-dimer", ylab =
"INTEM-Li60", abline(lm(epi$Prov2intemLI60 ~epi$DDpostop)),cex.main=1.3,
cex.lab=1.4)
> cor.test(epi$DDpostop, epi$Prov2intemLI60, use="complete.obs",
method="pearson")

    Pearson's product-moment correlation

data:  epi$DDpostop and epi$Prov2intemLI60
t = 0.8787, df = 26, p-value = 0.3876
alternative hypothesis: true correlation is not equal to 0
95 percent confidence interval:
 -0.2170022  0.5105531
sample estimates:
      cor
0.1698233

> cor.test(epi$DDpostop, epi$Prov2intemLI60, use="complete.obs",
method="spearman")

    Spearman's rank correlation rho

data:  epi$DDpostop and epi$Prov2intemLI60
S = 2773, p-value = 0.2165
alternative hypothesis: true rho is not equal to 0
sample estimates:
      rho
0.2410964

Warning message:
In cor.test.default(epi$DDpostop, epi$Prov2intemLI60, use = "complete.obs",  :
  Cannot compute exact p-value with ties
> text(x=4.5, y=93, "N/S", pos=3, cex=2)
>
> #Correlation between withdrawal time and D-Dimer
> plot(epi$withdrawaltime, epi$DDpostop, xlab="Day of epidural withdrawal",
ylab = "D-dimer", abline(lm(epi$DDpostop~epi$withdrawaltime)),cex.main=1.3,
cex.lab=1.4)
> cor.test(epi$withdrawaltime, epi$DDpostop, use="complete.obs",
method="pearson")

    Pearson's product-moment correlation

data:  epi$withdrawaltime and epi$DDpostop
t = 2.6428, df = 29, p-value = 0.01312
```

```
alternative hypothesis: true correlation is not equal to 0
95 percent confidence interval:
 0.1021768 0.6875695
sample estimates:
     cor 
0.440566 

> cor.test(epi$withdrawaltime, epi$DDpostop, use="complete.obs", 
method="spearman")

	Spearman's rank correlation rho

data:  epi$withdrawaltime and epi$DDpostop
S = 3224.5, p-value = 0.05365
alternative hypothesis: true rho is not equal to 0
sample estimates:
      rho 
0.3499042 

Warning message:
In cor.test.default(epi$withdrawaltime, epi$DDpostop, use = "complete.obs",  :
  Cannot compute exact p-value with ties
> text(x=13, y=5, "P=0.05", pos=3, cex=2)
> text(x=13, y=4.5, "rho=0.34", pos=3, cex=2)
> 
> hist(epi$DDpostop)
> hist(epi$Prov2fibtemLI60)
> 
> #Correlation between withdrawal time and Fibtem-Li60
> plot(epi$withdrawaltime, epi$Prov2fibtemLI60, xlab="Day of epidural 
withdrawal", ylab = "FIBTEM-Li60", abline(lm(epi$Prov2fibtemLI60~epi
$withdrawaltime)),cex.main=1.3, cex.lab=1.4)
> cor.test(epi$withdrawaltime, epi$Prov2fibtemLI60, use="complete.obs", 
method="pearson")

	Pearson's product-moment correlation

data:  epi$withdrawaltime and epi$Prov2fibtemLI60
t = -4.9153, df = 30, p-value = 2.959e-05
alternative hypothesis: true correlation is not equal to 0
95 percent confidence interval:
 -0.8245592 -0.4161145
sample estimates:
       cor 
-0.6678986 

> cor.test(epi$withdrawaltime, epi$Prov2fibtemLI60, use="complete.obs", 
method="spearman")

	Spearman's rank correlation rho

data:  epi$withdrawaltime and epi$Prov2fibtemLI60
S = 9239.1, p-value = 1.085e-05
alternative hypothesis: true rho is not equal to 0
sample estimates:
       rho 
```

```
-0.6933778

Warning message:
In cor.test.default(epi$withdrawaltime, epi$Prov2fibtemLI60, use =
"complete.obs",  :
  Cannot compute exact p-value with ties
> text(x=13, y=5, "P<0.01", pos=3, cex=2)
> text(x=13, y=4.5, "r=-0.67", pos=3, cex=2)
>
>
> #Correlation between withdrawal time and CRP
> plot(epi$withdrawaltime, epi$Prov2rutinCRP, xlab="Day of epidural
withdrawal", ylab = "CRP", abline(lm(epi$Prov2rutinCRP~epi
$withdrawaltime)),cex.main=1.3, cex.lab=1.4)
> cor.test(epi$withdrawaltime, epi$Prov2rutinCRP, use="complete.obs",
method="pearson")

	Pearson's product-moment correlation

data:  epi$withdrawaltime and epi$Prov2rutinCRP
t = -1.6047, df = 28, p-value = 0.1198
alternative hypothesis: true correlation is not equal to 0
95 percent confidence interval:
 -0.58890747  0.07823845
sample estimates:
       cor
-0.2902109

> cor.test(epi$withdrawaltime, epi$Prov2rutinCRP, use="complete.obs",
method="spearman")

	Spearman's rank correlation rho

data:  epi$withdrawaltime and epi$Prov2rutinCRP
S = 5519.1, p-value = 0.226
alternative hypothesis: true rho is not equal to 0
sample estimates:
       rho
-0.2278242

Warning message:
In cor.test.default(epi$withdrawaltime, epi$Prov2rutinCRP, use =
"complete.obs",  :
  Cannot compute exact p-value with ties
> text(x=13, y=150, "N/S", pos=3, cex=2)
>
> #Correlation between DDimer and CRP
> plot(epi$Prov2rutinCRP, epi$DDpostop, xlab="CRP", ylab = "D-dimer",
abline(lm(epi$DDpostop~epi$Prov2rutinCRP)),cex.main=1.3, cex.lab=1.4,
xlim=c(0,200), ylim=c(0,4))
> cor.test(epi$DDpostop, epi$Prov2rutinCRP, use="complete.obs",
method="pearson")

	Pearson's product-moment correlation

data:  epi$DDpostop and epi$Prov2rutinCRP
```

```
t = 1.6004, df = 26, p-value = 0.1216
alternative hypothesis: true correlation is not equal to 0
95 percent confidence interval:
 -0.08287968  0.60494847
sample estimates:
      cor 
0.2994566 

> cor.test(epi$withdrawaltime, epi$Prov2rutinCRP, use="complete.obs",
method="spearman")

	Spearman's rank correlation rho

data:  epi$withdrawaltime and epi$Prov2rutinCRP
S = 5519.1, p-value = 0.226
alternative hypothesis: true rho is not equal to 0
sample estimates:
       rho 
-0.2278242 

Warning message:
In cor.test.default(epi$withdrawaltime, epi$Prov2rutinCRP, use =
"complete.obs",  :
  Cannot compute exact p-value with ties
> text(x=160, y=1.5, "N/S", pos=3, cex=2)
> 
> ##Calculation of liver tests
> boxplot(main="Bilirubin",epi$Prov1rutinBIL,epi$Prov2rutinBIL)
> mean(epi$Prov1rutinBIL, na.rm=TRUE)
[1] 14.05263
> sd(epi$Prov1rutinBIL, na.rm=TRUE)
[1] 21.78016
> mean(epi$Prov2rutinBIL, na.rm=TRUE)
[1] 10.51613
> sd(epi$Prov2rutinBIL, na.rm=TRUE)
[1] 16.47801
> t.test(epi$Prov1rutinBIL,epi$Prov2rutinBIL,alternative =
c("two.sided"),paired = TRUE)

	Paired t-test

data:  epi$Prov1rutinBIL and epi$Prov2rutinBIL
t = 2.5067, df = 30, p-value = 0.01783
alternative hypothesis: true difference in means is not equal to 0
95 percent confidence interval:
 0.6634288 6.4978615
sample estimates:
mean of the differences 
               3.580645 

> wilcox.test(epi$Prov1rutinBIL,epi$Prov2rutinBIL,alternative =
c("two.sided"),paired = TRUE)

	Wilcoxon signed rank test with continuity correction

data:  epi$Prov1rutinBIL and epi$Prov2rutinBIL
```

```
V = 258.5, p-value = 0.009943
alternative hypothesis: true location shift is not equal to 0

Warning messages:
1: In wilcox.test.default(epi$Prov1rutinBIL, epi$Prov2rutinBIL, alternative =
c("two.sided"),  :
  cannot compute exact p-value with ties
2: In wilcox.test.default(epi$Prov1rutinBIL, epi$Prov2rutinBIL, alternative =
c("two.sided"),  :
  cannot compute exact p-value with zeroes
>
> boxplot(main="ALP",epi$Prov1rutinALP,epi$Prov2rutinALP)
> mean(epi$Prov1rutinALP, na.rm=TRUE)
[1] 1.64
> sd(epi$Prov1rutinALP, na.rm=TRUE)
[1] 1.045347
> mean(epi$Prov2rutinALP, na.rm=TRUE)
[1] 2.297813
> sd(epi$Prov2rutinALP, na.rm=TRUE)
[1] 1.302295
> t.test(epi$Prov1rutinALP,epi$Prov2rutinALP,alternative =
c("two.sided"),paired = TRUE)

	Paired t-test

data:  epi$Prov1rutinALP and epi$Prov2rutinALP
t = -2.6637, df = 31, p-value = 0.01215
alternative hypothesis: true difference in means is not equal to 0
95 percent confidence interval:
 -1.1410564 -0.1514436
sample estimates:
mean of the differences
               -0.64625

> wilcox.test(epi$Prov1rutinALP,epi$Prov2rutinALP,alternative =
c("two.sided"),paired = TRUE)

	Wilcoxon signed rank test with continuity correction

data:  epi$Prov1rutinALP and epi$Prov2rutinALP
V = 116, p-value = 0.009961
alternative hypothesis: true location shift is not equal to 0

Warning messages:
1: In wilcox.test.default(epi$Prov1rutinALP, epi$Prov2rutinALP, alternative =
c("two.sided"),  :
  cannot compute exact p-value with ties
2: In wilcox.test.default(epi$Prov1rutinALP, epi$Prov2rutinALP, alternative =
c("two.sided"),  :
  cannot compute exact p-value with zeroes
>
>
> ###Diagrams for advanced tests 140520
>
>
> ###Boxplot coagulation factors
```

```
> dev.new(width=3)
> boxplot(main="Fibrinogen",epi$FIBpreop,epi$FIBpostop,horizontal=FALSE,
na.rm=TRUE, axes=TRUE,cex.main=1.65,cex.axis=1.5,cex.lab=1.45, ylab="Fibrinogen
concentration (g/L)", boxwex=1, col=c(24,0), lty=1,ylim=c(0,8), xlim=c(0,2.5))
> arrows(0.25,2,,4,code=0,lwd=2,lty=3)
> my.legend.size <-legend(0.2,1, c("Preoperative","Postoperative"), cex=
1,fill=c(24,0),plot=FALSE)
> my.legend.size
$rect
$rect$w
[1] 2.322804

$rect$h
[1] 1.004651

$rect$left
[1] 0.2

$rect$top
[1] 1


$text
$text$x
[1] 0.8443182 0.8443182

$text$y
[1] 0.6651163 0.3302326


> legend((2.5-my.legend.size$rect$w)/2,1, c("Preoperative","Postoperative"),
cex= 1,fill=c(24,0))
> mean(epi$FIBpreop, na.rm=TRUE)
[1] 3.159189
> sd(epi$FIBpreop, na.rm=TRUE)
[1] 0.941389
> mean(epi$FIBpostop, na.rm=TRUE)
[1] 5.84129
> sd(epi$FIBpostop, na.rm=TRUE)
[1] 1.08464
>
>
> #hist(epi$FIBpreop)
> #hist(epi$FIBpostop)
>
> t.test(epi$FIBpreop,epi$FIBpostop,alternative = c("two.sided"),paired = TRUE)

	Paired t-test

data:  epi$FIBpreop and epi$FIBpostop
t = -11.345, df = 30, p-value = 2.239e-12
alternative hypothesis: true difference in means is not equal to 0
95 percent confidence interval:
 -3.081371 -2.141210
sample estimates:
mean of the differences
```

```
               -2.61129

> wilcox.test(epi$FIBpreop,epi$FIBpostop,alternative = c("two.sided"),paired =
TRUE)

    Wilcoxon signed rank test with continuity correction

data:  epi$FIBpreop and epi$FIBpostop
V = 3, p-value = 1.656e-06
alternative hypothesis: true location shift is not equal to 0

Warning message:
In wilcox.test.default(epi$FIBpreop, epi$FIBpostop, alternative =
c("two.sided"),  :
  cannot compute exact p-value with ties
> x<-1
> y<-1.5
> arrows(x,y,x+1,y, length=0.05, angle=90, code=1)
> arrows(x,y,x+1,y, length=0.05, angle=90, code=2)
> text(x+0.5,y-0.6,"**",pos=3, cex=1.4)
>
>
> boxplot(epi$F2preop,epi$F2postop,-100, epi$F7preop,epi$F7postop,-100, epi
$F9preop,epi$F9postop,-100, epi$F10preop,epi$F10postop,-100, epi$F11preop,epi
$F11postop,-100, epi$F12preop,epi$F12postop,-100, epi$F13preop,epi$F13postop,
horizontal=FALSE, na.rm=TRUE, axes=TRUE, main="Pre- and postoperative
concentrations of coagulation factors",cex.main=1.65,cex.axis=1.5,cex.lab=1.45,
ylab="Plasma concentration (kIE/L)", boxwex=1,
col=c(24,0,0,24,0,0,24,0,0,24,0,0,24,0,0,24,0,0,24,0,0,24,0,0),
lty=1,ylim=c(0.3,2.9),
names=c("F","2","","F","7","","F","9","","F","10","","F","11","","F","12","","F
","13"))
> legend(0,2.8, c("Preoperative","Postoperative"), cex= 1,fill=c(24,0))
> legend(5.7,5,lty=3,lwd=2,c("Reference range"))
> arrows(0.4,0.7,,1.5,code=0,lwd=2,lty=3)
> arrows(3.4,0.6,,1.6,code=0,lwd=2,lty=3)
> arrows(6.4,0.7,,1.3,code=0,lwd=2,lty=3)
> arrows(9.4,0.7,,1.52,code=0,lwd=2,lty=3)
> arrows(12.4,0.6,,1.3,code=0,lwd=2,lty=3)
> arrows(15.4,1.07,,1.5,code=0,lwd=2,lty=3)
> arrows(18.4,0.7,,1.4,code=0,lwd=2,lty=3)
>
>
>
> #hist(epi$F2preop)
> mean(epi$F2preop, na.rm=TRUE)
[1] 1.0395
> sd(epi$F2preop, na.rm=TRUE)
[1] 0.1941889
>
> #hist(epi$F2postop)
> mean(epi$F2postop, na.rm=TRUE)
[1] 1.038968
> sd(epi$F2postop, na.rm=TRUE)
[1] 0.2792906
>
```

```
> t.test(epi$F2preop, epi$F2postop,alternative = c("two.sided"),paired = TRUE)

	Paired t-test

data:  epi$F2preop and epi$F2postop
t = -0.57852, df = 30, p-value = 0.5672
alternative hypothesis: true difference in means is not equal to 0
95 percent confidence interval:
 -0.13751286  0.07680318
sample estimates:
mean of the differences 
            -0.03035484 

> wilcox.test(epi$F2preop, epi$F2postop,alternative = c("two.sided"),paired = 
TRUE)

	Wilcoxon signed rank test

data:  epi$F2preop and epi$F2postop
V = 224, p-value = 0.6496
alternative hypothesis: true location shift is not equal to 0

> 
> hist(epi$F7preop)
> mean(epi$F7preop, na.rm=TRUE)
[1] 1.101763
> sd(epi$F7preop, na.rm=TRUE)
[1] 0.2338181
> 
> #hist(epi$F7postop)
> mean(epi$F7postop, na.rm=TRUE)
[1] 1.003161
> sd(epi$F7postop, na.rm=TRUE)
[1] 0.2336632
> 
> t.test(epi$F7preop, epi$F7postop,alternative = c("two.sided"),paired = TRUE)

	Paired t-test

data:  epi$F7preop and epi$F7postop
t = 1.8705, df = 30, p-value = 0.0712
alternative hypothesis: true difference in means is not equal to 0
95 percent confidence interval:
 -0.00623686  0.14204331
sample estimates:
mean of the differences 
             0.06790323 

> wilcox.test(epi$F7preop, epi$F7postop,alternative = c("two.sided"),paired = 
TRUE)

	Wilcoxon signed rank test

data:  epi$F7preop and epi$F7postop
V = 323, p-value = 0.1461
alternative hypothesis: true location shift is not equal to 0
```

```
> #N/S
> x<-1
> y<-85
> arrows(x,y,x+1,y, length=0.05, angle=90, code=1)
> arrows(x,y,x+1,y, length=0.05, angle=90, code=2)
> text(x+0.5,y-3,"**",pos=3, cex=1.4)
>
> #hist(epi$F9preop)
> mean(epi$F9preop, na.rm=TRUE)
[1] 1.075526
> sd(epi$F9preop, na.rm=TRUE)
[1] 0.258687
>
> #hist(epi$F9postop)
> mean(epi$F9postop, na.rm=TRUE)
[1] 1.522516
> sd(epi$F9postop, na.rm=TRUE)
[1] 0.3873196
>
> t.test(epi$F9preop, epi$F9postop,alternative = c("two.sided"),paired = TRUE)

    Paired t-test

data:  epi$F9preop and epi$F9postop
t = -6.5032, df = 30, p-value = 3.448e-07
alternative hypothesis: true difference in means is not equal to 0
95 percent confidence interval:
 -0.6060707 -0.3163809
sample estimates:
mean of the differences
             -0.4612258

> wilcox.test(epi$F9preop, epi$F9postop,alternative = c("two.sided"),paired =
TRUE)

    Wilcoxon signed rank test

data:  epi$F9preop and epi$F9postop
V = 22, p-value = 4.992e-07
alternative hypothesis: true location shift is not equal to 0

> x<-7
> y<-2.7
> arrows(x,y,x+1,y, length=0.05, angle=90, code=1)
> arrows(x,y,x+1,y, length=0.05, angle=90, code=2)
> text(x+0.5,y-0.1,"**",pos=3, cex=1.4)
>
> #hist(epi$F10preop)
> mean(epi$F10preop, na.rm=TRUE)
[1] 0.8509737
> sd(epi$F10preop, na.rm=TRUE)
[1] 0.1776195
>
> #hist(epi$F10postop)
> mean(epi$F10postop, na.rm=TRUE)
```

```
[1] 0.9247742
> sd(epi$F10postop, na.rm=TRUE)
[1] 0.2636621
>
> t.test(epi$F10preop, epi$F10postop,alternative = c("two.sided"),paired =
TRUE)

	Paired t-test

data:  epi$F10preop and epi$F10postop
t = -2.0785, df = 30, p-value = 0.04631
alternative hypothesis: true difference in means is not equal to 0
95 percent confidence interval:
 -0.163017255 -0.001434358
sample estimates:
mean of the differences
            -0.08222581

> wilcox.test(epi$F10preop, epi$F10postop,alternative = c("two.sided"),paired =
TRUE)

	Wilcoxon signed rank test with continuity correction

data:  epi$F10preop and epi$F10postop
V = 146, p-value = 0.04669
alternative hypothesis: true location shift is not equal to 0

Warning message:
In wilcox.test.default(epi$F10preop, epi$F10postop, alternative =
c("two.sided"),  :
  cannot compute exact p-value with ties
> x<-10
> y<-2.7
> arrows(x,y,x+1,y, length=0.05, angle=90, code=1)
> arrows(x,y,x+1,y, length=0.05, angle=90, code=2)
> text(x+0.5,y-0.1,"*",pos=3, cex=1.4)
>
> #hist(epi$F11preop)
> mean(epi$F11preop, na.rm=TRUE)
[1] 1.023395
> sd(epi$F11preop, na.rm=TRUE)
[1] 0.2127542
>
> #hist(epi$F11postop)
> mean(epi$F11postop, na.rm=TRUE)
[1] 1.063677
> sd(epi$F11postop, na.rm=TRUE)
[1] 0.3265792
>
> t.test(epi$F11preop, epi$F11postop,alternative = c("two.sided"),paired =
TRUE)

	Paired t-test

data:  epi$F11preop and epi$F11postop
t = -1.2461, df = 30, p-value = 0.2224
```

```
alternative hypothesis: true difference in means is not equal to 0
95 percent confidence interval:
 -0.1531418  0.0370773
sample estimates:
mean of the differences
            -0.05803226

> wilcox.test(epi$F11preop, epi$F11postop,alternative = c("two.sided"),paired =
TRUE)

    Wilcoxon signed rank test with continuity correction

data:  epi$F11preop and epi$F11postop
V = 189.5, p-value = 0.2557
alternative hypothesis: true location shift is not equal to 0

Warning message:
In wilcox.test.default(epi$F11preop, epi$F11postop, alternative =
c("two.sided"),  :
  cannot compute exact p-value with ties
> #N/S
> x<-10
> y<-2.7
> arrows(x,y,x+1,y, length=0.05, angle=90, code=1)
> arrows(x,y,x+1,y, length=0.05, angle=90, code=2)
> text(x+0.5,y-0.1,"*",pos=3, cex=1.4)
>
>
> #hist(epi$F12preop)
> mean(epi$F12preop, na.rm=TRUE)
[1] 0.9438158
> sd(epi$F12preop, na.rm=TRUE)
[1] 0.2528337
>
> #hist(epi$F12postop)
> mean(epi$F12postop, na.rm=TRUE)
[1] 0.7950968
> sd(epi$F12postop, na.rm=TRUE)
[1] 0.3280546
>
> t.test(epi$F12preop, epi$F12postop,alternative = c("two.sided"),paired =
TRUE)

    Paired t-test

data:  epi$F12preop and epi$F12postop
t = 5.3943, df = 30, p-value = 7.652e-06
alternative hypothesis: true difference in means is not equal to 0
95 percent confidence interval:
 0.1001053 0.2220882
sample estimates:
mean of the differences
              0.1610968

> wilcox.test(epi$F12preop, epi$F12postop,alternative = c("two.sided"),paired =
TRUE)
```

```
	Wilcoxon signed rank test with continuity correction

data:  epi$F12preop and epi$F12postop
V = 454, p-value = 5.645e-05
alternative hypothesis: true location shift is not equal to 0

Warning message:
In wilcox.test.default(epi$F12preop, epi$F12postop, alternative =
c("two.sided"),  :
  cannot compute exact p-value with ties
> x<-16
> y<-2.7
> arrows(x,y,x+1,y, length=0.05, angle=90, code=1)
> arrows(x,y,x+1,y, length=0.05, angle=90, code=2)
> text(x+0.5,y-0.1,"**",pos=3, cex=1.4)
>
> #hist(epi$F13preop)
> mean(epi$F13preop, na.rm=TRUE)
[1] 1.203579
> sd(epi$F13preop, na.rm=TRUE)
[1] 0.3481401
>
> #hist(epi$F13postop)
> mean(epi$F13postop, na.rm=TRUE)
[1] 0.7787742
> sd(epi$F13postop, na.rm=TRUE)
[1] 0.2205076
>
> t.test(epi$F13preop, epi$F13postop,alternative = c("two.sided"),paired =
TRUE)

	Paired t-test

data:  epi$F13preop and epi$F13postop
t = 8.4888, df = 30, p-value = 1.794e-09
alternative hypothesis: true difference in means is not equal to 0
95 percent confidence interval:
 0.2953388 0.4824676
sample estimates:
mean of the differences
              0.3889032

> wilcox.test(epi$F13preop, epi$F13postop,alternative = c("two.sided"),paired =
TRUE)

	Wilcoxon signed rank test

data:  epi$F13preop and epi$F13postop
V = 496, p-value = 9.313e-10
alternative hypothesis: true location shift is not equal to 0

> x<-19
> y<-2.7
> arrows(x,y,x+1,y, length=0.05, angle=90, code=1)
> arrows(x,y,x+1,y, length=0.05, angle=90, code=2)
```

```
> text(x+0.5,y-0.1,"**",pos=3, cex=1.4)
>
>
> mean(epi$Prov2rutinCRP,na.rm=TRUE)
[1] 91.96667
> sd(epi$Prov2rutinCRP,na.rm=TRUE)
[1] 59.82762
> hist(epi$Prov2rutinCRP)
>
> mean(epi$Prov1rutinPK,na.rm=TRUE)
[1] 1.029412
> sd(epi$Prov1rutinPK,na.rm=TRUE)
[1] 0.08714117
> hist(epi$Prov1rutinPK)
>
> mean(epi$Prov2rutinPK,na.rm=TRUE)
[1] 1.168571
> sd(epi$Prov2rutinPK,na.rm=TRUE)
[1] 0.1529541
> hist(epi$Prov2rutinPK)
>
>
> mean(epi$Prov1rutinAPTT,na.rm=TRUE)
[1] 27
> sd(epi$Prov1rutinAPTT,na.rm=TRUE)
[1] 2.872281
> hist(epi$Prov1rutinAPTT)
>
> mean(epi$Prov2rutinAPTT,na.rm=TRUE)
[1] 29.6
> sd(epi$Prov2rutinAPTT,na.rm=TRUE)
[1] 4.447074
> hist(epi$Prov2rutinAPTT)
>
>
> mean(epi$Prov1rutinTPK,na.rm=TRUE)
[1] 213.1053
> sd(epi$Prov1rutinTPK,na.rm=TRUE)
[1] 72.11282
> hist(epi$Prov1rutinTPK)
>
> mean(epi$Prov2rutinTPK,na.rm=TRUE)
[1] 283.0645
> sd(epi$Prov2rutinTPK,na.rm=TRUE)
[1] 153.446
> hist(epi$Prov2rutinTPK)
>
>
> #Correlation between withdrawal time and TPK
> hist(epi$withdrawaltime)
>
> plot(epi$withdrawaltime, epi$Prov2rutinTPK, xlab="Day of epidural
withdrawal", ylab = "Platelet count (millions/mL)", abline(lm(epi
$Prov2rutinTPK~epi$withdrawaltime)),cex.main=1.3, cex.lab=1.4)
> cor.test(epi$withdrawaltime, epi$Prov2rutinTPK, use="complete.obs",
method="pearson")
```

```
	Pearson's product-moment correlation

data:  epi$withdrawaltime and epi$Prov2rutinTPK
t = 3.6065, df = 29, p-value = 0.001151
alternative hypothesis: true correlation is not equal to 0
95 percent confidence interval:
 0.2517544 0.7607874
sample estimates:
     cor 
0.556455 

> cor.test(epi$withdrawaltime, epi$Prov2rutinTPK, use="complete.obs",
method="spearman")

	Spearman's rank correlation rho

data:  epi$withdrawaltime and epi$Prov2rutinTPK
S = 2363, p-value = 0.002504
alternative hypothesis: true rho is not equal to 0
sample estimates:
      rho 
0.5235982 

Warning message:
In cor.test.default(epi$withdrawaltime, epi$Prov2rutinTPK, use =
"complete.obs",  :
  Cannot compute exact p-value with ties
> text(x=13, y=700, "P<0.01", pos=3, cex=2)
> text(x=13, y=640, "r=0.56", pos=3, cex=2)
> 
> ####SMALL DIAGRAMS FOR COMPOSITE PLOT
> 
> #Standard plot
> cor.test(epi$XPARAM, epi$YPARAM, use="complete.obs", method="pearson")
Error in cor.test.default(epi$XPARAM, epi$YPARAM, use = "complete.obs",  : 
  'x' must be a numeric vector
> cor.test(epi$XPARAM, epi$YPARAM, use="complete.obs", method="spearman")
Error in cor.test.default(epi$XPARAM, epi$YPARAM, use = "complete.obs",  : 
  'x' must be a numeric vector
> #RESULT:
> plot(epi$XPARAM, epi$YPARAM, xlab="", ylab = "", cex.axis=3, cex=2.5, pch=19,
axes=F)
Error in plot.window(...) : need finite 'xlim' values
In addition: Warning messages:
1: In min(x) : no non-missing arguments to min; returning Inf
2: In max(x) : no non-missing arguments to max; returning -Inf
3: In min(x) : no non-missing arguments to min; returning Inf
4: In max(x) : no non-missing arguments to max; returning -Inf
> axis(side=1,lwd=4,cex.axis=3, labels=NA)
> axis(side=1,lwd=0,cex.axis=3, line=1)
> axis(side=2,lwd=4,cex.axis=3, labels=NA)
> axis(side=2,lwd=0,cex.axis=3, line=0)
> 
> #INR vs EXTEM-MCF
> cor.test(epi$Prov2rutinPK, epi$Prov2extemMCF, use="complete.obs",
```

```
method="pearson")

	Pearson's product-moment correlation

data:  epi$Prov2rutinPK and epi$Prov2extemMCF
t = -1.4426, df = 31, p-value = 0.1592
alternative hypothesis: true correlation is not equal to 0
95 percent confidence interval:
 -0.5470235  0.1012061
sample estimates:
       cor
-0.2508175

> cor.test(epi$Prov2rutinPK, epi$Prov2extemMCF, use="complete.obs",
method="spearman")

	Spearman's rank correlation rho

data:  epi$Prov2rutinPK and epi$Prov2extemMCF
S = 6546.7, p-value = 0.6027
alternative hypothesis: true rho is not equal to 0
sample estimates:
        rho
-0.09402775

Warning message:
In cor.test.default(epi$Prov2rutinPK, epi$Prov2extemMCF, use =
"complete.obs",  :
  Cannot compute exact p-value with ties
> #RESULT:
> plot(epi$Prov2rutinPK, epi$Prov2extemMCF, xlab="", ylab = "", cex.axis=3,
cex=2.5, pch=19, axes=F)
> axis(side=1,lwd=4,cex.axis=3, labels=NA)
> axis(side=1,lwd=0,cex.axis=3, line=1)
> axis(side=2,lwd=4,cex.axis=3, labels=NA)
> axis(side=2,lwd=0,cex.axis=3, line=0)
>
> #INR vs EXTEM-CT
> cor.test(epi$Prov2rutinPK, epi$Prov2extemCT, use="complete.obs",
method="pearson")

	Pearson's product-moment correlation

data:  epi$Prov2rutinPK and epi$Prov2extemCT
t = 1.1177, df = 31, p-value = 0.2723
alternative hypothesis: true correlation is not equal to 0
95 percent confidence interval:
 -0.1571117  0.5059370
sample estimates:
      cor
0.1968129

> cor.test(epi$Prov2rutinPK, epi$Prov2extemCT, use="complete.obs",
method="spearman")

	Spearman's rank correlation rho
```

```
data:  epi$Prov2rutinPK and epi$Prov2extemCT
S = 5462.4, p-value = 0.6296
alternative hypothesis: true rho is not equal to 0
sample estimates:
       rho
0.08716727

Warning message:
In cor.test.default(epi$Prov2rutinPK, epi$Prov2extemCT, use =
"complete.obs",  :
  Cannot compute exact p-value with ties
> #RESULT:
> plot(epi$Prov2rutinPK, epi$Prov2extemCT, xlab="", ylab = "", cex.axis=3,
cex=2.5, pch=19, axes=F)
> axis(side=1,lwd=4,cex.axis=3, labels=NA)
> axis(side=1,lwd=0,cex.axis=3, line=1)
> axis(side=2,lwd=4,cex.axis=3, labels=NA)
> axis(side=2,lwd=0,cex.axis=3, line=0)
>
> #INR vs INTEM-CT
> cor.test(epi$Prov2rutinPK, epi$Prov2intemCT, use="complete.obs",
method="pearson")

	Pearson's product-moment correlation

data:  epi$Prov2rutinPK and epi$Prov2intemCT
t = -0.50255, df = 31, p-value = 0.6188
alternative hypothesis: true correlation is not equal to 0
95 percent confidence interval:
 -0.4202349  0.2614838
sample estimates:
        cor
-0.08989508

> cor.test(epi$Prov2rutinPK, epi$Prov2intemCT, use="complete.obs",
method="spearman")

	Spearman's rank correlation rho

data:  epi$Prov2rutinPK and epi$Prov2intemCT
S = 6241.9, p-value = 0.8118
alternative hypothesis: true rho is not equal to 0
sample estimates:
        rho
-0.04309158

Warning message:
In cor.test.default(epi$Prov2rutinPK, epi$Prov2intemCT, use =
"complete.obs",  :
  Cannot compute exact p-value with ties
> #RESULT:
> plot(epi$Prov2rutinPK, epi$Prov2intemCT, xlab="", ylab = "", cex.axis=3,
cex=2.5, pch=19, axes=F)
> axis(side=1,lwd=4,cex.axis=3, labels=NA)
> axis(side=1,lwd=0,cex.axis=3, line=1)
```

```
> axis(side=2,lwd=4,cex.axis=3, labels=NA)
> axis(side=2,lwd=0,cex.axis=3, line=0)
>
>
> #INR vs FIBTEM-MCF
> cor.test(epi$Prov2rutinPK, epi$Prov2fibtemMCF, use="complete.obs",
method="pearson")

	Pearson's product-moment correlation

data:  epi$Prov2rutinPK and epi$Prov2fibtemMCF
t = -0.99309, df = 31, p-value = 0.3284
alternative hypothesis: true correlation is not equal to 0
95 percent confidence interval:
 -0.4893990  0.1784758
sample estimates:
       cor
-0.1755922

> cor.test(epi$Prov2rutinPK, epi$Prov2fibtemMCF, use="complete.obs",
method="spearman")

	Spearman's rank correlation rho

data:  epi$Prov2rutinPK and epi$Prov2fibtemMCF
S = 6137.7, p-value = 0.8872
alternative hypothesis: true rho is not equal to 0
sample estimates:
        rho
-0.02568291

Warning message:
In cor.test.default(epi$Prov2rutinPK, epi$Prov2fibtemMCF, use =
"complete.obs",  :
  Cannot compute exact p-value with ties
> #RESULT:
> plot(epi$Prov2rutinPK, epi$Prov2fibemMCF, xlab="", ylab = "", cex.axis=3,
cex=2.5, pch=19, axes=F)
> axis(side=1,lwd=4,cex.axis=3, labels=NA)
> axis(side=1,lwd=0,cex.axis=3, line=1)
> axis(side=2,lwd=4,cex.axis=3, labels=NA)
> axis(side=2,lwd=0,cex.axis=3, line=0)
>
> #aPTT vs INTEM-CT
> cor.test(epi$Prov2rutinAPTT, epi$Prov2intemCT, use="complete.obs",
method="pearson")

	Pearson's product-moment correlation

data:  epi$Prov2rutinAPTT and epi$Prov2intemCT
t = -0.89483, df = 31, p-value = 0.3778
alternative hypothesis: true correlation is not equal to 0
95 percent confidence interval:
 -0.4760559  0.1952656
sample estimates:
       cor
```

```
-0.1586808

> cor.test(epi$Prov2rutinAPTT, epi$Prov2intemCT, use="complete.obs",
method="spearman")

    Spearman's rank correlation rho

data:  epi$Prov2rutinAPTT and epi$Prov2intemCT
S = 6583.8, p-value = 0.5789
alternative hypothesis: true rho is not equal to 0
sample estimates:
      rho
-0.100237

Warning message:
In cor.test.default(epi$Prov2rutinAPTT, epi$Prov2intemCT, use =
"complete.obs",  :
  Cannot compute exact p-value with ties
> #RESULT:
> plot(epi$Prov2rutinAPTT, epi$Prov2intemCT, xlab="", ylab = "", cex.axis=3,
cex=2.5, pch=19, axes=F)
> axis(side=1,lwd=4,cex.axis=3, labels=NA)
> axis(side=1,lwd=0,cex.axis=3, line=1)
> axis(side=2,lwd=4,cex.axis=3, labels=NA)
> axis(side=2,lwd=0,cex.axis=3, line=0)
>
>
>
> #aPTT vs EXTEM-MCF
> cor.test(epi$Prov2rutinAPTT, epi$Prov2extemMCF, use="complete.obs",
method="pearson")

    Pearson's product-moment correlation

data:  epi$Prov2rutinAPTT and epi$Prov2extemMCF
t = -0.16956, df = 31, p-value = 0.8665
alternative hypothesis: true correlation is not equal to 0
95 percent confidence interval:
 -0.3698837  0.3161730
sample estimates:
        cor
-0.03044012

> cor.test(epi$Prov2rutinAPTT, epi$Prov2extemMCF, use="complete.obs",
method="spearman")

    Spearman's rank correlation rho

data:  epi$Prov2rutinAPTT and epi$Prov2extemMCF
S = 5841.2, p-value = 0.8951
alternative hypothesis: true rho is not equal to 0
sample estimates:
       rho
0.02386784

Warning message:
```

```
In cor.test.default(epi$Prov2rutinAPTT, epi$Prov2extemMCF, use =
"complete.obs",  :
  Cannot compute exact p-value with ties
> #RESULT:
> plot(epi$Prov2rutinAPTT, epi$Prov2extemMCF, xlab="", ylab = "", cex.axis=3,
cex=2.5, pch=19, axes=F)
> axis(side=1,lwd=4,cex.axis=3, labels=NA)
> axis(side=1,lwd=0,cex.axis=3, line=1)
> axis(side=2,lwd=4,cex.axis=3, labels=NA)
> axis(side=2,lwd=0,cex.axis=3, line=0)
>
> #aPTT vs EXTEM-CT
> cor.test(epi$Prov2rutinAPTT, epi$Prov2extemCT, use="complete.obs",
method="pearson")

    Pearson's product-moment correlation

data:  epi$Prov2rutinAPTT and epi$Prov2extemCT
t = 0.96335, df = 31, p-value = 0.3428
alternative hypothesis: true correlation is not equal to 0
95 percent confidence interval:
 -0.1835627  0.4853891
sample estimates:
      cor
0.1704904

> cor.test(epi$Prov2rutinAPTT, epi$Prov2extemCT, use="complete.obs",
method="spearman")

    Spearman's rank correlation rho

data:  epi$Prov2rutinAPTT and epi$Prov2extemCT
S = 6474.8, p-value = 0.65
alternative hypothesis: true rho is not equal to 0
sample estimates:
        rho
-0.08202175

Warning message:
In cor.test.default(epi$Prov2rutinAPTT, epi$Prov2extemCT, use =
"complete.obs",  :
  Cannot compute exact p-value with ties
> #RESULT:
> plot(epi$Prov2rutinAPTT, epi$Prov2extemCT, xlab="", ylab = "", cex.axis=3,
cex=2.5, pch=19, axes=F)
> axis(side=1,lwd=4,cex.axis=3, labels=NA)
> axis(side=1,lwd=0,cex.axis=3, line=1)
> axis(side=2,lwd=4,cex.axis=3, labels=NA)
> axis(side=2,lwd=0,cex.axis=3, line=0)
>
> #aPTT vs FIBTEM-MCF
> cor.test(epi$Prov2rutinAPTT, epi$Prov2fibtemMCF, use="complete.obs",
method="pearson")

    Pearson's product-moment correlation
```

```
data:  epi$Prov2rutinAPTT and epi$Prov2fibtemMCF
t = -0.9046, df = 31, p-value = 0.3727
alternative hypothesis: true correlation is not equal to 0
95 percent confidence interval:
 -0.4773939  0.1935995
sample estimates:
       cor
-0.1603683

> cor.test(epi$Prov2rutinAPTT, epi$Prov2fibtemMCF, use="complete.obs",
method="spearman")

    Spearman's rank correlation rho

data:  epi$Prov2rutinAPTT and epi$Prov2fibtemMCF
S = 6003.7, p-value = 0.9855
alternative hypothesis: true rho is not equal to 0
sample estimates:
         rho
-0.003286168

Warning message:
In cor.test.default(epi$Prov2rutinAPTT, epi$Prov2fibtemMCF, use =
"complete.obs",  :
  Cannot compute exact p-value with ties
> #RESULT:
> plot(epi$Prov2rutinAPTT, epi$Prov2fibtemMCF, xlab="", ylab = "", cex.axis=3,
cex=2.5, pch=19, axes=F)
> axis(side=1,lwd=4,cex.axis=3, labels=NA)
> axis(side=1,lwd=0,cex.axis=3, line=1)
> axis(side=2,lwd=4,cex.axis=3, labels=NA)
> axis(side=2,lwd=0,cex.axis=3, line=0)
>
> #aPTT vs FIBTEM-CT
> cor.test(epi$Prov2rutinAPTT, epi$Prov2fibtemCT, use="complete.obs",
method="pearson")

    Pearson's product-moment correlation

data:  epi$Prov2rutinAPTT and epi$Prov2fibtemCT
t = 1.129, df = 31, p-value = 0.2676
alternative hypothesis: true correlation is not equal to 0
95 percent confidence interval:
 -0.1551655  0.5074197
sample estimates:
      cor
0.1987298

> cor.test(epi$Prov2rutinAPTT, epi$Prov2fibtemCT, use="complete.obs",
method="spearman")

    Spearman's rank correlation rho

data:  epi$Prov2rutinAPTT and epi$Prov2fibtemCT
S = 4797.7, p-value = 0.2687
alternative hypothesis: true rho is not equal to 0
```

```
sample estimates:
      rho
0.1982488

Warning message:
In cor.test.default(epi$Prov2rutinAPTT, epi$Prov2fibtemCT, use =
"complete.obs",  :
  Cannot compute exact p-value with ties
> #RESULT:
> plot(epi$Prov2rutinAPTT, epi$Prov2fibtemCT, xlab="", ylab = "", cex.axis=3,
cex=2.5, pch=19, axes=F)
> axis(side=1,lwd=4,cex.axis=3, labels=NA)
> axis(side=1,lwd=0,cex.axis=3, line=1)
> axis(side=2,lwd=4,cex.axis=3, labels=NA)
> axis(side=2,lwd=0,cex.axis=3, line=0)
>
> #Plc vs EXTEM-MCF
> cor.test(epi$Prov2rutinTPK, epi$Prov2extemMCF, use="complete.obs",
method="pearson")

    Pearson's product-moment correlation

data:  epi$Prov2rutinTPK and epi$Prov2extemMCF
t = 3.7841, df = 29, p-value = 0.0007166
alternative hypothesis: true correlation is not equal to 0
95 percent confidence interval:
 0.2770366 0.7720028
sample estimates:
      cor
0.5749378

> cor.test(epi$Prov2rutinTPK, epi$Prov2extemMCF, use="complete.obs",
method="spearman")

    Spearman's rank correlation rho

data:  epi$Prov2rutinTPK and epi$Prov2extemMCF
S = 1981.3, p-value = 0.0003543
alternative hypothesis: true rho is not equal to 0
sample estimates:
      rho
0.6005494

Warning message:
In cor.test.default(epi$Prov2rutinTPK, epi$Prov2extemMCF, use =
"complete.obs",  :
  Cannot compute exact p-value with ties
> #RESULT:
> plot(epi$Prov2rutinTPK, epi$Prov2extemMCF, xlab="", ylab = "", cex.axis=3,
cex=2.5, pch=19, axes=F,abline(lm(epi$Prov2extemMCF~ epi$Prov2rutinTPK),lwd=3))
> axis(side=1,lwd=4,cex.axis=3, labels=NA)
> axis(side=1,lwd=0,cex.axis=3, line=1)
> axis(side=2,lwd=4,cex.axis=3, labels=NA)
> axis(side=2,lwd=0,cex.axis=3, line=0)
> text(500, 55 , "r=0.57 **", pos=3, cex=7)
>
```

```
>
> #Plc vs EXTEM-CT
> cor.test(epi$Prov2rutinTPK, epi$Prov2extemCT, use="complete.obs",
method="pearson")

    Pearson's product-moment correlation

data:  epi$Prov2rutinTPK and epi$Prov2extemCT
t = -0.19277, df = 29, p-value = 0.8485
alternative hypothesis: true correlation is not equal to 0
95 percent confidence interval:
 -0.3852304  0.3226567
sample estimates:
        cor
-0.03577342

> cor.test(epi$Prov2rutinTPK, epi$Prov2extemCT, use="complete.obs",
method="spearman")

    Spearman's rank correlation rho

data:  epi$Prov2rutinTPK and epi$Prov2extemCT
S = 4601.2, p-value = 0.699
alternative hypothesis: true rho is not equal to 0
sample estimates:
       rho
0.07233799

Warning message:
In cor.test.default(epi$Prov2rutinTPK, epi$Prov2extemCT, use =
"complete.obs",  :
  Cannot compute exact p-value with ties
> #RESULT:
> plot(epi$Prov2rutinTPK, epi$Prov2extemCT, xlab="", ylab = "", cex.axis=3,
cex=2.5, pch=19, axes=F)
> axis(side=1,lwd=4,cex.axis=3, labels=NA)
> axis(side=1,lwd=0,cex.axis=3, line=1)
> axis(side=2,lwd=4,cex.axis=3, labels=NA)
> axis(side=2,lwd=0,cex.axis=3, line=0)
>
> #Plc vs INTEM-CT
> cor.test(epi$Prov2rutinTPK, epi$Prov2intemCT, use="complete.obs",
method="pearson")

    Pearson's product-moment correlation

data:  epi$Prov2rutinTPK and epi$Prov2intemCT
t = 0.4713, df = 29, p-value = 0.641
alternative hypothesis: true correlation is not equal to 0
95 percent confidence interval:
 -0.2756719  0.4282934
sample estimates:
       cor
0.08718459

> cor.test(epi$Prov2rutinTPK, epi$Prov2intemCT, use="complete.obs",
```

```
method="spearman")

	Spearman's rank correlation rho

data:  epi$Prov2rutinTPK and epi$Prov2intemCT
S = 5611.2, p-value = 0.4814
alternative hypothesis: true rho is not equal to 0
sample estimates:
       rho 
-0.1312897 

Warning message:
In cor.test.default(epi$Prov2rutinTPK, epi$Prov2intemCT, use =
"complete.obs",  :
  Cannot compute exact p-value with ties
> #RESULT:
> plot(epi$Prov2rutinTPK, epi$Prov2intemCT, xlab="", ylab = "", cex.axis=3,
cex=2.5, pch=19, axes=F)
> axis(side=1,lwd=4,cex.axis=3, labels=NA)
> axis(side=1,lwd=0,cex.axis=3, line=1)
> axis(side=2,lwd=4,cex.axis=3, labels=NA)
> axis(side=2,lwd=0,cex.axis=3, line=0)
> 
> #Plc vs FIBTEM-MCF
> cor.test(epi$Prov2rutinTPK, epi$Prov2fibtemMCF, use="complete.obs",
method="pearson")

	Pearson's product-moment correlation

data:  epi$Prov2rutinTPK and epi$Prov2fibtemMCF
t = 3.5516, df = 29, p-value = 0.001331
alternative hypothesis: true correlation is not equal to 0
95 percent confidence interval:
 0.2437729 0.7571824
sample estimates:
      cor 
0.5505567 

> cor.test(epi$Prov2rutinTPK, epi$Prov2fibtemMCF, use="complete.obs",
method="spearman")

	Spearman's rank correlation rho

data:  epi$Prov2rutinTPK and epi$Prov2fibtemMCF
S = 1826, p-value = 0.0001375
alternative hypothesis: true rho is not equal to 0
sample estimates:
      rho 
0.6318629 

Warning message:
In cor.test.default(epi$Prov2rutinTPK, epi$Prov2fibtemMCF, use =
"complete.obs",  :
  Cannot compute exact p-value with ties
> #RESULT:
> plot(epi$Prov2rutinTPK, epi$Prov2fibtemMCF, xlab="", ylab = "", cex.axis=3,
```

```
cex=2.5, pch=19, axes=F,abline(lm(epi$Prov2fibtemMCF~epi$Prov2rutinTPK),lwd=3))
> axis(side=1,lwd=4,cex.axis=3, labels=NA)
> axis(side=1,lwd=0,cex.axis=3, line=1)
> axis(side=2,lwd=4,cex.axis=3, labels=NA)
> axis(side=2,lwd=0,cex.axis=3, line=0)
> text(550, 20 , "r=0.55 **", cex=7)
>
>
> #DD vs EXTEM-MCF
> cor.test(epi$DDpostop, epi$Prov2extemMCF, use="complete.obs",
method="pearson")

	Pearson's product-moment correlation

data:  epi$DDpostop and epi$Prov2extemMCF
t = 1.1426, df = 29, p-value = 0.2626
alternative hypothesis: true correlation is not equal to 0
95 percent confidence interval:
 -0.1584429  0.5233974
sample estimates:
      cor 
0.2075496 

> cor.test(epi$DDpostop, epi$Prov2extemMCF, use="complete.obs",
method="spearman")

	Spearman's rank correlation rho

data:  epi$DDpostop and epi$Prov2extemMCF
S = 3875.7, p-value = 0.2374
alternative hypothesis: true rho is not equal to 0
sample estimates:
      rho 
0.2186031 

Warning message:
In cor.test.default(epi$DDpostop, epi$Prov2extemMCF, use = "complete.obs",  :
  Cannot compute exact p-value with ties
> #RESULT:
> plot(epi$DDpostop, epi$Prov2extemMCF, xlab="", ylab = "", cex.axis=3,
cex=2.5, pch=19, axes=F)
> axis(side=1,lwd=4,cex.axis=3, labels=NA)
> axis(side=1,lwd=0,cex.axis=3, line=1)
> axis(side=2,lwd=4,cex.axis=3, labels=NA)
> axis(side=2,lwd=0,cex.axis=3, line=0)
>
> #DD vs EXTEM-CT
> cor.test(epi$DDpostop, epi$Prov2extemCT, use="complete.obs",
method="pearson")

	Pearson's product-moment correlation

data:  epi$DDpostop and epi$Prov2extemCT
t = -2.5824, df = 29, p-value = 0.01513
alternative hypothesis: true correlation is not equal to 0
95 percent confidence interval:
```

```
 -0.6822053 -0.0921686
sample estimates:
       cor
-0.4323874

> cor.test(epi$DDpostop, epi$Prov2extemCT, use="complete.obs",
method="spearman")

	Spearman's rank correlation rho

data:  epi$DDpostop and epi$Prov2extemCT
S = 6410.6, p-value = 0.1104
alternative hypothesis: true rho is not equal to 0
sample estimates:
       rho
-0.2924667

Warning message:
In cor.test.default(epi$DDpostop, epi$Prov2extemCT, use = "complete.obs",  :
  Cannot compute exact p-value with ties
> #RESULT:
> plot(epi$DDpostop, epi$Prov2extemCT, xlab="", ylab = "", cex.axis=3, cex=2.5,
pch=19, axes=F,abline(lm(epi$Prov2extemCT~ epi$DDpostop),lwd=3,lty=2))
> axis(side=1,lwd=4,cex.axis=3, labels=NA)
> axis(side=1,lwd=0,cex.axis=3, line=1)
> axis(side=2,lwd=4,cex.axis=3, labels=NA)
> axis(side=2,lwd=0,cex.axis=3, line=0)
> text(3.7,70,"r=-0.43*",cex=7)
> #Note: given the outler with a really high D-Dimer, and that the non-
parametric test does not give significance while the parametric one does, I
have counted this as a 'non-significant' result. /OT
>
> #DD vs INTEM-CT
> cor.test(epi$DDpostop, epi$Prov2intemCT, use="complete.obs",
method="pearson")

	Pearson's product-moment correlation

data:  epi$DDpostop and epi$Prov2intemCT
t = -0.17456, df = 29, p-value = 0.8626
alternative hypothesis: true correlation is not equal to 0
95 percent confidence interval:
 -0.3823491  0.3256805
sample estimates:
        cor
-0.03239841

> cor.test(epi$DDpostop, epi$Prov2intemCT, use="complete.obs",
method="spearman")

	Spearman's rank correlation rho

data:  epi$DDpostop and epi$Prov2intemCT
S = 5913.5, p-value = 0.3002
alternative hypothesis: true rho is not equal to 0
sample estimates:
```

```
      rho 
-0.192234 

Warning message:
In cor.test.default(epi$DDpostop, epi$Prov2intemCT, use = "complete.obs",  :
  Cannot compute exact p-value with ties
> #RESULT:
> plot(epi$DDpostop, epi$Prov2intemCT, xlab="", ylab = "", cex.axis=3, cex=2.5, 
pch=19, axes=F)
> axis(side=1,lwd=4,cex.axis=3, labels=NA)
> axis(side=1,lwd=0,cex.axis=3, line=1)
> axis(side=2,lwd=4,cex.axis=3, labels=NA)
> axis(side=2,lwd=0,cex.axis=3, line=0)
> 
> #DD vs FIBTEM-MCF
> cor.test(epi$DDpostop, epi$Prov2fibtemMCF, use="complete.obs", 
method="pearson")

	Pearson's product-moment correlation

data:  epi$DDpostop and epi$Prov2fibtemMCF
t = 3.1564, df = 29, p-value = 0.003709
alternative hypothesis: true correlation is not equal to 0
95 percent confidence interval:
 0.1843580 0.7293265
sample estimates:
      cor 
0.5056653 

> cor.test(epi$DDpostop, epi$Prov2fibtemMCF, use="complete.obs", 
method="spearman")

	Spearman's rank correlation rho

data:  epi$DDpostop and epi$Prov2fibtemMCF
S = 1948.5, p-value = 0.0002926
alternative hypothesis: true rho is not equal to 0
sample estimates:
      rho 
0.6071597 

Warning message:
In cor.test.default(epi$DDpostop, epi$Prov2fibtemMCF, use = "complete.obs",  :
  Cannot compute exact p-value with ties
> #RESULT:
> plot(epi$DDpostop, epi$Prov2fibtemMCF, xlab="", ylab = "", cex.axis=3, 
cex=2.5, pch=19, axes=F,abline(lm(epi$Prov2fibtemMCF~ epi$DDpostop),lwd=3))
> axis(side=1,lwd=4,cex.axis=3, labels=NA)
> axis(side=1,lwd=0,cex.axis=3, line=1)
> axis(side=2,lwd=4,cex.axis=3, labels=NA)
> axis(side=2,lwd=0,cex.axis=3, line=0)
> text(3.5, 22 , "r=0.51**", cex=7, pos=1)
> 
> #Fibrinogen vs EXTEM-MCF
> cor.test(epi$FIBpostop, epi$Prov2extemMCF, use="complete.obs", 
method="pearson")
```

```
    Pearson's product-moment correlation

data:  epi$FIBpostop and epi$Prov2extemMCF
t = 2.2847, df = 29, p-value = 0.02983
alternative hypothesis: true correlation is not equal to 0
95 percent confidence interval:
 0.04203983 0.65434600
sample estimates:
     cor
0.390562

> cor.test(epi$FIBpostop, epi$Prov2extemMCF, use="complete.obs",
method="spearman")

    Spearman's rank correlation rho

data:  epi$FIBpostop and epi$Prov2extemMCF
S = 3458.5, p-value = 0.09785
alternative hypothesis: true rho is not equal to 0
sample estimates:
      rho
0.3027318

Warning message:
In cor.test.default(epi$FIBpostop, epi$Prov2extemMCF, use = "complete.obs",  :
  Cannot compute exact p-value with ties
> #RESULT:
> plot(epi$FIBpostop, epi$Prov2extemMCF, xlab="", ylab = "", cex.axis=3,
cex=2.5, pch=19, axes=F,abline(lm(epi$Prov2extemMCF~ epi
$FIBpostop),lwd=3,lty=2))
> axis(side=1,lwd=4,cex.axis=3, labels=NA)
> axis(side=1,lwd=0,cex.axis=3, line=1)
> axis(side=2,lwd=4,cex.axis=3, labels=NA)
> axis(side=2,lwd=0,cex.axis=3, line=0)
> epi$Prov2extemMCF
 [1] 71 76 74 75 77 73 73 73 75 68 77 74 66 70 NA 63 69 80 68 NA 71 64 72 65 63
[26] NA 60 54 NA NA 80 76 77 75 77 73 79 77
> text(6.4,58,"r=0.39*",cex=8)
> #NB given that the non-parametric test is non-significant, parametric sig.
>
> #Fibrinogen vs EXTEM-CT
> cor.test(epi$FIBpostop, epi$Prov2extemCT, use="complete.obs",
method="pearson")

    Pearson's product-moment correlation

data:  epi$FIBpostop and epi$Prov2extemCT
t = -1.6132, df = 29, p-value = 0.1175
alternative hypothesis: true correlation is not equal to 0
95 percent confidence interval:
 -0.58211669  0.07499732
sample estimates:
       cor
-0.2869689
```

```
> cor.test(epi$FIBpostop, epi$Prov2extemCT, use="complete.obs",
method="spearman")

	Spearman's rank correlation rho

data:  epi$FIBpostop and epi$Prov2extemCT
S = 7078.1, p-value = 0.01658
alternative hypothesis: true rho is not equal to 0
sample estimates:
       rho
-0.4270374

Warning message:
In cor.test.default(epi$FIBpostop, epi$Prov2extemCT, use = "complete.obs",  :
  Cannot compute exact p-value with ties
> #RESULT:
> plot(epi$FIBpostop, epi$Prov2extemCT, xlab="", ylab = "", cex.axis=3,
cex=2.5, pch=19, axes=F,abline(lm(epi$Prov2extemCT~ epi
$FIBpostop),lwd=3,lty=2))
> axis(side=1,lwd=4,cex.axis=3, labels=NA)
> axis(side=1,lwd=0,cex.axis=3, line=1)
> axis(side=2,lwd=4,cex.axis=3, labels=NA)
> axis(side=2,lwd=0,cex.axis=3, line=0)
> text(5.5,30,"rho=-0.42*",cex=6)
> #NB given that the non-parametric but not the parametric test is significant,
I have not counted this as significant.
>
> #Fibrinogen vs INTEM-CT
> cor.test(epi$FIBpostop, epi$Prov2intemCT, use="complete.obs",
method="pearson")

	Pearson's product-moment correlation

data:  epi$FIBpostop and epi$Prov2intemCT
t = -0.13747, df = 29, p-value = 0.8916
alternative hypothesis: true correlation is not equal to 0
95 percent confidence interval:
 -0.3764550  0.3318216
sample estimates:
        cor
-0.02551897

> cor.test(epi$FIBpostop, epi$Prov2intemCT, use="complete.obs",
method="spearman")

	Spearman's rank correlation rho

data:  epi$FIBpostop and epi$Prov2intemCT
S = 4353.6, p-value = 0.5124
alternative hypothesis: true rho is not equal to 0
sample estimates:
      rho
0.1222554

Warning message:
In cor.test.default(epi$FIBpostop, epi$Prov2intemCT, use = "complete.obs",  :
```

```
  Cannot compute exact p-value with ties
> #RESULT:
> plot(epi$FIBpostop, epi$Prov2intemCT, xlab="", ylab = "", cex.axis=3,
cex=2.5, pch=19, axes=F)
> axis(side=1,lwd=4,cex.axis=3, labels=NA)
> axis(side=1,lwd=0,cex.axis=3, line=1)
> axis(side=2,lwd=4,cex.axis=3, labels=NA)
> axis(side=2,lwd=0,cex.axis=3, line=0)
>
> #Fibrinogen vs FIBTEM-MCF
> cor.test(epi$FIBpostop, epi$fibtemMCF, use="complete.obs", method="pearson")
Error in cor.test.default(epi$FIBpostop, epi$fibtemMCF, use =
"complete.obs",  :
  'x' and 'y' must have the same length
> cor.test(epi$FIBpostop, epi$fibtemMCF, use="complete.obs", method="spearman")
Error in cor.test.default(epi$FIBpostop, epi$fibtemMCF, use =
"complete.obs",  :
  'x' and 'y' must have the same length
> #RESULT:
> plot(epi$FIBpostop, epi$fibtemMCF, xlab="", ylab = "", cex.axis=3, cex=2.5,
pch=19, axes=F)
> axis(side=1,lwd=4,cex.axis=3, labels=NA)
> axis(side=1,lwd=0,cex.axis=3, line=1)
> axis(side=2,lwd=4,cex.axis=3, labels=NA)
> axis(side=2,lwd=0,cex.axis=3, line=0)
>
> #F2 vs EXTEM-MCF
> cor.test(epi$F2postop, epi$Prov2extemMCF, use="complete.obs",
method="pearson")

    Pearson's product-moment correlation

data:  epi$F2postop and epi$Prov2extemMCF
t = 1.2043, df = 29, p-value = 0.2382
alternative hypothesis: true correlation is not equal to 0
95 percent confidence interval:
 -0.1475097  0.5314781
sample estimates:
      cor
0.2182373

> cor.test(epi$F2postop, epi$Prov2extemMCF, use="complete.obs",
method="spearman")

    Spearman's rank correlation rho

data:  epi$F2postop and epi$Prov2extemMCF
S = 4169.9, p-value = 0.392
alternative hypothesis: true rho is not equal to 0
sample estimates:
      rho
0.1592969

Warning message:
In cor.test.default(epi$F2postop, epi$Prov2extemMCF, use = "complete.obs",  :
  Cannot compute exact p-value with ties
```

```
> #RESULT:
> plot(epi$F2postop, epi$Prov2extemMCF, xlab="", ylab = "", cex.axis=3,
cex=2.5, pch=19, axes=F)
> axis(side=1,lwd=4,cex.axis=3, labels=NA)
> axis(side=1,lwd=0,cex.axis=3, line=1)
> axis(side=2,lwd=4,cex.axis=3, labels=NA)
> axis(side=2,lwd=0,cex.axis=3, line=0)
>
>
> #F2 vs EXTEM-CT
> cor.test(epi$F2postop, epi$Prov2extemCT, use="complete.obs",
method="pearson")

    Pearson's product-moment correlation

data:  epi$F2postop and epi$Prov2extemCT
t = -1.5235, df = 29, p-value = 0.1385
alternative hypothesis: true correlation is not equal to 0
95 percent confidence interval:
 -0.57143614  0.09089247
sample estimates:
       cor
-0.2722148

> cor.test(epi$F2postop, epi$Prov2extemCT, use="complete.obs",
method="spearman")

    Spearman's rank correlation rho

data:  epi$F2postop and epi$Prov2extemCT
S = 6559.9, p-value = 0.07676
alternative hypothesis: true rho is not equal to 0
sample estimates:
       rho
-0.3225616

Warning message:
In cor.test.default(epi$F2postop, epi$Prov2extemCT, use = "complete.obs",  :
  Cannot compute exact p-value with ties
> #RESULT:
> plot(epi$F2postop, epi$Prov2extemCT, xlab="", ylab = "", cex.axis=3, cex=2.5,
pch=19, axes=F)
> axis(side=1,lwd=4,cex.axis=3, labels=NA)
> axis(side=1,lwd=0,cex.axis=3, line=1)
> axis(side=2,lwd=4,cex.axis=3, labels=NA)
> axis(side=2,lwd=0,cex.axis=3, line=0)
>
> #F2 vs INTEM-CT
> cor.test(epi$F2postop, epi$Prov2intemCT, use="complete.obs",
method="pearson")

    Pearson's product-moment correlation

data:  epi$F2postop and epi$Prov2intemCT
t = -0.95783, df = 29, p-value = 0.3461
alternative hypothesis: true correlation is not equal to 0
```

```
95 percent confidence interval:
 -0.4985228  0.1910803
sample estimates:
       cor
-0.1751165

> cor.test(epi$F2postop, epi$Prov2intemCT, use="complete.obs",
method="spearman")

	Spearman's rank correlation rho

data:  epi$F2postop and epi$Prov2intemCT
S = 5693.4, p-value = 0.4273
alternative hypothesis: true rho is not equal to 0
sample estimates:
       rho
-0.1478568

Warning message:
In cor.test.default(epi$F2postop, epi$Prov2intemCT, use = "complete.obs",  :
  Cannot compute exact p-value with ties
> #RESULT:
> plot(epi$F2postop, epi$Prov2intemCT, xlab="", ylab = "", cex.axis=3, cex=2.5,
pch=19, axes=F)
> axis(side=1,lwd=4,cex.axis=3, labels=NA)
> axis(side=1,lwd=0,cex.axis=3, line=1)
> axis(side=2,lwd=4,cex.axis=3, labels=NA)
> axis(side=2,lwd=0,cex.axis=3, line=0)
>
> #F2 vs FIBTEM-MCF
> cor.test(epi$F2postop, epi$Prov2fibtemMCF, use="complete.obs",
method="pearson")

	Pearson's product-moment correlation

data:  epi$F2postop and epi$Prov2fibtemMCF
t = 2.7334, df = 29, p-value = 0.01057
alternative hypothesis: true correlation is not equal to 0
95 percent confidence interval:
 0.1170382 0.6954189
sample estimates:
      cor
0.4526081

> cor.test(epi$F2postop, epi$Prov2fibtemMCF, use="complete.obs",
method="spearman")

	Spearman's rank correlation rho

data:  epi$F2postop and epi$Prov2fibtemMCF
S = 3387.6, p-value = 0.08226
alternative hypothesis: true rho is not equal to 0
sample estimates:
      rho
0.3170251
```

```
Warning message:
In cor.test.default(epi$F2postop, epi$Prov2fibtemMCF, use = "complete.obs",  :
  Cannot compute exact p-value with ties
> #RESULT:
> plot(epi$F2postop, epi$Prov2fibtemMCF, xlab="", ylab = "", cex.axis=3,
cex=2.5, pch=19, axes=F,abline(lm(epi$Prov2fibtemMCF~ epi$F2postop),lwd=3,
lty=2))
> axis(side=1,lwd=4,cex.axis=3, labels=NA)
> axis(side=1,lwd=0,cex.axis=3, line=1)
> axis(side=2,lwd=4,cex.axis=3, labels=NA)
> axis(side=2,lwd=0,cex.axis=3, line=0)
> text(1.5,22,"r=0.45*",cex=7,pos=1)
>
>
>
> #F7 vs EXTEM-MCF
> cor.test(epi$F7postop, epi$Prov2extemMCF, use="complete.obs",
method="pearson")

	Pearson's product-moment correlation

data:  epi$F7postop and epi$Prov2extemMCF
t = 0.97461, df = 29, p-value = 0.3378
alternative hypothesis: true correlation is not equal to 0
95 percent confidence interval:
 -0.1881239  0.5008238
sample estimates:
      cor
0.1780875

> cor.test(epi$F7postop, epi$Prov2extemMCF, use="complete.obs",
method="spearman")

	Spearman's rank correlation rho

data:  epi$F7postop and epi$Prov2extemMCF
S = 4588.5, p-value = 0.6889
alternative hypothesis: true rho is not equal to 0
sample estimates:
       rho
0.07489179

Warning message:
In cor.test.default(epi$F7postop, epi$Prov2extemMCF, use = "complete.obs",  :
  Cannot compute exact p-value with ties
> #RESULT:
> plot(epi$F7postop, epi$Prov2extemMCF, xlab="", ylab = "", cex.axis=3,
cex=2.5, pch=19, axes=F)
> axis(side=1,lwd=4,cex.axis=3, labels=NA)
> axis(side=1,lwd=0,cex.axis=3, line=1)
> axis(side=2,lwd=4,cex.axis=3, labels=NA)
> axis(side=2,lwd=0,cex.axis=3, line=0)
>
>
> #F7 vs EXTEM-CT
> cor.test(epi$F7postop, epi$Prov2extemCT, use="complete.obs",
```

```
method="pearson")

    Pearson's product-moment correlation

data:  epi$F7postop and epi$Prov2extemCT
t = -1.6856, df = 29, p-value = 0.1026
alternative hypothesis: true correlation is not equal to 0
95 percent confidence interval:
 -0.59055327  0.06220115
sample estimates:
       cor 
-0.2987229 

> cor.test(epi$F7postop, epi$Prov2extemCT, use="complete.obs", 
method="spearman")

    Spearman's rank correlation rho

data:  epi$F7postop and epi$Prov2extemCT
S = 7005.7, p-value = 0.02113
alternative hypothesis: true rho is not equal to 0
sample estimates:
       rho 
-0.4124426 

Warning message:
In cor.test.default(epi$F7postop, epi$Prov2extemCT, use = "complete.obs",  :
  Cannot compute exact p-value with ties
> #RESULT:
> plot(epi$F7postop, epi$Prov2extemCT, xlab="", ylab = "", cex.axis=3, cex=2.5, 
pch=19, axes=F,abline(lm(epi$Prov2extemCT~ epi$F7postop),lwd=3,lty=2))
> axis(side=1,lwd=4,cex.axis=3, labels=NA)
> axis(side=1,lwd=0,cex.axis=3, line=1)
> axis(side=2,lwd=4,cex.axis=3, labels=NA)
> axis(side=2,lwd=0,cex.axis=3, line=0)
> text(1.1,70,"rho=-0.41",cex=6)
> 
> #F7 vs intem-CT
> cor.test(epi$F7postop, epi$Prov2intemCT, use="complete.obs", 
method="pearson")

    Pearson's product-moment correlation

data:  epi$F7postop and epi$Prov2intemCT
t = -1.1018, df = 29, p-value = 0.2796
alternative hypothesis: true correlation is not equal to 0
95 percent confidence interval:
 -0.5179922  0.1656640
sample estimates:
       cor 
-0.2004424 

> cor.test(epi$F7postop, epi$Prov2intemCT, use="complete.obs", 
method="spearman")

    Spearman's rank correlation rho
```

```
data:  epi$F7postop and epi$Prov2intemCT
S = 5766.4, p-value = 0.3822
alternative hypothesis: true rho is not equal to 0
sample estimates:
      rho
-0.162582

Warning message:
In cor.test.default(epi$F7postop, epi$Prov2intemCT, use = "complete.obs",  :
  Cannot compute exact p-value with ties
> #RESULT:
> plot(epi$F7postop, epi$Prov2intemCT, xlab="", ylab = "", cex.axis=3, cex=2.5,
pch=19, axes=F)
> axis(side=1,lwd=4,cex.axis=3, labels=NA)
> axis(side=1,lwd=0,cex.axis=3, line=1)
> axis(side=2,lwd=4,cex.axis=3, labels=NA)
> axis(side=2,lwd=0,cex.axis=3, line=0)
>
>
> #F7 vs fibtem-MCF
> cor.test(epi$F7postop, epi$Prov2fibtemMCF, use="complete.obs",
method="pearson")

    Pearson's product-moment correlation

data:  epi$F7postop and epi$Prov2fibtemMCF
t = 1.1696, df = 29, p-value = 0.2517
alternative hypothesis: true correlation is not equal to 0
95 percent confidence interval:
 -0.153646  0.526958
sample estimates:
      cor
0.2122496

> cor.test(epi$F7postop, epi$Prov2fibtemMCF, use="complete.obs",
method="spearman")

    Spearman's rank correlation rho

data:  epi$F7postop and epi$Prov2fibtemMCF
S = 4154.7, p-value = 0.3829
alternative hypothesis: true rho is not equal to 0
sample estimates:
      rho
0.1623541

Warning message:
In cor.test.default(epi$F7postop, epi$Prov2fibtemMCF, use = "complete.obs",  :
  Cannot compute exact p-value with ties
> #RESULT:
> plot(epi$F7postop, epi$Prov2fibtemMCF, xlab="", ylab = "", cex.axis=3,
cex=2.5, pch=19, axes=F)
> axis(side=1,lwd=4,cex.axis=3, labels=NA)
> axis(side=1,lwd=0,cex.axis=3, line=1)
> axis(side=2,lwd=4,cex.axis=3, labels=NA)
```

```
> axis(side=2,lwd=0,cex.axis=3, line=0)
>
>
> #F9 vs EXTEM-MCF
> cor.test(epi$F9postop, epi$Prov2extemMCF, use="complete.obs",
method="pearson")

    Pearson's product-moment correlation

data:  epi$F9postop and epi$Prov2extemMCF
t = 2.1374, df = 29, p-value = 0.04113
alternative hypothesis: true correlation is not equal to 0
95 percent confidence interval:
 0.0167591 0.6396360
sample estimates:
      cor
0.3689085

> cor.test(epi$F9postop, epi$Prov2extemMCF, use="complete.obs",
method="spearman")

    Spearman's rank correlation rho

data:  epi$F9postop and epi$Prov2extemMCF
S = 3572.5, p-value = 0.1275
alternative hypothesis: true rho is not equal to 0
sample estimates:
     rho
0.279731

Warning message:
In cor.test.default(epi$F9postop, epi$Prov2extemMCF, use = "complete.obs",  :
  Cannot compute exact p-value with ties
> #RESULT:
> plot(epi$F9postop, epi$Prov2extemMCF, xlab="", ylab = "", cex.axis=3,
cex=2.5, pch=19, axes=F,abline(lm(epi$Prov2extemMCF~ epi
$F9postop),lwd=3,lty=2))
> axis(side=1,lwd=4,cex.axis=3, labels=NA)
> axis(side=1,lwd=0,cex.axis=3, line=1)
> axis(side=2,lwd=4,cex.axis=3, labels=NA)
> axis(side=2,lwd=0,cex.axis=3, line=0)
> text(2,63,"r=0.37*",cex=7)
>
> #F9 vs EXTEM-CT
> cor.test(epi$F9postop, epi$Prov2extemCT, use="complete.obs",
method="pearson")

    Pearson's product-moment correlation

data:  epi$F9postop and epi$Prov2extemCT
t = -0.32209, df = 29, p-value = 0.7497
alternative hypothesis: true correlation is not equal to 0
95 percent confidence interval:
 -0.4054660  0.3010043
sample estimates:
        cor
```

```
-0.05970371

> cor.test(epi$F9postop, epi$Prov2extemCT, use="complete.obs",
method="spearman")

	Spearman's rank correlation rho

data:  epi$F9postop and epi$Prov2extemCT
S = 5615.2, p-value = 0.4787
alternative hypothesis: true rho is not equal to 0
sample estimates:
       rho
-0.1320947

Warning message:
In cor.test.default(epi$F9postop, epi$Prov2extemCT, use = "complete.obs",  :
  Cannot compute exact p-value with ties
> #RESULT:
> plot(epi$F9postop, epi$Prov2extemCT, xlab="", ylab = "", cex.axis=3, cex=2.5,
pch=19, axes=F)
> axis(side=1,lwd=4,cex.axis=3, labels=NA)
> axis(side=1,lwd=0,cex.axis=3, line=1)
> axis(side=2,lwd=4,cex.axis=3, labels=NA)
> axis(side=2,lwd=0,cex.axis=3, line=0)
>
> #F9 vs INTEM-CT
> cor.test(epi$F9postop, epi$Prov2intemCT, use="complete.obs",
method="pearson")

	Pearson's product-moment correlation

data:  epi$F9postop and epi$Prov2intemCT
t = -0.77183, df = 29, p-value = 0.4465
alternative hypothesis: true correlation is not equal to 0
95 percent confidence interval:
 -0.4724640  0.2237109
sample estimates:
       cor
-0.1418756

> cor.test(epi$F9postop, epi$Prov2intemCT, use="complete.obs",
method="spearman")

	Spearman's rank correlation rho

data:  epi$F9postop and epi$Prov2intemCT
S = 4669.9, p-value = 0.7546
alternative hypothesis: true rho is not equal to 0
sample estimates:
       rho
0.05849723

Warning message:
In cor.test.default(epi$F9postop, epi$Prov2intemCT, use = "complete.obs",  :
  Cannot compute exact p-value with ties
> #RESULT:
```

```
> plot(epi$F9postop, epi$Prov2intemCT, xlab="", ylab = "", cex.axis=3, cex=2.5,
pch=19, axes=F)
> axis(side=1,lwd=4,cex.axis=3, labels=NA)
> axis(side=1,lwd=0,cex.axis=3, line=1)
> axis(side=2,lwd=4,cex.axis=3, labels=NA)
> axis(side=2,lwd=0,cex.axis=3, line=0)
>
> #F9 vs FIBTEM-MCF
> cor.test(epi$F9postop, epi$Prov2intemMCF, use="complete.obs",
method="pearson")

    Pearson's product-moment correlation

data:  epi$F9postop and epi$Prov2intemMCF
t = -0.79539, df = 29, p-value = 0.4328
alternative hypothesis: true correlation is not equal to 0
95 percent confidence interval:
 -0.4758202  0.2195940
sample estimates:
       cor
-0.1461155

> cor.test(epi$F9postop, epi$Prov2intemMCF, use="complete.obs",
method="spearman")

    Spearman's rank correlation rho

data:  epi$F9postop and epi$Prov2intemMCF
S = 4941.9, p-value = 0.9845
alternative hypothesis: true rho is not equal to 0
sample estimates:
        rho
0.003645607

Warning message:
In cor.test.default(epi$F9postop, epi$Prov2intemMCF, use = "complete.obs",  :
  Cannot compute exact p-value with ties
> #RESULT:
> plot(epi$F9postop, epi$Prov2intemMCF, xlab="", ylab = "", cex.axis=3,
cex=2.5, pch=19, axes=F)
> axis(side=1,lwd=4,cex.axis=3, labels=NA)
> axis(side=1,lwd=0,cex.axis=3, line=1)
> axis(side=2,lwd=4,cex.axis=3, labels=NA)
> axis(side=2,lwd=0,cex.axis=3, line=0)
>
>
> #F10 vs EXTEM-MCF
> cor.test(epi$F10postop, epi$Prov2extemMCF, use="complete.obs",
method="pearson")

    Pearson's product-moment correlation

data:  epi$F10postop and epi$Prov2extemMCF
t = 2.4071, df = 29, p-value = 0.02267
alternative hypothesis: true correlation is not equal to 0
95 percent confidence interval:
```

```
 0.06282767 0.66610425
sample estimates:
      cor 
0.4080829 

> cor.test(epi$F10postop, epi$Prov2extemMCF, use="complete.obs", 
method="spearman")

	Spearman's rank correlation rho

data:  epi$F10postop and epi$Prov2extemMCF
S = 3348.5, p-value = 0.07453
alternative hypothesis: true rho is not equal to 0
sample estimates:
      rho 
0.3249012 

Warning message:
In cor.test.default(epi$F10postop, epi$Prov2extemMCF, use = "complete.obs",  :
  Cannot compute exact p-value with ties
> #RESULT:
> plot(epi$F10postop, epi$Prov2extemMCF, xlab="", ylab = "", cex.axis=3, 
cex=2.5, pch=19, axes=F,abline(lm(epi$Prov2extemMCF~ epi
$F10postop),lwd=3,lty=2))
> axis(side=1,lwd=4,cex.axis=3, labels=NA)
> axis(side=1,lwd=0,cex.axis=3, line=1)
> axis(side=2,lwd=4,cex.axis=3, labels=NA)
> axis(side=2,lwd=0,cex.axis=3, line=0)
> text(1.2,58,"r=0.41*",cex=7)
> 
> #F10 vs EXTEM-CT
> cor.test(epi$F10postop, epi$Prov2extemCT, use="complete.obs", 
method="pearson")

	Pearson's product-moment correlation

data:  epi$F10postop and epi$Prov2extemCT
t = -0.74502, df = 29, p-value = 0.4623
alternative hypothesis: true correlation is not equal to 0
95 percent confidence interval:
 -0.4686249  0.2283897
sample estimates:
       cor 
-0.1370408 

> cor.test(epi$F10postop, epi$Prov2extemCT, use="complete.obs", 
method="spearman")

	Spearman's rank correlation rho

data:  epi$F10postop and epi$Prov2extemCT
S = 6103.2, p-value = 0.2122
alternative hypothesis: true rho is not equal to 0
sample estimates:
       rho 
-0.2304821 
```

```
Warning message:
In cor.test.default(epi$F10postop, epi$Prov2extemCT, use = "complete.obs",  :
  Cannot compute exact p-value with ties
> #RESULT:
> plot(epi$F10postop, epi$Prov2extemCT, xlab="", ylab = "", cex.axis=3,
cex=2.5, pch=19, axes=F)
> axis(side=1,lwd=4,cex.axis=3, labels=NA)
> axis(side=1,lwd=0,cex.axis=3, line=1)
> axis(side=2,lwd=4,cex.axis=3, labels=NA)
> axis(side=2,lwd=0,cex.axis=3, line=0)
>
> #F10 vs INTEM-CT
> cor.test(epi$F10postop, epi$Prov2intemCT, use="complete.obs",
method="pearson")

    Pearson's product-moment correlation

data:  epi$F10postop and epi$Prov2intemCT
t = -0.32822, df = 29, p-value = 0.7451
alternative hypothesis: true correlation is not equal to 0
95 percent confidence interval:
 -0.4064152  0.2999704
sample estimates:
        cor
-0.06083604

> cor.test(epi$F10postop, epi$Prov2intemCT, use="complete.obs",
method="spearman")

    Spearman's rank correlation rho

data:  epi$F10postop and epi$Prov2intemCT
S = 4909, p-value = 0.9562
alternative hypothesis: true rho is not equal to 0
sample estimates:
       rho
0.01028848

Warning message:
In cor.test.default(epi$F10postop, epi$Prov2intemCT, use = "complete.obs",  :
  Cannot compute exact p-value with ties
> #RESULT:
> plot(epi$F10postop, epi$Prov2intemCT, xlab="", ylab = "", cex.axis=3,
cex=2.5, pch=19, axes=F)
> axis(side=1,lwd=4,cex.axis=3, labels=NA)
> axis(side=1,lwd=0,cex.axis=3, line=1)
> axis(side=2,lwd=4,cex.axis=3, labels=NA)
> axis(side=2,lwd=0,cex.axis=3, line=0)
>
> #F10 vs FIBTEM-MCF
> cor.test(epi$F10postop, epi$Prov2intemMCF, use="complete.obs",
method="pearson")

    Pearson's product-moment correlation
```

```
data:  epi$F10postop and epi$Prov2intemMCF
t = 0.59596, df = 29, p-value = 0.5558
alternative hypothesis: true correlation is not equal to 0
95 percent confidence interval:
 -0.2542543  0.4469167
sample estimates:
      cor
0.1099956

> cor.test(epi$F10postop, epi$Prov2intemMCF, use="complete.obs",
method="spearman")

	Spearman's rank correlation rho

data:  epi$F10postop and epi$Prov2intemMCF
S = 4177.4, p-value = 0.3966
alternative hypothesis: true rho is not equal to 0
sample estimates:
      rho
0.1577897

Warning message:
In cor.test.default(epi$F10postop, epi$Prov2intemMCF, use = "complete.obs",  :
  Cannot compute exact p-value with ties
> #RESULT:
> plot(epi$F10postop, epi$Prov2intemMCF, xlab="", ylab = "", cex.axis=3,
cex=2.5, pch=19, axes=F)
> axis(side=1,lwd=4,cex.axis=3, labels=NA)
> axis(side=1,lwd=0,cex.axis=3, line=1)
> axis(side=2,lwd=4,cex.axis=3, labels=NA)
> axis(side=2,lwd=0,cex.axis=3, line=0)
>
>
> #F11 vs EXTEM-MCF
> cor.test(epi$F11postop, epi$Prov2extemMCF, use="complete.obs",
method="pearson")

	Pearson's product-moment correlation

data:  epi$F11postop and epi$Prov2extemMCF
t = 1.7109, df = 29, p-value = 0.09778
alternative hypothesis: true correlation is not equal to 0
95 percent confidence interval:
 -0.05774782  0.59345628
sample estimates:
      cor
0.3027881

> cor.test(epi$F11postop, epi$Prov2extemMCF, use="complete.obs",
method="spearman")

	Spearman's rank correlation rho

data:  epi$F11postop and epi$Prov2extemMCF
S = 4471, p-value = 0.5978
alternative hypothesis: true rho is not equal to 0
```

```
sample estimates:
       rho
0.09858373

Warning message:
In cor.test.default(epi$F11postop, epi$Prov2extemMCF, use = "complete.obs",  :
  Cannot compute exact p-value with ties
> #RESULT:
> plot(epi$F11postop, epi$Prov2extemMCF, xlab="", ylab = "", cex.axis=3,
cex=2.5, pch=19, axes=F)
> axis(side=1,lwd=4,cex.axis=3, labels=NA)
> axis(side=1,lwd=0,cex.axis=3, line=1)
> axis(side=2,lwd=4,cex.axis=3, labels=NA)
> axis(side=2,lwd=0,cex.axis=3, line=0)
>
>
> #F11 vs EXTEM-CT
> cor.test(epi$F11postop, epi$Prov2extemCT, use="complete.obs",
method="pearson")

	Pearson's product-moment correlation

data:  epi$F11postop and epi$Prov2extemCT
t = -0.91083, df = 29, p-value = 0.3699
alternative hypothesis: true correlation is not equal to 0
95 percent confidence interval:
 -0.4920325  0.1993526
sample estimates:
       cor
-0.1667678

> cor.test(epi$F11postop, epi$Prov2extemCT, use="complete.obs",
method="spearman")

	Spearman's rank correlation rho

data:  epi$F11postop and epi$Prov2extemCT
S = 6159.3, p-value = 0.19
alternative hypothesis: true rho is not equal to 0
sample estimates:
       rho
-0.2417941

Warning message:
In cor.test.default(epi$F11postop, epi$Prov2extemCT, use = "complete.obs",  :
  Cannot compute exact p-value with ties
> #RESULT:
> plot(epi$F11postop, epi$Prov2extemCT, xlab="", ylab = "", cex.axis=3,
cex=2.5, pch=19, axes=F)
> axis(side=1,lwd=4,cex.axis=3, labels=NA)
> axis(side=1,lwd=0,cex.axis=3, line=1)
> axis(side=2,lwd=4,cex.axis=3, labels=NA)
> axis(side=2,lwd=0,cex.axis=3, line=0)
>
> #F11 vs INTEM-CT
> cor.test(epi$F11postop, epi$Prov2intemCT, use="complete.obs",
```

```
method="pearson")

    Pearson's product-moment correlation

data:  epi$F11postop and epi$Prov2intemCT
t = -0.030361, df = 29, p-value = 0.976
alternative hypothesis: true correlation is not equal to 0
95 percent confidence interval:
 -0.3592603  0.3494003
sample estimates:
         cor 
-0.005637846 

> cor.test(epi$F11postop, epi$Prov2intemCT, use="complete.obs",
method="spearman")

    Spearman's rank correlation rho

data:  epi$F11postop and epi$Prov2intemCT
S = 4709.8, p-value = 0.7876
alternative hypothesis: true rho is not equal to 0
sample estimates:
       rho 
0.05043373 

Warning message:
In cor.test.default(epi$F11postop, epi$Prov2intemCT, use = "complete.obs",  :
  Cannot compute exact p-value with ties
> #RESULT:
> plot(epi$F11postop, epi$Prov2intemCT, xlab="", ylab = "", cex.axis=3,
cex=2.5, pch=19, axes=F)
> axis(side=1,lwd=4,cex.axis=3, labels=NA)
> axis(side=1,lwd=0,cex.axis=3, line=1)
> axis(side=2,lwd=4,cex.axis=3, labels=NA)
> axis(side=2,lwd=0,cex.axis=3, line=0)
> 
> #F11 vs FIBTEM-MCF
> cor.test(epi$F11postop, epi$Prov2fibtemMCF, use="complete.obs",
method="pearson")

    Pearson's product-moment correlation

data:  epi$F11postop and epi$Prov2fibtemMCF
t = 2.5389, df = 29, p-value = 0.01674
alternative hypothesis: true correlation is not equal to 0
95 percent confidence interval:
 0.08493867 0.67829026
sample estimates:
     cor 
0.426444 

> cor.test(epi$F11postop, epi$Prov2fibtemMCF, use="complete.obs",
method="spearman")

    Spearman's rank correlation rho
```

```
data:  epi$F11postop and epi$Prov2fibtemMCF
S = 3423.5, p-value = 0.08991
alternative hypothesis: true rho is not equal to 0
sample estimates:
      rho
0.3097777

Warning message:
In cor.test.default(epi$F11postop, epi$Prov2fibtemMCF, use = "complete.obs",  :
  Cannot compute exact p-value with ties
> #RESULT:
> plot(epi$F11postop, epi$Prov2fibtemMCF, xlab="", ylab = "", cex.axis=3,
cex=2.5, pch=19, axes=F,abline(lm(epi$Prov2fibtemMCF~ epi
$F11postop),lwd=3,lty=2))
> axis(side=1,lwd=4,cex.axis=3, labels=NA)
> axis(side=1,lwd=0,cex.axis=3, line=1)
> axis(side=2,lwd=4,cex.axis=3, labels=NA)
> axis(side=2,lwd=0,cex.axis=3, line=0)
> text(1.5,19,"r=0.43*",cex=6)
>
> #F12 vs EXTEM-MCF
> cor.test(epi$F12postop, epi$Prov2extemMCF, use="complete.obs",
method="pearson")

    Pearson's product-moment correlation

data:  epi$F12postop and epi$Prov2extemMCF
t = 0.13599, df = 29, p-value = 0.8928
alternative hypothesis: true correlation is not equal to 0
95 percent confidence interval:
 -0.3320659  0.3762193
sample estimates:
       cor
0.02524455

> cor.test(epi$F12postop, epi$Prov2extemMCF, use="complete.obs",
method="spearman")

    Spearman's rank correlation rho

data:  epi$F12postop and epi$Prov2extemMCF
S = 5598.5, p-value = 0.4901
alternative hypothesis: true rho is not equal to 0
sample estimates:
       rho
-0.1287329

Warning message:
In cor.test.default(epi$F12postop, epi$Prov2extemMCF, use = "complete.obs",  :
  Cannot compute exact p-value with ties
> #RESULT:
> plot(epi$F12postop, epi$Prov2extemMCF, xlab="", ylab = "", cex.axis=3,
cex=2.5, pch=19, axes=F)
> axis(side=1,lwd=4,cex.axis=3, labels=NA)
> axis(side=1,lwd=0,cex.axis=3, line=1)
> axis(side=2,lwd=4,cex.axis=3, labels=NA)
```

```
> axis(side=2,lwd=0,cex.axis=3, line=0)
>
> #F12 vs EXTEM-CT
> cor.test(epi$F12postop, epi$Prov2extemCT, use="complete.obs",
method="pearson")

    Pearson's product-moment correlation

data:  epi$F12postop and epi$Prov2extemCT
t = -1.6107, df = 29, p-value = 0.1181
alternative hypothesis: true correlation is not equal to 0
95 percent confidence interval:
 -0.58181687  0.07544813
sample estimates:
       cor
-0.2865528

> cor.test(epi$F12postop, epi$Prov2extemCT, use="complete.obs",
method="spearman")

    Spearman's rank correlation rho

data:  epi$F12postop and epi$Prov2extemCT
S = 6426.7, p-value = 0.1063
alternative hypothesis: true rho is not equal to 0
sample estimates:
       rho
-0.2956983

Warning message:
In cor.test.default(epi$F12postop, epi$Prov2extemCT, use = "complete.obs",  :
  Cannot compute exact p-value with ties
> #RESULT:
> plot(epi$F12postop, epi$Prov2extemCT, xlab="", ylab = "", cex.axis=3,
cex=2.5, pch=19, axes=F)
> axis(side=1,lwd=4,cex.axis=3, labels=NA)
> axis(side=1,lwd=0,cex.axis=3, line=1)
> axis(side=2,lwd=4,cex.axis=3, labels=NA)
> axis(side=2,lwd=0,cex.axis=3, line=0)
>
> #F12 vs INTEM-CT
> cor.test(epi$F12postop, epi$Prov2intemCT, use="complete.obs",
method="pearson")

    Pearson's product-moment correlation

data:  epi$F12postop and epi$Prov2intemCT
t = 0.94101, df = 29, p-value = 0.3545
alternative hypothesis: true correlation is not equal to 0
95 percent confidence interval:
 -0.1940427  0.4962075
sample estimates:
      cor
0.1721328

> cor.test(epi$F12postop, epi$Prov2intemCT, use="complete.obs",
```

```
method="spearman")

	Spearman's rank correlation rho

data:  epi$F12postop and epi$Prov2intemCT
S = 4698.9, p-value = 0.7785
alternative hypothesis: true rho is not equal to 0
sample estimates:
       rho
0.05264751

Warning message:
In cor.test.default(epi$F12postop, epi$Prov2intemCT, use = "complete.obs",  :
  Cannot compute exact p-value with ties
> #RESULT:
> plot(epi$F12postop, epi$Prov2intemCT, xlab="", ylab = "", cex.axis=3,
cex=2.5, pch=19, axes=F)
> axis(side=1,lwd=4,cex.axis=3, labels=NA)
> axis(side=1,lwd=0,cex.axis=3, line=1)
> axis(side=2,lwd=4,cex.axis=3, labels=NA)
> axis(side=2,lwd=0,cex.axis=3, line=0)
>
> #F12 vs FIBTEM-MCF
> cor.test(epi$F12postop, epi$Prov2fibtemMCF, use="complete.obs",
method="pearson")

	Pearson's product-moment correlation

data:  epi$F12postop and epi$Prov2fibtemMCF
t = 0.41489, df = 29, p-value = 0.6813
alternative hypothesis: true correlation is not equal to 0
95 percent confidence interval:
 -0.2852892  0.4197317
sample estimates:
       cor
0.07681624

> cor.test(epi$F12postop, epi$Prov2fibtemMCF, use="complete.obs",
method="spearman")

	Spearman's rank correlation rho

data:  epi$F12postop and epi$Prov2fibtemMCF
S = 4808.6, p-value = 0.8705
alternative hypothesis: true rho is not equal to 0
sample estimates:
       rho
0.03052984

Warning message:
In cor.test.default(epi$F12postop, epi$Prov2fibtemMCF, use = "complete.obs",  :
  Cannot compute exact p-value with ties
> #RESULT:
> plot(epi$F12postop, epi$Prov2fibtemMCF, xlab="", ylab = "", cex.axis=3,
cex=2.5, pch=19, axes=F)
> axis(side=1,lwd=4,cex.axis=3, labels=NA)
```

```
> axis(side=1,lwd=0,cex.axis=3, line=1)
> axis(side=2,lwd=4,cex.axis=3, labels=NA)
> axis(side=2,lwd=0,cex.axis=3, line=0)
>
>
>
>
> #F13 vs EXTEM-MCF
> cor.test(epi$F13postop, epi$Prov2extemMCF, use="complete.obs",
method="pearson")

	Pearson's product-moment correlation

data:  epi$F13postop and epi$Prov2extemMCF
t = 2.6074, df = 29, p-value = 0.01426
alternative hypothesis: true correlation is not equal to 0
95 percent confidence interval:
 0.09631896 0.68443758
sample estimates:
      cor
0.4357859

> cor.test(epi$F13postop, epi$Prov2extemMCF, use="complete.obs",
method="spearman")

	Spearman's rank correlation rho

data:  epi$F13postop and epi$Prov2extemMCF
S = 3359.7, p-value = 0.07668
alternative hypothesis: true rho is not equal to 0
sample estimates:
      rho
0.3226419

Warning message:
In cor.test.default(epi$F13postop, epi$Prov2extemMCF, use = "complete.obs",  :
  Cannot compute exact p-value with ties
> #RESULT:
> plot(epi$F13postop, epi$Prov2extemMCF, xlab="", ylab = "", cex.axis=3,
cex=2.5, pch=19, axes=F,abline(lm(epi$Prov2extemMCF~ epi
$F13postop),lwd=3,lty=2))
> axis(side=1,lwd=4,cex.axis=3, labels=NA)
> axis(side=1,lwd=0,cex.axis=3, line=1)
> axis(side=2,lwd=4,cex.axis=3, labels=NA)
> axis(side=2,lwd=0,cex.axis=3, line=0)
> text(1,60,"r=0.44*",cex=6)
>
>
> #F13 vs EXTEM-CT
> cor.test(epi$F13postop, epi$Prov2extemCT, use="complete.obs",
method="pearson")

	Pearson's product-moment correlation

data:  epi$F13postop and epi$Prov2extemCT
t = -0.96615, df = 29, p-value = 0.342
```

```
alternative hypothesis: true correlation is not equal to 0
95 percent confidence interval:
 -0.4996641  0.1896156
sample estimates:
       cor
-0.1765892

> cor.test(epi$F13postop, epi$Prov2extemCT, use="complete.obs",
method="spearman")

    Spearman's rank correlation rho

data:  epi$F13postop and epi$Prov2extemCT
S = 5848.6, p-value = 0.3349
alternative hypothesis: true rho is not equal to 0
sample estimates:
      rho
-0.179156

Warning message:
In cor.test.default(epi$F13postop, epi$Prov2extemCT, use = "complete.obs",  :
  Cannot compute exact p-value with ties
> #RESULT:
> plot(epi$F13postop, epi$Prov2extemCT, xlab="", ylab = "", cex.axis=3,
cex=2.5, pch=19, axes=F)
> axis(side=1,lwd=4,cex.axis=3, labels=NA)
> axis(side=1,lwd=0,cex.axis=3, line=1)
> axis(side=2,lwd=4,cex.axis=3, labels=NA)
> axis(side=2,lwd=0,cex.axis=3, line=0)
>
> #F13 vs INTEM-CT
> cor.test(epi$F13postop, epi$Prov2intemCT, use="complete.obs",
method="pearson")

    Pearson's product-moment correlation

data:  epi$F13postop and epi$Prov2intemCT
t = 0.18351, df = 29, p-value = 0.8557
alternative hypothesis: true correlation is not equal to 0
95 percent confidence interval:
 -0.3241954  0.3837659
sample estimates:
       cor
0.03405699

> cor.test(epi$F13postop, epi$Prov2intemCT, use="complete.obs",
method="spearman")

    Spearman's rank correlation rho

data:  epi$F13postop and epi$Prov2intemCT
S = 4576.8, p-value = 0.6795
alternative hypothesis: true rho is not equal to 0
sample estimates:
       rho
0.07725669
```

```
Warning message:
In cor.test.default(epi$F13postop, epi$Prov2intemCT, use = "complete.obs",  :
  Cannot compute exact p-value with ties
> #RESULT:
> plot(epi$F13postop, epi$Prov2intemCT, xlab="", ylab = "", cex.axis=3,
cex=2.5, pch=19, axes=F)
> axis(side=1,lwd=4,cex.axis=3, labels=NA)
> axis(side=1,lwd=0,cex.axis=3, line=1)
> axis(side=2,lwd=4,cex.axis=3, labels=NA)
> axis(side=2,lwd=0,cex.axis=3, line=0)
>
> #F13 vs FIBTEM-MCF
> cor.test(epi$F13postop, epi$Prov2fibtemMCF, use="complete.obs",
method="pearson")

    Pearson's product-moment correlation

data:  epi$F13postop and epi$Prov2fibtemMCF
t = 2.8328, df = 29, p-value = 0.008309
alternative hypothesis: true correlation is not equal to 0
95 percent confidence interval:
 0.1331763 0.7037890
sample estimates:
      cor
0.4655473

> cor.test(epi$F13postop, epi$Prov2fibtemMCF, use="complete.obs",
method="spearman")

    Spearman's rank correlation rho

data:  epi$F13postop and epi$Prov2fibtemMCF
S = 2650.5, p-value = 0.008296
alternative hypothesis: true rho is not equal to 0
sample estimates:
      rho
0.4656307

Warning message:
In cor.test.default(epi$F13postop, epi$Prov2fibtemMCF, use = "complete.obs",  :
  Cannot compute exact p-value with ties
> #RESULT:
> plot(epi$F13postop, epi$Prov2fibtemMCF, xlab="", ylab = "", cex.axis=3,
cex=2.5, pch=19, axes=F,abline(lm(epi$Prov2fibtemMCF~ epi
$F13postop)),lwd=3,lty=2)
> axis(side=1,lwd=4,cex.axis=3, labels=NA)
> axis(side=1,lwd=0,cex.axis=3, line=1)
> axis(side=2,lwd=4,cex.axis=3, labels=NA)
> axis(side=2,lwd=0,cex.axis=3, line=0)
> text(.85,20,"Rho=0.47**",cex=6)
>
>
>
>
>
```

```
> #EXTEM-MCF vs PT-INR
> cor.test(epi$Prov2extemMCF, epi$Prov2rutinPK, use="complete.obs",
method="pearson")

	Pearson's product-moment correlation

data:  epi$Prov2extemMCF and epi$Prov2rutinPK
t = -1.4426, df = 31, p-value = 0.1592
alternative hypothesis: true correlation is not equal to 0
95 percent confidence interval:
 -0.5470235  0.1012061
sample estimates:
       cor
-0.2508175

> cor.test(epi$Prov2extemMCF, epi$Prov2rutinPK, use="complete.obs",
method="spearman")

	Spearman's rank correlation rho

data:  epi$Prov2extemMCF and epi$Prov2rutinPK
S = 6546.7, p-value = 0.6027
alternative hypothesis: true rho is not equal to 0
sample estimates:
        rho
-0.09402775

Warning message:
In cor.test.default(epi$Prov2extemMCF, epi$Prov2rutinPK, use =
"complete.obs",  :
  Cannot compute exact p-value with ties
> #RESULT:
> plot(epi$Prov2extemMCF, epi$Prov2rutinPK, xlab="", ylab = "", cex.axis=3,
cex=2.5, pch=19, axes=F)
> axis(side=1,lwd=4,cex.axis=3, labels=NA)
> axis(side=1,lwd=0,cex.axis=3, line=1)
> axis(side=2,lwd=4,cex.axis=3, labels=NA)
> axis(side=2,lwd=0,cex.axis=3, line=0)
>
> #EXTEM-MCF vs aPTT
> cor.test(epi$Prov2extemMCF, epi$Prov2rutinAPTT, use="complete.obs",
method="pearson")

	Pearson's product-moment correlation

data:  epi$Prov2extemMCF and epi$Prov2rutinAPTT
t = -0.16956, df = 31, p-value = 0.8665
alternative hypothesis: true correlation is not equal to 0
95 percent confidence interval:
 -0.3698837  0.3161730
sample estimates:
        cor
-0.03044012

> cor.test(epi$Prov2extemMCF, epi$Prov2rutinAPTT, use="complete.obs",
method="spearman")
```

```
	Spearman's rank correlation rho

data:  epi$Prov2extemMCF and epi$Prov2rutinAPTT
S = 5841.2, p-value = 0.8951
alternative hypothesis: true rho is not equal to 0
sample estimates:
       rho
0.02386784

Warning message:
In cor.test.default(epi$Prov2extemMCF, epi$Prov2rutinAPTT, use =
"complete.obs",  :
  Cannot compute exact p-value with ties
> #RESULT:
> plot(epi$Prov2extemMCF, epi$Prov2rutinAPTT, xlab="", ylab = "", cex.axis=3,
cex=2.5, pch=19, axes=F)
> axis(side=1,lwd=4,cex.axis=3, labels=NA)
> axis(side=1,lwd=0,cex.axis=3, line=1)
> axis(side=2,lwd=4,cex.axis=3, labels=NA)
> axis(side=2,lwd=0,cex.axis=3, line=0)
>
> #EXTEM-MCF vs PIVKA
> cor.test(epi$Prov2extemMCF, epi$PIVKAMpostop, use="complete.obs",
method="pearson")

	Pearson's product-moment correlation

data:  epi$Prov2extemMCF and epi$PIVKAMpostop
t = -0.89872, df = 29, p-value = 0.3762
alternative hypothesis: true correlation is not equal to 0
95 percent confidence interval:
 -0.4903501  0.2014808
sample estimates:
       cor
-0.1646114

> cor.test(epi$Prov2extemMCF, epi$PIVKAMpostop, use="complete.obs",
method="spearman")

	Spearman's rank correlation rho

data:  epi$Prov2extemMCF and epi$PIVKAMpostop
S = 6448.9, p-value = 0.1009
alternative hypothesis: true rho is not equal to 0
sample estimates:
       rho
-0.3001744

Warning message:
In cor.test.default(epi$Prov2extemMCF, epi$PIVKAMpostop, use =
"complete.obs",  :
  Cannot compute exact p-value with ties
> #RESULT:
> plot(epi$Prov2extemMCF, epi$PIVKAMpostop, xlab="", ylab = "", cex.axis=3,
cex=2.5, pch=19, axes=F)
```

```
> axis(side=1,lwd=4,cex.axis=3, labels=NA)
> axis(side=1,lwd=0,cex.axis=3, line=1)
> axis(side=2,lwd=4,cex.axis=3, labels=NA)
> axis(side=2,lwd=0,cex.axis=3, line=0)
>
> #EXTEM-MCF vs CRP
> cor.test(epi$Prov2extemMCF, epi$Prov2rutinCRP, use="complete.obs",
method="pearson")

    Pearson's product-moment correlation

data:  epi$Prov2extemMCF and epi$Prov2rutinCRP
t = -0.58792, df = 28, p-value = 0.5613
alternative hypothesis: true correlation is not equal to 0
95 percent confidence interval:
 -0.4526865  0.2601939
sample estimates:
       cor
-0.1104267

> cor.test(epi$Prov2extemMCF, epi$Prov2rutinCRP, use="complete.obs",
method="spearman")

    Spearman's rank correlation rho

data:  epi$Prov2extemMCF and epi$Prov2rutinCRP
S = 4299.5, p-value = 0.8194
alternative hypothesis: true rho is not equal to 0
sample estimates:
       rho
0.04350263

Warning message:
In cor.test.default(epi$Prov2extemMCF, epi$Prov2rutinCRP, use =
"complete.obs",  :
  Cannot compute exact p-value with ties
> #RESULT:
> plot(epi$Prov2extemMCF, epi$Prov2rutinCRP, xlab="", ylab = "", cex.axis=3,
cex=2.5, pch=19, axes=F)
> axis(side=1,lwd=4,cex.axis=3, labels=NA)
> axis(side=1,lwd=0,cex.axis=3, line=1)
> axis(side=2,lwd=4,cex.axis=3, labels=NA)
> axis(side=2,lwd=0,cex.axis=3, line=0)
>
>
>
> #EXTEM-CT vs PT-INR
> cor.test(epi$Prov2extemCT, epi$Prov2rutinPK, use="complete.obs",
method="pearson")

    Pearson's product-moment correlation

data:  epi$Prov2extemCT and epi$Prov2rutinPK
t = 1.1177, df = 31, p-value = 0.2723
alternative hypothesis: true correlation is not equal to 0
95 percent confidence interval:
```

```
 -0.1571117  0.5059370
sample estimates:
      cor
0.1968129

> cor.test(epi$Prov2extemCT, epi$Prov2rutinPK, use="complete.obs",
method="spearman")

	Spearman's rank correlation rho

data:  epi$Prov2extemCT and epi$Prov2rutinPK
S = 5462.4, p-value = 0.6296
alternative hypothesis: true rho is not equal to 0
sample estimates:
       rho
0.08716727

Warning message:
In cor.test.default(epi$Prov2extemCT, epi$Prov2rutinPK, use =
"complete.obs",  :
  Cannot compute exact p-value with ties
> #RESULT:
> plot(epi$Prov2extemCT, epi$Prov2rutinPK, xlab="", ylab = "", cex.axis=3,
cex=2.5, pch=19, axes=F)
> axis(side=1,lwd=4,cex.axis=3, labels=NA)
> axis(side=1,lwd=0,cex.axis=3, line=1)
> axis(side=2,lwd=4,cex.axis=3, labels=NA)
> axis(side=2,lwd=0,cex.axis=3, line=0)
>
> #EXTEM-CT vs aPTT
> cor.test(epi$Prov2extemCT, epi$Prov2rutinAPTT, use="complete.obs",
method="pearson")

	Pearson's product-moment correlation

data:  epi$Prov2extemCT and epi$Prov2rutinAPTT
t = 0.96335, df = 31, p-value = 0.3428
alternative hypothesis: true correlation is not equal to 0
95 percent confidence interval:
 -0.1835627  0.4853891
sample estimates:
      cor
0.1704904

> cor.test(epi$Prov2extemCT, epi$Prov2rutinAPTT, use="complete.obs",
method="spearman")

	Spearman's rank correlation rho

data:  epi$Prov2extemCT and epi$Prov2rutinAPTT
S = 6474.8, p-value = 0.65
alternative hypothesis: true rho is not equal to 0
sample estimates:
        rho
-0.08202175
```

```
Warning message:
In cor.test.default(epi$Prov2extemCT, epi$Prov2rutinAPTT, use =
"complete.obs",  :
  Cannot compute exact p-value with ties
> #RESULT:
> plot(epi$Prov2extemCT, epi$Prov2rutinAPTT, xlab="", ylab = "", cex.axis=3,
cex=2.5, pch=19, axes=F)
> axis(side=1,lwd=4,cex.axis=3, labels=NA)
> axis(side=1,lwd=0,cex.axis=3, line=1)
> axis(side=2,lwd=4,cex.axis=3, labels=NA)
> axis(side=2,lwd=0,cex.axis=3, line=0)
>
> #EXTEM-CT vs PIVKA
> cor.test(epi$Prov2extemCT, epi$PIVKAMpostop, use="complete.obs",
method="pearson")

	Pearson's product-moment correlation

data:  epi$Prov2extemCT and epi$PIVKAMpostop
t = -0.9842, df = 29, p-value = 0.3332
alternative hypothesis: true correlation is not equal to 0
95 percent confidence interval:
 -0.5021355  0.1864331
sample estimates:
       cor
-0.1797837

> cor.test(epi$Prov2extemCT, epi$PIVKAMpostop, use="complete.obs",
method="spearman")

	Spearman's rank correlation rho

data:  epi$Prov2extemCT and epi$PIVKAMpostop
S = 7649.9, p-value = 0.001624
alternative hypothesis: true rho is not equal to 0
sample estimates:
       rho
-0.5423156

Warning message:
In cor.test.default(epi$Prov2extemCT, epi$PIVKAMpostop, use =
"complete.obs",  :
  Cannot compute exact p-value with ties
> #RESULT:
> plot(epi$Prov2extemCT, epi$PIVKAMpostop, xlab="", ylab = "", cex.axis=2.7,
cex=2.5, pch=19, axes=F)
> axis(side=1,lwd=4,cex.axis=3, labels=NA)
> axis(side=1,lwd=0,cex.axis=3, line=1)
> axis(side=2,lwd=4,cex.axis=2, labels=NA)
> axis(side=2,lwd=0,cex.axis=2.5, line=0)
>
> #EXTEM-CT vs CRP
> cor.test(epi$Prov2extemCT, epi$Prov2rutinCRP, use="complete.obs",
method="pearson")

	Pearson's product-moment correlation
```

```
data:  epi$Prov2extemCT and epi$Prov2rutinCRP
t = 0.16566, df = 28, p-value = 0.8696
alternative hypothesis: true correlation is not equal to 0
95 percent confidence interval:
 -0.3327281  0.3871963
sample estimates:
       cor
0.03129213

> cor.test(epi$Prov2extemCT, epi$Prov2rutinCRP, use="complete.obs",
method="spearman")

    Spearman's rank correlation rho

data:  epi$Prov2extemCT and epi$Prov2rutinCRP
S = 4190.2, p-value = 0.7218
alternative hypothesis: true rho is not equal to 0
sample estimates:
       rho
0.06780432

Warning message:
In cor.test.default(epi$Prov2extemCT, epi$Prov2rutinCRP, use =
"complete.obs",  :
  Cannot compute exact p-value with ties
> #RESULT:
> plot(epi$Prov2extemCT, epi$Prov2rutinCRP, xlab="", ylab = "", cex.axis=3,
cex=2.5, pch=19, axes=F)
> axis(side=1,lwd=4,cex.axis=3, labels=NA)
> axis(side=1,lwd=0,cex.axis=3, line=1)
> axis(side=2,lwd=4,cex.axis=3, labels=NA)
> axis(side=2,lwd=0,cex.axis=3, line=0)
>
>
>
> #FIBTEM-MCF vs PT-INR
> cor.test(epi$Prov2fibtemMCF, epi$Prov2rutinPK, use="complete.obs",
method="pearson")

    Pearson's product-moment correlation

data:  epi$Prov2fibtemMCF and epi$Prov2rutinPK
t = -0.99309, df = 31, p-value = 0.3284
alternative hypothesis: true correlation is not equal to 0
95 percent confidence interval:
 -0.4893990  0.1784758
sample estimates:
       cor
-0.1755922

> cor.test(epi$Prov2fibtemMCF, epi$Prov2rutinPK, use="complete.obs",
method="spearman")

    Spearman's rank correlation rho
```

```
data:  epi$Prov2fibtemMCF and epi$Prov2rutinPK
S = 6137.7, p-value = 0.8872
alternative hypothesis: true rho is not equal to 0
sample estimates:
        rho
-0.02568291

Warning message:
In cor.test.default(epi$Prov2fibtemMCF, epi$Prov2rutinPK, use =
"complete.obs",  :
  Cannot compute exact p-value with ties
> #RESULT:
> plot(epi$Prov2fibtemMCF, epi$Prov2rutinPK, xlab="", ylab = "", cex.axis=3,
cex=2.5, pch=19, axes=F)
> axis(side=1,lwd=4,cex.axis=3, labels=NA)
> axis(side=1,lwd=0,cex.axis=3, line=1)
> axis(side=2,lwd=4,cex.axis=3, labels=NA)
> axis(side=2,lwd=0,cex.axis=3, line=0)
>
> #fibtem-MCF vs aPTT
> cor.test(epi$Prov2fibtemMCF, epi$Prov2rutinAPTT, use="complete.obs",
method="pearson")

    Pearson's product-moment correlation

data:  epi$Prov2fibtemMCF and epi$Prov2rutinAPTT
t = -0.9046, df = 31, p-value = 0.3727
alternative hypothesis: true correlation is not equal to 0
95 percent confidence interval:
 -0.4773939  0.1935995
sample estimates:
       cor
-0.1603683

> cor.test(epi$Prov2fibtemMCF, epi$Prov2rutinAPTT, use="complete.obs",
method="spearman")

    Spearman's rank correlation rho

data:  epi$Prov2fibtemMCF and epi$Prov2rutinAPTT
S = 6003.7, p-value = 0.9855
alternative hypothesis: true rho is not equal to 0
sample estimates:
         rho
-0.003286168

Warning message:
In cor.test.default(epi$Prov2fibtemMCF, epi$Prov2rutinAPTT, use =
"complete.obs",  :
  Cannot compute exact p-value with ties
> #RESULT:
> plot(epi$Prov2fibtemMCF, epi$Prov2rutinAPTT, xlab="", ylab = "", cex.axis=3,
cex=2.5, pch=19, axes=F)
> axis(side=1,lwd=4,cex.axis=3, labels=NA)
> axis(side=1,lwd=0,cex.axis=3, line=1)
> axis(side=2,lwd=4,cex.axis=3, labels=NA)
```

```
> axis(side=2,lwd=0,cex.axis=3, line=0)
>
> #fibtem-MCF vs PIVKA
> cor.test(epi$Prov2fibtemMCF, epi$PIVKAMpostop, use="complete.obs",
method="pearson")

	Pearson's product-moment correlation

data:  epi$Prov2fibtemMCF and epi$PIVKAMpostop
t = -0.64842, df = 29, p-value = 0.5218
alternative hypothesis: true correlation is not equal to 0
95 percent confidence interval:
 -0.4546267  0.2451816
sample estimates:
       cor
-0.1195442

> cor.test(epi$Prov2fibtemMCF, epi$PIVKAMpostop, use="complete.obs",
method="spearman")

	Spearman's rank correlation rho

data:  epi$Prov2fibtemMCF and epi$PIVKAMpostop
S = 5548.7, p-value = 0.5248
alternative hypothesis: true rho is not equal to 0
sample estimates:
       rho
-0.1186822

Warning message:
In cor.test.default(epi$Prov2fibtemMCF, epi$PIVKAMpostop, use =
"complete.obs",  :
  Cannot compute exact p-value with ties
> #RESULT:
> plot(epi$Prov2fibtemMCF, epi$PIVKAMpostop, xlab="", ylab = "", cex.axis=3,
cex=2.5, pch=19, axes=F)
> axis(side=1,lwd=4,cex.axis=3, labels=NA)
> axis(side=1,lwd=0,cex.axis=3, line=1)
> axis(side=2,lwd=4,cex.axis=3, labels=NA)
> axis(side=2,lwd=0,cex.axis=3, line=0)
>
> #fibtem-MCF vs CRP
> cor.test(epi$Prov2fibtemMCF, epi$Prov2rutinCRP, use="complete.obs",
method="pearson")

	Pearson's product-moment correlation

data:  epi$Prov2fibtemMCF and epi$Prov2rutinCRP
t = -0.6738, df = 28, p-value = 0.506
alternative hypothesis: true correlation is not equal to 0
95 percent confidence interval:
 -0.4654053  0.2451080
sample estimates:
       cor
-0.1263155
```

```
> cor.test(epi$Prov2fibtemMCF, epi$Prov2rutinCRP, use="complete.obs",
method="spearman")

	Spearman's rank correlation rho

data:  epi$Prov2fibtemMCF and epi$Prov2rutinCRP
S = 4530.6, p-value = 0.9669
alternative hypothesis: true rho is not equal to 0
sample estimates:
         rho
-0.007921481

Warning message:
In cor.test.default(epi$Prov2fibtemMCF, epi$Prov2rutinCRP, use =
"complete.obs",  :
  Cannot compute exact p-value with ties
> #RESULT:
> plot(epi$Prov2fibtemMCF, epi$Prov2rutinCRP, xlab="", ylab = "", cex.axis=3,
cex=2.5, pch=19, axes=F)
> axis(side=1,lwd=4,cex.axis=3, labels=NA)
> axis(side=1,lwd=0,cex.axis=3, line=1)
> axis(side=2,lwd=4,cex.axis=3, labels=NA)
> axis(side=2,lwd=0,cex.axis=3, line=0)
>
>
>
> #F2 vs PT-INR
> cor.test(epi$F2postop, epi$Prov2rutinPK, use="complete.obs",
method="pearson")

	Pearson's product-moment correlation

data:  epi$F2postop and epi$Prov2rutinPK
t = -2.798, df = 29, p-value = 0.009041
alternative hypothesis: true correlation is not equal to 0
95 percent confidence interval:
 -0.7008942 -0.1275606
sample estimates:
       cor
-0.4610609

> cor.test(epi$F2postop, epi$Prov2rutinPK, use="complete.obs",
method="spearman")

	Spearman's rank correlation rho

data:  epi$F2postop and epi$Prov2rutinPK
S = 7595.9, p-value = 0.002095
alternative hypothesis: true rho is not equal to 0
sample estimates:
       rho
-0.5314268

Warning message:
In cor.test.default(epi$F2postop, epi$Prov2rutinPK, use = "complete.obs",  :
  Cannot compute exact p-value with ties
```

```
> #RESULT:
> plot(epi$F2postop, epi$Prov2rutinPK, xlab="", ylab = "", cex.axis=3, cex=2.5, pch=19, axes=F,abline(lm(epi$Prov2rutinPK~ epi$F2postop),lwd=3))
> axis(side=1,lwd=4,cex.axis=3, labels=NA)
> axis(side=1,lwd=0,cex.axis=3, line=1)
> axis(side=2,lwd=4,cex.axis=3, labels=NA)
> axis(side=2,lwd=0,cex.axis=3, line=0)
> text(1.4,1.5,"r=-0.53**",cex=7)
>
> #F2 vs aPTT
> cor.test(epi$F2postop, epi$Prov2rutinAPTT, use="complete.obs", method="pearson")

	Pearson's product-moment correlation

data:  epi$F2postop and epi$Prov2rutinAPTT
t = -2.8248, df = 29, p-value = 0.008471
alternative hypothesis: true correlation is not equal to 0
95 percent confidence interval:
 -0.7031311 -0.1318968
sample estimates:
       cor
-0.4645266

> cor.test(epi$F2postop, epi$Prov2rutinAPTT, use="complete.obs", method="spearman")

	Spearman's rank correlation rho

data:  epi$F2postop and epi$Prov2rutinAPTT
S = 6623.8, p-value = 0.06508
alternative hypothesis: true rho is not equal to 0
sample estimates:
       rho
-0.3354347

Warning message:
In cor.test.default(epi$F2postop, epi$Prov2rutinAPTT, use = "complete.obs",  :
  Cannot compute exact p-value with ties
> #RESULT:
> plot(epi$F2postop, epi$Prov2rutinAPTT, xlab="", ylab = "", cex.axis=3, cex=2.5, pch=19, axes=F,abline(lm(epi$Prov2rutinAPTT~ epi$F2postop),lwd=3,lty=2))
> axis(side=1,lwd=4,cex.axis=3, labels=NA)
> axis(side=1,lwd=0,cex.axis=3, line=1)
> axis(side=2,lwd=4,cex.axis=3, labels=NA)
> axis(side=2,lwd=0,cex.axis=3, line=0)
> text(1.35,40,"r=-0.46*",cex=8)
>
> #F2 vs PIVKA
> cor.test(epi$F2postop, epi$PIVKAMpostop, use="complete.obs", method="pearson")

	Pearson's product-moment correlation

data:  epi$F2postop and epi$PIVKAMpostop
```

```
t = 0.62829, df = 29, p-value = 0.5347
alternative hypothesis: true correlation is not equal to 0
95 percent confidence interval:
 -0.2486658  0.4516782
sample estimates:
      cor
0.1158852

> cor.test(epi$F2postop, epi$PIVKAMpostop, use="complete.obs",
method="spearman")

	Spearman's rank correlation rho

data:  epi$F2postop and epi$PIVKAMpostop
S = 3200, p-value = 0.05082
alternative hypothesis: true rho is not equal to 0
sample estimates:
      rho
0.3548387

> #RESULT:
> plot(epi$F2postop, epi$PIVKAMpostop, xlab="", ylab = "", cex.axis=3, cex=2.5,
pch=19, axes=F)
> axis(side=1,lwd=4,cex.axis=3, labels=NA)
> axis(side=1,lwd=0,cex.axis=3, line=1)
> axis(side=2,lwd=4,cex.axis=3, labels=NA)
> axis(side=2,lwd=0,cex.axis=3, line=0)
>
> #F2 vs CRP
> cor.test(epi$F2postop, epi$Prov2rutinCRP, use="complete.obs",
method="pearson")

	Pearson's product-moment correlation

data:  epi$F2postop and epi$Prov2rutinCRP
t = -0.77348, df = 26, p-value = 0.4462
alternative hypothesis: true correlation is not equal to 0
95 percent confidence interval:
 -0.4953380  0.2363232
sample estimates:
       cor
-0.1499767

> cor.test(epi$F2postop, epi$Prov2rutinCRP, use="complete.obs",
method="spearman")

	Spearman's rank correlation rho

data:  epi$F2postop and epi$Prov2rutinCRP
S = 4133.1, p-value = 0.506
alternative hypothesis: true rho is not equal to 0
sample estimates:
       rho
-0.1311251

Warning message:
```

```
In cor.test.default(epi$F2postop, epi$Prov2rutinCRP, use = "complete.obs",  :
  Cannot compute exact p-value with ties
> #RESULT:
> plot(epi$F2postop, epi$Prov2rutinCRP, xlab="", ylab = "", cex.axis=3,
cex=2.5, pch=19, axes=F)
> axis(side=1,lwd=4,cex.axis=3, labels=NA)
> axis(side=1,lwd=0,cex.axis=3, line=1)
> axis(side=2,lwd=4,cex.axis=3, labels=NA)
> axis(side=2,lwd=0,cex.axis=3, line=0)
>
>
>
> #F7 vs PT-INR
> cor.test(epi$F7postop, epi$Prov2rutinPK, use="complete.obs",
method="pearson")

	Pearson's product-moment correlation

data:  epi$F7postop and epi$Prov2rutinPK
t = -3.7201, df = 29, p-value = 0.0008505
alternative hypothesis: true correlation is not equal to 0
95 percent confidence interval:
 -0.7680348 -0.2680127
sample estimates:
       cor
-0.5683756

> cor.test(epi$F7postop, epi$Prov2rutinPK, use="complete.obs",
method="spearman")

	Spearman's rank correlation rho

data:  epi$F7postop and epi$Prov2rutinPK
S = 7645.4, p-value = 0.001659
alternative hypothesis: true rho is not equal to 0
sample estimates:
       rho
-0.5414184

Warning message:
In cor.test.default(epi$F7postop, epi$Prov2rutinPK, use = "complete.obs",  :
  Cannot compute exact p-value with ties
> #RESULT:
> plot(epi$F7postop, epi$Prov2rutinPK, xlab="", ylab = "", cex.axis=3, cex=2.5,
pch=19, axes=F,abline(lm(epi$Prov2rutinPK~ epi$F7postop),lwd=3))
> axis(side=1,lwd=4,cex.axis=3, labels=NA)
> axis(side=1,lwd=0,cex.axis=3, line=1)
> axis(side=2,lwd=4,cex.axis=3, labels=NA)
> axis(side=2,lwd=0,cex.axis=3, line=0)
> text(1.2,1.5,"r=-0.57**",cex=6)
>
> #F7 vs aPTT
> cor.test(epi$F7postop, epi$Prov2rutinAPTT, use="complete.obs",
method="pearson")

	Pearson's product-moment correlation
```

```
data:  epi$F7postop and epi$Prov2rutinAPTT
t = -1.8185, df = 29, p-value = 0.07934
alternative hypothesis: true correlation is not equal to 0
95 percent confidence interval:
 -0.60561656  0.03880751
sample estimates:
      cor
-0.319932

> cor.test(epi$F7postop, epi$Prov2rutinAPTT, use="complete.obs",
method="spearman")

    Spearman's rank correlation rho

data:  epi$F7postop and epi$Prov2rutinAPTT
S = 5981, p-value = 0.2666
alternative hypothesis: true rho is not equal to 0
sample estimates:
       rho
-0.2058442

Warning message:
In cor.test.default(epi$F7postop, epi$Prov2rutinAPTT, use = "complete.obs",  :
  Cannot compute exact p-value with ties
> #RESULT:
> plot(epi$F7postop, epi$Prov2rutinAPTT, xlab="", ylab = "", cex.axis=3,
cex=2.5, pch=19, axes=F)
> axis(side=1,lwd=4,cex.axis=3, labels=NA)
> axis(side=1,lwd=0,cex.axis=3, line=1)
> axis(side=2,lwd=4,cex.axis=3, labels=NA)
> axis(side=2,lwd=0,cex.axis=3, line=0)
>
>
> #F7 vs PIVKA
> cor.test(epi$F7postop, epi$PIVKAMpostop, use="complete.obs",
method="pearson")

    Pearson's product-moment correlation

data:  epi$F7postop and epi$PIVKAMpostop
t = 1.2953, df = 29, p-value = 0.2054
alternative hypothesis: true correlation is not equal to 0
95 percent confidence interval:
 -0.1313587  0.5431934
sample estimates:
      cor
0.2338669

> cor.test(epi$F7postop, epi$PIVKAMpostop, use="complete.obs",
method="spearman")

    Spearman's rank correlation rho

data:  epi$F7postop and epi$PIVKAMpostop
S = 3364, p-value = 0.07795
```

```
alternative hypothesis: true rho is not equal to 0
sample estimates:
      rho 
0.3217742 

> #RESULT:
> plot(epi$F7postop, epi$PIVKAMpostop, xlab="", ylab = "", cex.axis=3, cex=2.5, 
pch=19, axes=F)
> axis(side=1,lwd=4,cex.axis=3, labels=NA)
> axis(side=1,lwd=0,cex.axis=3, line=1)
> axis(side=2,lwd=4,cex.axis=3, labels=NA)
> axis(side=2,lwd=0,cex.axis=3, line=0)
> 
> #F7 vs CRP
> cor.test(epi$F7postop, epi$Prov2rutinCRP, use="complete.obs", 
method="pearson")

	Pearson's product-moment correlation

data:  epi$F7postop and epi$Prov2rutinCRP
t = -0.27418, df = 26, p-value = 0.7861
alternative hypothesis: true correlation is not equal to 0
95 percent confidence interval:
 -0.4183889  0.3259125
sample estimates:
        cor 
-0.05369296 

> cor.test(epi$F7postop, epi$Prov2rutinCRP, use="complete.obs", 
method="spearman")

	Spearman's rank correlation rho

data:  epi$F7postop and epi$Prov2rutinCRP
S = 4240.2, p-value = 0.4148
alternative hypothesis: true rho is not equal to 0
sample estimates:
       rho 
-0.1604161 

Warning message:
In cor.test.default(epi$F7postop, epi$Prov2rutinCRP, use = "complete.obs",  :
  Cannot compute exact p-value with ties
> #RESULT:
> plot(epi$F7postop, epi$Prov2rutinCRP, xlab="", ylab = "", cex.axis=3, 
cex=2.5, pch=19, axes=F)
> axis(side=1,lwd=4,cex.axis=3, labels=NA)
> axis(side=1,lwd=0,cex.axis=3, line=1)
> axis(side=2,lwd=4,cex.axis=3, labels=NA)
> axis(side=2,lwd=0,cex.axis=3, line=0)
> 
> 
> 
> #F9 vs PT-INR
> cor.test(epi$F9postop, epi$Prov2rutinPK, use="complete.obs", 
method="pearson")
```

```
	Pearson's product-moment correlation

data:  epi$F9postop and epi$Prov2rutinPK
t = -2.046, df = 29, p-value = 0.04992
alternative hypothesis: true correlation is not equal to 0
95 percent confidence interval:
 -0.6301952979 -0.0009432787
sample estimates:
       cor 
-0.3551647 

> cor.test(epi$F9postop, epi$Prov2rutinPK, use="complete.obs",
method="spearman")

	Spearman's rank correlation rho

data:  epi$F9postop and epi$Prov2rutinPK
S = 6244.4, p-value = 0.1595
alternative hypothesis: true rho is not equal to 0
sample estimates:
       rho 
-0.2589483 

Warning message:
In cor.test.default(epi$F9postop, epi$Prov2rutinPK, use = "complete.obs",  :
  Cannot compute exact p-value with ties
> #RESULT:
> plot(epi$F9postop, epi$Prov2rutinPK, xlab="", ylab = "", cex.axis=3, cex=2.5,
pch=19, axes=F,abline(lm(epi$Prov2rutinPK~ epi$F9postop),lwd=3,lty=2))
> axis(side=1,lwd=4,cex.axis=3, labels=NA)
> axis(side=1,lwd=0,cex.axis=3, line=1)
> axis(side=2,lwd=4,cex.axis=3, labels=NA)
> axis(side=2,lwd=0,cex.axis=3, line=0)
> text(1.95,1.55,"r=-0.36*",cex=7)
> 
> #F9 vs aPTT
> cor.test(epi$F9postop, epi$Prov2rutinAPTT, use="complete.obs",
method="pearson")

	Pearson's product-moment correlation

data:  epi$F9postop and epi$Prov2rutinAPTT
t = -1.2637, df = 29, p-value = 0.2164
alternative hypothesis: true correlation is not equal to 0
95 percent confidence interval:
 -0.5391469  0.1369781
sample estimates:
       cor 
-0.2284503 

> cor.test(epi$F9postop, epi$Prov2rutinAPTT, use="complete.obs",
method="spearman")

	Spearman's rank correlation rho
```

```
data:  epi$F9postop and epi$Prov2rutinAPTT
S = 5896.5, p-value = 0.309
alternative hypothesis: true rho is not equal to 0
sample estimates:
       rho
-0.1888088

Warning message:
In cor.test.default(epi$F9postop, epi$Prov2rutinAPTT, use = "complete.obs",  :
  Cannot compute exact p-value with ties
> #RESULT:
> plot(epi$F9postop, epi$Prov2rutinAPTT, xlab="", ylab = "", cex.axis=3,
cex=2.5, pch=19, axes=F)
> axis(side=1,lwd=4,cex.axis=3, labels=NA)
> axis(side=1,lwd=0,cex.axis=3, line=1)
> axis(side=2,lwd=4,cex.axis=3, labels=NA)
> axis(side=2,lwd=0,cex.axis=3, line=0)
>
>
> #F9 vs PIVKA
> cor.test(epi$F9postop, epi$PIVKAMpostop, use="complete.obs",
method="pearson")

	Pearson's product-moment correlation

data:  epi$F9postop and epi$PIVKAMpostop
t = 0.23775, df = 29, p-value = 0.8137
alternative hypothesis: true correlation is not equal to 0
95 percent confidence interval:
 -0.3151594  0.3923150
sample estimates:
       cor
0.04410625

> cor.test(epi$F9postop, epi$PIVKAMpostop, use="complete.obs",
method="spearman")

	Spearman's rank correlation rho

data:  epi$F9postop and epi$PIVKAMpostop
S = 5260, p-value = 0.7459
alternative hypothesis: true rho is not equal to 0
sample estimates:
        rho
-0.06048387

> #RESULT:
> plot(epi$F9postop, epi$PIVKAMpostop, xlab="", ylab = "", cex.axis=3, cex=2.5,
pch=19, axes=F)
> axis(side=1,lwd=4,cex.axis=3, labels=NA)
> axis(side=1,lwd=0,cex.axis=3, line=1)
> axis(side=2,lwd=4,cex.axis=3, labels=NA)
> axis(side=2,lwd=0,cex.axis=3, line=0)
>
> #F9 vs CRP
> cor.test(epi$F9postop, epi$Prov2rutinCRP, use="complete.obs",
```

```
method="pearson")

	Pearson's product-moment correlation

data:  epi$F9postop and epi$Prov2rutinCRP
t = 1.0772, df = 26, p-value = 0.2913
alternative hypothesis: true correlation is not equal to 0
95 percent confidence interval:
 -0.1802788  0.5382686
sample estimates:
      cor 
0.2067003 

> cor.test(epi$F9postop, epi$Prov2rutinCRP, use="complete.obs", method="spearman")

	Spearman's rank correlation rho

data:  epi$F9postop and epi$Prov2rutinCRP
S = 2792.8, p-value = 0.2273
alternative hypothesis: true rho is not equal to 0
sample estimates:
      rho 
0.2356967 

Warning message:
In cor.test.default(epi$F9postop, epi$Prov2rutinCRP, use = "complete.obs",  :
  Cannot compute exact p-value with ties
> #RESULT:
> plot(epi$F9postop, epi$Prov2rutinCRP, xlab="", ylab = "", cex.axis=3, cex=2.5, pch=19, axes=F)
> axis(side=1,lwd=4,cex.axis=3, labels=NA)
> axis(side=1,lwd=0,cex.axis=3, line=1)
> axis(side=2,lwd=4,cex.axis=3, labels=NA)
> axis(side=2,lwd=0,cex.axis=3, line=0)
> 
> 
> #F10 vs PT-INR
> cor.test(epi$F10postop, epi$Prov2rutinPK, use="complete.obs", method="pearson")

	Pearson's product-moment correlation

data:  epi$F10postop and epi$Prov2rutinPK
t = -2.4963, df = 29, p-value = 0.01848
alternative hypothesis: true correlation is not equal to 0
95 percent confidence interval:
 -0.67440010 -0.07781606
sample estimates:
       cor 
-0.4205599 

> cor.test(epi$F10postop, epi$Prov2rutinPK, use="complete.obs", method="spearman")

	Spearman's rank correlation rho
```

```
data:  epi$F10postop and epi$Prov2rutinPK
S = 6921.9, p-value = 0.02763
alternative hypothesis: true rho is not equal to 0
sample estimates:
       rho
-0.3955396

Warning message:
In cor.test.default(epi$F10postop, epi$Prov2rutinPK, use = "complete.obs",  :
  Cannot compute exact p-value with ties
> #RESULT:
> plot(epi$F10postop, epi$Prov2rutinPK, xlab="", ylab = "", cex.axis=3,
cex=2.5, pch=19, axes=F,abline(lm(epi$Prov2rutinPK~ epi
$F10postop),lwd=3,lty=1))
> axis(side=1,lwd=4,cex.axis=3, labels=NA)
> axis(side=1,lwd=0,cex.axis=3, line=1)
> axis(side=2,lwd=4,cex.axis=3, labels=NA)
> axis(side=2,lwd=0,cex.axis=3, line=0)
> text(1.2,1.55,"r=-0.40*",cex=7)
>
> #F10 vs aPTT
> cor.test(epi$F10postop, epi$Prov2rutinAPTT, use="complete.obs",
method="pearson")

    Pearson's product-moment correlation

data:  epi$F10postop and epi$Prov2rutinAPTT
t = -2.9444, df = 29, p-value = 0.006314
alternative hypothesis: true correlation is not equal to 0
95 percent confidence interval:
 -0.7128916 -0.1510770
sample estimates:
       cor
-0.4797356

> cor.test(epi$F10postop, epi$Prov2rutinAPTT, use="complete.obs",
method="spearman")

    Spearman's rank correlation rho

data:  epi$F10postop and epi$Prov2rutinAPTT
S = 7046.9, p-value = 0.01842
alternative hypothesis: true rho is not equal to 0
sample estimates:
       rho
-0.4207554

Warning message:
In cor.test.default(epi$F10postop, epi$Prov2rutinAPTT, use = "complete.obs",  :
  Cannot compute exact p-value with ties
> #RESULT:
> plot(epi$F10postop, epi$Prov2rutinAPTT, xlab="", ylab = "", cex.axis=3,
cex=2.5, pch=19, axes=F,abline(lm(epi$Prov2rutinAPTT~ epi
$F10postop),lwd=3,lty=1))
> axis(side=1,lwd=4,cex.axis=3, labels=NA)
```

```
> axis(side=1,lwd=0,cex.axis=3, line=1)
> axis(side=2,lwd=4,cex.axis=3, labels=NA)
> axis(side=2,lwd=0,cex.axis=3, line=0)
> text(1.2,40,"r=-0.48*",cex=7)
>
> #F10 vs PIVKA
> cor.test(epi$F10postop, epi$PIVKAMpostop, use="complete.obs",
method="pearson")

    Pearson's product-moment correlation

data:  epi$F10postop and epi$PIVKAMpostop
t = 0.72038, df = 29, p-value = 0.4771
alternative hypothesis: true correlation is not equal to 0
95 percent confidence interval:
 -0.2326811  0.4650804
sample estimates:
      cor
0.1325909

> cor.test(epi$F10postop, epi$PIVKAMpostop, use="complete.obs",
method="spearman")

    Spearman's rank correlation rho

data:  epi$F10postop and epi$PIVKAMpostop
S = 4747, p-value = 0.8186
alternative hypothesis: true rho is not equal to 0
sample estimates:
       rho
0.04294788

Warning message:
In cor.test.default(epi$F10postop, epi$PIVKAMpostop, use = "complete.obs",  :
  Cannot compute exact p-value with ties
> #RESULT:
> plot(epi$F10postop, epi$PIVKAMpostop, xlab="", ylab = "", cex.axis=3,
cex=2.5, pch=19, axes=F)
> axis(side=1,lwd=4,cex.axis=3, labels=NA)
> axis(side=1,lwd=0,cex.axis=3, line=1)
> axis(side=2,lwd=4,cex.axis=3, labels=NA)
> axis(side=2,lwd=0,cex.axis=3, line=0)
>
> #F10 vs CRP
> cor.test(epi$F10postop, epi$Prov2rutinCRP, use="complete.obs",
method="pearson")

    Pearson's product-moment correlation

data:  epi$F10postop and epi$Prov2rutinCRP
t = 0.39461, df = 26, p-value = 0.6963
alternative hypothesis: true correlation is not equal to 0
95 percent confidence interval:
 -0.3046882  0.4376384
sample estimates:
       cor
```

```
0.07715958

> cor.test(epi$F10postop, epi$Prov2rutinCRP, use="complete.obs",
method="spearman")

	Spearman's rank correlation rho

data:  epi$F10postop and epi$Prov2rutinCRP
S = 3224.9, p-value = 0.5517
alternative hypothesis: true rho is not equal to 0
sample estimates:
      rho
0.1174377

Warning message:
In cor.test.default(epi$F10postop, epi$Prov2rutinCRP, use = "complete.obs",  :
  Cannot compute exact p-value with ties
> #RESULT:
> plot(epi$F10postop, epi$Prov2rutinCRP, xlab="", ylab = "", cex.axis=3,
cex=2.5, pch=19, axes=F)
> axis(side=1,lwd=4,cex.axis=3, labels=NA)
> axis(side=1,lwd=0,cex.axis=3, line=1)
> axis(side=2,lwd=4,cex.axis=3, labels=NA)
> axis(side=2,lwd=0,cex.axis=3, line=0)
>
>
> #F11 vs PT-INR
> cor.test(epi$F11postop, epi$Prov2rutinPK, use="complete.obs",
method="pearson")

	Pearson's product-moment correlation

data:  epi$F11postop and epi$Prov2rutinPK
t = -2.7558, df = 29, p-value = 0.01001
alternative hypothesis: true correlation is not equal to 0
95 percent confidence interval:
 -0.6973286 -0.1206935
sample estimates:
       cor
-0.4555513

> cor.test(epi$F11postop, epi$Prov2rutinPK, use="complete.obs",
method="spearman")

	Spearman's rank correlation rho

data:  epi$F11postop and epi$Prov2rutinPK
S = 6819.1, p-value = 0.03775
alternative hypothesis: true rho is not equal to 0
sample estimates:
       rho
-0.3748259

Warning message:
In cor.test.default(epi$F11postop, epi$Prov2rutinPK, use = "complete.obs",  :
  Cannot compute exact p-value with ties
```

```
> #RESULT:
> plot(epi$F11postop, epi$Prov2rutinPK, xlab="", ylab = "", cex.axis=3,
cex=2.5, pch=19, axes=F,abline(lm(epi$Prov2rutinPK~ epi
$F11postop),lwd=3,lty=1))
> axis(side=1,lwd=4,cex.axis=3, labels=NA)
> axis(side=1,lwd=0,cex.axis=3, line=1)
> axis(side=2,lwd=4,cex.axis=3, labels=NA)
> axis(side=2,lwd=0,cex.axis=3, line=0)
> text(1.35,1.55,"r=-0.45*",cex=7)
>
> #F11 vs aPTT
> cor.test(epi$F11postop, epi$Prov2rutinAPTT, use="complete.obs",
method="pearson")

    Pearson's product-moment correlation

data:  epi$F11postop and epi$Prov2rutinAPTT
t = -3.7572, df = 29, p-value = 0.0007701
alternative hypothesis: true correlation is not equal to 0
95 percent confidence interval:
 -0.7703469 -0.2732601
sample estimates:
       cor
-0.5721961

> cor.test(epi$F11postop, epi$Prov2rutinAPTT, use="complete.obs",
method="spearman")

    Spearman's rank correlation rho

data:  epi$F11postop and epi$Prov2rutinAPTT
S = 7650.5, p-value = 0.001618
alternative hypothesis: true rho is not equal to 0
sample estimates:
      rho
-0.542449

Warning message:
In cor.test.default(epi$F11postop, epi$Prov2rutinAPTT, use = "complete.obs",  :
  Cannot compute exact p-value with ties
> #RESULT:
> plot(epi$F11postop, epi$Prov2rutinAPTT, xlab="", ylab = "", cex.axis=3,
cex=2.5, pch=19, axes=F,abline(lm(epi$Prov2rutinAPTT~ epi
$F11postop),lwd=3,lty=1))
> axis(side=1,lwd=4,cex.axis=3, labels=NA)
> axis(side=1,lwd=0,cex.axis=3, line=1)
> axis(side=2,lwd=4,cex.axis=3, labels=NA)
> axis(side=2,lwd=0,cex.axis=3, line=0)
> text(1.2,40,"r=-0.48*",cex=7)
>
> #F11 vs PIVKA
> cor.test(epi$F11postop, epi$PIVKAMpostop, use="complete.obs",
method="pearson")

    Pearson's product-moment correlation
```

```
data:  epi$F11postop and epi$PIVKAMpostop
t = 1.344, df = 29, p-value = 0.1894
alternative hypothesis: true correlation is not equal to 0
95 percent confidence interval:
 -0.1227165  0.5493561
sample estimates:
      cor 
0.2421533 

> cor.test(epi$F11postop, epi$PIVKAMpostop, use="complete.obs", 
method="spearman")

	Spearman's rank correlation rho

data:  epi$F11postop and epi$PIVKAMpostop
S = 3231.8, p-value = 0.05475
alternative hypothesis: true rho is not equal to 0
sample estimates:
      rho 
0.3484222 

Warning message:
In cor.test.default(epi$F11postop, epi$PIVKAMpostop, use = "complete.obs",  :
  Cannot compute exact p-value with ties
> #RESULT:
> plot(epi$F11postop, epi$PIVKAMpostop, xlab="", ylab = "", cex.axis=3, 
cex=2.5, pch=19, axes=F)
> axis(side=1,lwd=4,cex.axis=3, labels=NA)
> axis(side=1,lwd=0,cex.axis=3, line=1)
> axis(side=2,lwd=4,cex.axis=3, labels=NA)
> axis(side=2,lwd=0,cex.axis=3, line=0)
> 
> #F11 vs CRP
> cor.test(epi$F11postop, epi$Prov2rutinCRP, use="complete.obs", 
method="pearson")

	Pearson's product-moment correlation

data:  epi$F11postop and epi$Prov2rutinCRP
t = -1.3216, df = 26, p-value = 0.1978
alternative hypothesis: true correlation is not equal to 0
95 percent confidence interval:
 -0.5705603  0.1348068
sample estimates:
       cor 
-0.2508881 

> cor.test(epi$F11postop, epi$Prov2rutinCRP, use="complete.obs", 
method="spearman")

	Spearman's rank correlation rho

data:  epi$F11postop and epi$Prov2rutinCRP
S = 4382.3, p-value = 0.3092
alternative hypothesis: true rho is not equal to 0
sample estimates:
```

```
       rho
-0.1993155

Warning message:
In cor.test.default(epi$F11postop, epi$Prov2rutinCRP, use = "complete.obs",  :
  Cannot compute exact p-value with ties
> #RESULT:
> plot(epi$F11postop, epi$Prov2rutinCRP, xlab="", ylab = "", cex.axis=3,
cex=2.5, pch=19, axes=F)
> axis(side=1,lwd=4,cex.axis=3, labels=NA)
> axis(side=1,lwd=0,cex.axis=3, line=1)
> axis(side=2,lwd=4,cex.axis=3, labels=NA)
> axis(side=2,lwd=0,cex.axis=3, line=0)
>
>
>
> #F12 vs PT-INR
> cor.test(epi$F12postop, epi$Prov2rutinPK, use="complete.obs",
method="pearson")

    Pearson's product-moment correlation

data:  epi$F12postop and epi$Prov2rutinPK
t = -1.9473, df = 29, p-value = 0.06124
alternative hypothesis: true correlation is not equal to 0
95 percent confidence interval:
 -0.61972276  0.01624047
sample estimates:
       cor
-0.3400566

> cor.test(epi$F12postop, epi$Prov2rutinPK, use="complete.obs",
method="spearman")

    Spearman's rank correlation rho

data:  epi$F12postop and epi$Prov2rutinPK
S = 6396.2, p-value = 0.1141
alternative hypothesis: true rho is not equal to 0
sample estimates:
       rho
-0.2895475

Warning message:
In cor.test.default(epi$F12postop, epi$Prov2rutinPK, use = "complete.obs",  :
  Cannot compute exact p-value with ties
> #RESULT:
> plot(epi$F12postop, epi$Prov2rutinPK, xlab="", ylab = "", cex.axis=3,
cex=2.5, pch=19, axes=F)
> axis(side=1,lwd=4,cex.axis=3, labels=NA)
> axis(side=1,lwd=0,cex.axis=3, line=1)
> axis(side=2,lwd=4,cex.axis=3, labels=NA)
> axis(side=2,lwd=0,cex.axis=3, line=0)
>
>
> #F12 vs aPTT
```

```
> cor.test(epi$F12postop, epi$Prov2rutinAPTT, use="complete.obs",
method="pearson")

    Pearson's product-moment correlation

data:  epi$F12postop and epi$Prov2rutinAPTT
t = -3.2114, df = 29, p-value = 0.003223
alternative hypothesis: true correlation is not equal to 0
95 percent confidence interval:
 -0.7334190 -0.1928443
sample estimates:
       cor
-0.5121855

> cor.test(epi$F12postop, epi$Prov2rutinAPTT, use="complete.obs",
method="spearman")

    Spearman's rank correlation rho

data:  epi$F12postop and epi$Prov2rutinAPTT
S = 7614.6, p-value = 0.00192
alternative hypothesis: true rho is not equal to 0
sample estimates:
       rho
-0.5351948

Warning message:
In cor.test.default(epi$F12postop, epi$Prov2rutinAPTT, use = "complete.obs",  :
  Cannot compute exact p-value with ties
> #RESULT:
> plot(epi$F12postop, epi$Prov2rutinAPTT, xlab="", ylab = "", cex.axis=3,
cex=2.5, pch=19, axes=F,abline(lm(epi$Prov2rutinAPTT~ epi
$F12postop),lwd=3,lty=1))
> axis(side=1,lwd=4,cex.axis=3, labels=NA)
> axis(side=1,lwd=0,cex.axis=3, line=1)
> axis(side=2,lwd=4,cex.axis=3, labels=NA)
> axis(side=2,lwd=0,cex.axis=3, line=0)
> text(1.1,40,"r=-0.51**",cex=7)
>
> #F12 vs PIVKA
> cor.test(epi$F12postop, epi$PIVKAMpostop, use="complete.obs",
method="pearson")

    Pearson's product-moment correlation

data:  epi$F12postop and epi$PIVKAMpostop
t = 2.0018, df = 29, p-value = 0.05474
alternative hypothesis: true correlation is not equal to 0
95 percent confidence interval:
 -0.006736402  0.625543087
sample estimates:
      cor
0.3484354

> cor.test(epi$F12postop, epi$PIVKAMpostop, use="complete.obs",
method="spearman")
```

```
	Spearman's rank correlation rho

data:  epi$F12postop and epi$PIVKAMpostop
S = 1978, p-value = 0.0004509
alternative hypothesis: true rho is not equal to 0
sample estimates:
      rho 
0.6012097 

> #RESULT:
> plot(epi$F12postop, epi$PIVKAMpostop, xlab="", ylab = "", cex.axis=3,
cex=2.5, pch=19, axes=F,ylim=c(0,110),abline(lm(epi$PIVKAMpostop~epi
$F12postop),lwd=3,lty=2))
> axis(side=1,lwd=4,cex.axis=3, labels=NA)
> axis(side=1,lwd=0,cex.axis=3, line=1)
> axis(side=2,lwd=4,cex.axis=3, labels=NA)
> axis(side=2,lwd=0,cex.axis=3, line=0)
> text(.95,90,"Rho=0.60**",cex=6)
> 
> #F12 vs CRP
> cor.test(epi$F12postop, epi$Prov2rutinCRP, use="complete.obs",
method="pearson")

	Pearson's product-moment correlation

data:  epi$F12postop and epi$Prov2rutinCRP
t = -3.4561, df = 26, p-value = 0.001896
alternative hypothesis: true correlation is not equal to 0
95 percent confidence interval:
 -0.7724510 -0.2377528
sample estimates:
       cor 
-0.5610634 

> cor.test(epi$F12postop, epi$Prov2rutinCRP, use="complete.obs",
method="spearman")

	Spearman's rank correlation rho

data:  epi$F12postop and epi$Prov2rutinCRP
S = 5854.6, p-value = 0.0006964
alternative hypothesis: true rho is not equal to 0
sample estimates:
       rho 
-0.6022448 

Warning message:
In cor.test.default(epi$F12postop, epi$Prov2rutinCRP, use = "complete.obs",  :
  Cannot compute exact p-value with ties
> #RESULT:
> plot(epi$F12postop, epi$Prov2rutinCRP, xlab="", ylab = "", cex.axis=3,
cex=2.5, pch=19, axes=F,abline(lm(epi$Prov2rutinCRP~ epi
$F12postop),lwd=3,lty=1))
> axis(side=1,lwd=4,cex.axis=3, labels=NA)
> axis(side=1,lwd=0,cex.axis=3, line=1)
```

```
> axis(side=2,lwd=4,cex.axis=3, labels=NA)
> axis(side=2,lwd=0,cex.axis=3, line=0)
> text(.95,250,"r=-0.56**",cex=6)
>
>
>
>
> #F13 vs PT-INR
> cor.test(epi$F13postop, epi$Prov2rutinPK, use="complete.obs",
method="pearson")

	Pearson's product-moment correlation

data:  epi$F13postop and epi$Prov2rutinPK
t = -2.035, df = 29, p-value = 0.05109
alternative hypothesis: true correlation is not equal to 0
95 percent confidence interval:
 -0.6290361706  0.0009771294
sample estimates:
       cor
-0.3534854

> cor.test(epi$F13postop, epi$Prov2rutinPK, use="complete.obs",
method="spearman")

	Spearman's rank correlation rho

data:  epi$F13postop and epi$Prov2rutinPK
S = 6076.1, p-value = 0.2236
alternative hypothesis: true rho is not equal to 0
sample estimates:
       rho
-0.2250186

Warning message:
In cor.test.default(epi$F13postop, epi$Prov2rutinPK, use = "complete.obs",  :
  Cannot compute exact p-value with ties
> #RESULT:
> plot(epi$F13postop, epi$Prov2rutinPK, xlab="", ylab = "", cex.axis=3,
cex=2.5, pch=19, axes=F)
> axis(side=1,lwd=4,cex.axis=3, labels=NA)
> axis(side=1,lwd=0,cex.axis=3, line=1)
> axis(side=2,lwd=4,cex.axis=3, labels=NA)
> axis(side=2,lwd=0,cex.axis=3, line=0)
>
>
> #F13 vs aPTT
> cor.test(epi$F13postop, epi$Prov2rutinAPTT, use="complete.obs",
method="pearson")

	Pearson's product-moment correlation

data:  epi$F13postop and epi$Prov2rutinAPTT
t = -1.1949, df = 29, p-value = 0.2418
alternative hypothesis: true correlation is not equal to 0
95 percent confidence interval:
```

```
 -0.5302610  0.1491672
sample estimates:
       cor 
-0.2166227 

> cor.test(epi$F13postop, epi$Prov2rutinAPTT, use="complete.obs", 
method="spearman")

	Spearman's rank correlation rho

data:  epi$F13postop and epi$Prov2rutinAPTT
S = 5905.5, p-value = 0.3043
alternative hypothesis: true rho is not equal to 0
sample estimates:
      rho 
-0.190634 

Warning message:
In cor.test.default(epi$F13postop, epi$Prov2rutinAPTT, use = "complete.obs",  :
  Cannot compute exact p-value with ties
> #RESULT:
> plot(epi$F13postop, epi$Prov2rutinAPTT, xlab="", ylab = "", cex.axis=3, 
cex=2.5, pch=19, axes=F)
> axis(side=1,lwd=4,cex.axis=3, labels=NA)
> axis(side=1,lwd=0,cex.axis=3, line=1)
> axis(side=2,lwd=4,cex.axis=3, labels=NA)
> axis(side=2,lwd=0,cex.axis=3, line=0)
> 
> 
> #F13 vs PIVKA
> cor.test(epi$F13postop, epi$PIVKAMpostop, use="complete.obs", 
method="pearson")

	Pearson's product-moment correlation

data:  epi$F13postop and epi$PIVKAMpostop
t = -0.49414, df = 29, p-value = 0.6249
alternative hypothesis: true correlation is not equal to 0
95 percent confidence interval:
 -0.4317363  0.2717645
sample estimates:
        cor 
-0.09137479 

> cor.test(epi$F13postop, epi$PIVKAMpostop, use="complete.obs", 
method="spearman")

	Spearman's rank correlation rho

data:  epi$F13postop and epi$PIVKAMpostop
S = 3830, p-value = 0.2168
alternative hypothesis: true rho is not equal to 0
sample estimates:
      rho 
0.2278226 
```

```
> #RESULT:
> plot(epi$F13postop, epi$PIVKAMpostop, xlab="", ylab = "", cex.axis=3,
cex=2.5, pch=19, axes=F)
> axis(side=1,lwd=4,cex.axis=3, labels=NA)
> axis(side=1,lwd=0,cex.axis=3, line=1)
> axis(side=2,lwd=4,cex.axis=3, labels=NA)
> axis(side=2,lwd=0,cex.axis=3, line=0)
>
>
> #F13 vs CRP
> cor.test(epi$F13postop, epi$Prov2rutinCRP, use="complete.obs",
method="pearson")

	Pearson's product-moment correlation

data:  epi$F13postop and epi$Prov2rutinCRP
t = -0.27037, df = 26, p-value = 0.789
alternative hypothesis: true correlation is not equal to 0
95 percent confidence interval:
 -0.4177741  0.3265782
sample estimates:
        cor
-0.05295006

> cor.test(epi$F13postop, epi$Prov2rutinCRP, use="complete.obs",
method="spearman")

	Spearman's rank correlation rho

data:  epi$F13postop and epi$Prov2rutinCRP
S = 3859.1, p-value = 0.7767
alternative hypothesis: true rho is not equal to 0
sample estimates:
        rho
-0.05611826

Warning message:
In cor.test.default(epi$F13postop, epi$Prov2rutinCRP, use = "complete.obs",  :
  Cannot compute exact p-value with ties
> #RESULT:
> plot(epi$F13postop, epi$Prov2rutinCRP, xlab="", ylab = "", cex.axis=3,
cex=2.5, pch=19, axes=F)
> axis(side=1,lwd=4,cex.axis=3, labels=NA)
> axis(side=1,lwd=0,cex.axis=3, line=1)
> axis(side=2,lwd=4,cex.axis=3, labels=NA)
> axis(side=2,lwd=0,cex.axis=3, line=0)
>
>
>
>
> #Fibrinogen vs PT-INR
> cor.test(epi$FIBpostop, epi$Prov2rutinPK, use="complete.obs",
method="pearson")

	Pearson's product-moment correlation
```

```
data:  epi$FIBpostop and epi$Prov2rutinPK
t = -0.25778, df = 29, p-value = 0.7984
alternative hypothesis: true correlation is not equal to 0
95 percent confidence interval:
 -0.3954537  0.3118094
sample estimates:
        cor
-0.04781344

> cor.test(epi$FIBpostop, epi$Prov2rutinPK, use="complete.obs",
method="spearman")

    Spearman's rank correlation rho

data:  epi$FIBpostop and epi$Prov2rutinPK
S = 4845, p-value = 0.9015
alternative hypothesis: true rho is not equal to 0
sample estimates:
       rho
0.02318511

Warning message:
In cor.test.default(epi$FIBpostop, epi$Prov2rutinPK, use = "complete.obs",  :
  Cannot compute exact p-value with ties
> #RESULT:
> plot(epi$FIBpostop, epi$Prov2rutinPK, xlab="", ylab = "", cex.axis=3,
cex=2.5, pch=19, axes=F)
> axis(side=1,lwd=4,cex.axis=3, labels=NA)
> axis(side=1,lwd=0,cex.axis=3, line=1)
> axis(side=2,lwd=4,cex.axis=3, labels=NA)
> axis(side=2,lwd=0,cex.axis=3, line=0)
>
>
> #FIB vs aPTT
> cor.test(epi$FIBpostop, epi$Prov2rutinAPTT, use="complete.obs",
method="pearson")

    Pearson's product-moment correlation

data:  epi$FIBpostop and epi$Prov2rutinAPTT
t = -0.033445, df = 29, p-value = 0.9735
alternative hypothesis: true correlation is not equal to 0
95 percent confidence interval:
 -0.3597589  0.3488974
sample estimates:
         cor
-0.006210473

> cor.test(epi$FIBpostop, epi$Prov2rutinAPTT, use="complete.obs",
method="spearman")

    Spearman's rank correlation rho

data:  epi$FIBpostop and epi$Prov2rutinAPTT
S = 4704.6, p-value = 0.7833
alternative hypothesis: true rho is not equal to 0
```

```
sample estimates:
       rho
0.05148571

Warning message:
In cor.test.default(epi$FIBpostop, epi$Prov2rutinAPTT, use = "complete.obs",  :
  Cannot compute exact p-value with ties
> #RESULT:
> plot(epi$FIBpostop, epi$Prov2rutinAPTT, xlab="", ylab = "", cex.axis=3,
cex=2.5, pch=19, axes=F)
> axis(side=1,lwd=4,cex.axis=3, labels=NA)
> axis(side=1,lwd=0,cex.axis=3, line=1)
> axis(side=2,lwd=4,cex.axis=3, labels=NA)
> axis(side=2,lwd=0,cex.axis=3, line=0)
>
>
> #FIB vs PIVKA
> cor.test(epi$FIBpostop, epi$PIVKAMpostop, use="complete.obs",
method="pearson")

	Pearson's product-moment correlation

data:  epi$FIBpostop and epi$PIVKAMpostop
t = 0.92809, df = 29, p-value = 0.361
alternative hypothesis: true correlation is not equal to 0
95 percent confidence interval:
 -0.1963158  0.4944241
sample estimates:
      cor
0.1698387

> cor.test(epi$FIBpostop, epi$PIVKAMpostop, use="complete.obs",
method="spearman")

	Spearman's rank correlation rho

data:  epi$FIBpostop and epi$PIVKAMpostop
S = 3822.1, p-value = 0.2144
alternative hypothesis: true rho is not equal to 0
sample estimates:
      rho
0.2294167

Warning message:
In cor.test.default(epi$FIBpostop, epi$PIVKAMpostop, use = "complete.obs",  :
  Cannot compute exact p-value with ties
> #RESULT:
> plot(epi$FIBpostop, epi$PIVKAMpostop, xlab="", ylab = "", cex.axis=3,
cex=2.5, pch=19, axes=F)
> axis(side=1,lwd=4,cex.axis=3, labels=NA)
> axis(side=1,lwd=0,cex.axis=3, line=1)
> axis(side=2,lwd=4,cex.axis=3, labels=NA)
> axis(side=2,lwd=0,cex.axis=3, line=0)
>
>
> #FIB vs CRP
```

```
> cor.test(epi$FIBpostop, epi$Prov2rutinCRP, use="complete.obs",
method="pearson")

	Pearson's product-moment correlation

data:  epi$FIBpostop and epi$Prov2rutinCRP
t = 1.5779, df = 26, p-value = 0.1267
alternative hypothesis: true correlation is not equal to 0
95 percent confidence interval:
 -0.08706656  0.60226770
sample estimates:
      cor
0.2956126

> cor.test(epi$FIBpostop, epi$Prov2rutinCRP, use="complete.obs",
method="spearman")

	Spearman's rank correlation rho

data:  epi$FIBpostop and epi$Prov2rutinCRP
S = 2812, p-value = 0.2381
alternative hypothesis: true rho is not equal to 0
sample estimates:
      rho
0.2304426

Warning message:
In cor.test.default(epi$FIBpostop, epi$Prov2rutinCRP, use = "complete.obs",  :
  Cannot compute exact p-value with ties
> #RESULT:
> plot(epi$FIBpostop, epi$Prov2rutinCRP, xlab="", ylab = "", cex.axis=3,
cex=2.5, pch=19, axes=F)
> axis(side=1,lwd=4,cex.axis=3, labels=NA)
> axis(side=1,lwd=0,cex.axis=3, line=1)
> axis(side=2,lwd=4,cex.axis=3, labels=NA)
> axis(side=2,lwd=0,cex.axis=3, line=0)
>
>
>
> #PIVKA vs PT-INR
> cor.test(epi$PIVKAMpostop, epi$Prov2rutinPK, use="complete.obs",
method="pearson")

	Pearson's product-moment correlation

data:  epi$PIVKAMpostop and epi$Prov2rutinPK
t = -0.61612, df = 29, p-value = 0.5426
alternative hypothesis: true correlation is not equal to 0
95 percent confidence interval:
 -0.4498893  0.2507709
sample estimates:
       cor
-0.1136697

> cor.test(epi$PIVKAMpostop, epi$Prov2rutinPK, use="complete.obs",
method="spearman")
```

```
	Spearman's rank correlation rho

data:  epi$PIVKAMpostop and epi$Prov2rutinPK
S = 5061.2, p-value = 0.9133
alternative hypothesis: true rho is not equal to 0
sample estimates:
        rho 
-0.02039946 

Warning message:
In cor.test.default(epi$PIVKAMpostop, epi$Prov2rutinPK, use = 
"complete.obs",  :
  Cannot compute exact p-value with ties
> #RESULT:
> plot(epi$PIVKAMpostop, epi$Prov2rutinPK, xlab="", ylab = "", cex.axis=3, 
cex=2.5, pch=19, axes=F, xlim=c(0,140))
> axis(side=1,lwd=4,cex.axis=3, labels=NA)
> axis(side=1,lwd=0,cex.axis=3, line=1)
> axis(side=2,lwd=4,cex.axis=3, labels=NA)
> axis(side=2,lwd=0,cex.axis=3, line=0)
> 
> 
> #PIVKAM vs aPTT
> cor.test(epi$PIVKAMpostop, epi$Prov2rutinAPTT, use="complete.obs", 
method="pearson")

	Pearson's product-moment correlation

data:  epi$PIVKAMpostop and epi$Prov2rutinAPTT
t = -1.239, df = 29, p-value = 0.2253
alternative hypothesis: true correlation is not equal to 0
95 percent confidence interval:
 -0.5359820  0.1413431
sample estimates:
       cor 
-0.2242271 

> cor.test(epi$PIVKAMpostop, epi$Prov2rutinAPTT, use="complete.obs", 
method="spearman")

	Spearman's rank correlation rho

data:  epi$PIVKAMpostop and epi$Prov2rutinAPTT
S = 5129, p-value = 0.8556
alternative hypothesis: true rho is not equal to 0
sample estimates:
        rho 
-0.03407076 

Warning message:
In cor.test.default(epi$PIVKAMpostop, epi$Prov2rutinAPTT, use = 
"complete.obs",  :
  Cannot compute exact p-value with ties
> #RESULT:
> plot(epi$PIVKAMpostop, epi$Prov2rutinAPTT, xlab="", ylab = "", cex.axis=3, 
```

```
cex=2.5, pch=19, axes=F, xlim=c(0,140))
> axis(side=1,lwd=4,cex.axis=3, labels=NA)
> axis(side=1,lwd=0,cex.axis=3, line=1)
> axis(side=2,lwd=4,cex.axis=3, labels=NA)
> axis(side=2,lwd=0,cex.axis=3, line=0)
>
>
> #PIVKAM vs PIVKA
> cor.test(epi$PIVKAMpostop, epi$PIVKAMpostop, use="complete.obs",
method="pearson")

	Pearson's product-moment correlation

data:  epi$PIVKAMpostop and epi$PIVKAMpostop
t = Inf, df = 29, p-value < 2.2e-16
alternative hypothesis: true correlation is not equal to 0
95 percent confidence interval:
 1 1
sample estimates:
cor
  1

> cor.test(epi$PIVKAMpostop, epi$PIVKAMpostop, use="complete.obs",
method="spearman")

	Spearman's rank correlation rho

data:  epi$PIVKAMpostop and epi$PIVKAMpostop
S = 0, p-value < 2.2e-16
alternative hypothesis: true rho is not equal to 0
sample estimates:
rho
  1

> #RESULT:
> plot(epi$PIVKAMpostop, epi$PIVKAMpostop, xlab="", ylab = "", cex.axis=3,
cex=2.5, pch=19, axes=F)
> axis(side=1,lwd=4,cex.axis=3, labels=NA)
> axis(side=1,lwd=0,cex.axis=3, line=1)
> axis(side=2,lwd=4,cex.axis=3, labels=NA)
> axis(side=2,lwd=0,cex.axis=3, line=0)
>
>
> #PIVKAM vs CRP
> cor.test(epi$PIVKAMpostop, epi$Prov2rutinCRP, use="complete.obs",
method="pearson")

	Pearson's product-moment correlation

data:  epi$PIVKAMpostop and epi$Prov2rutinCRP
t = -1.0739, df = 26, p-value = 0.2927
alternative hypothesis: true correlation is not equal to 0
95 percent confidence interval:
 -0.5378138  0.1808981
sample estimates:
       cor
```

```
-0.2060874

> cor.test(epi$PIVKAMpostop, epi$Prov2rutinCRP, use="complete.obs",
method="spearman")

	Spearman's rank correlation rho

data:  epi$PIVKAMpostop and epi$Prov2rutinCRP
S = 4800.3, p-value = 0.104
alternative hypothesis: true rho is not equal to 0
sample estimates:
       rho
-0.3137148

Warning message:
In cor.test.default(epi$PIVKAMpostop, epi$Prov2rutinCRP, use =
"complete.obs",  :
  Cannot compute exact p-value with ties
> #RESULT:
> plot(epi$PIVKAMpostop, epi$Prov2rutinCRP, xlab="", ylab = "", cex.axis=3,
cex=2.5, pch=19, axes=F, xlim=c(0,140))
> axis(side=1,lwd=4,cex.axis=3, labels=NA)
> axis(side=1,lwd=0,cex.axis=3, line=1)
> axis(side=2,lwd=4,cex.axis=3, labels=NA)
> axis(side=2,lwd=0,cex.axis=3, line=0)
>
>
> #Plc vs PT-INR
> cor.test(epi$Prov2rutinTPK, epi$Prov2rutinPK, use="complete.obs",
method="pearson")

	Pearson's product-moment correlation

data:  epi$Prov2rutinTPK and epi$Prov2rutinPK
t = -0.51222, df = 29, p-value = 0.6124
alternative hypothesis: true correlation is not equal to 0
95 percent confidence interval:
 -0.4344523  0.2686654
sample estimates:
        cor
-0.09468899

> cor.test(epi$Prov2rutinTPK, epi$Prov2rutinPK, use="complete.obs",
method="spearman")

	Spearman's rank correlation rho

data:  epi$Prov2rutinTPK and epi$Prov2rutinPK
S = 5501.5, p-value = 0.5588
alternative hypothesis: true rho is not equal to 0
sample estimates:
       rho
-0.1091649

Warning message:
In cor.test.default(epi$Prov2rutinTPK, epi$Prov2rutinPK, use =
```

```
"complete.obs",  :
  Cannot compute exact p-value with ties
> #RESULT:
> plot(epi$Prov2rutinTPK, epi$Prov2rutinPK, xlab="", ylab = "", cex.axis=3,
cex=2.5, pch=19, axes=F)
> axis(side=1,lwd=4,cex.axis=3, labels=NA)
> axis(side=1,lwd=0,cex.axis=3, line=1)
> axis(side=2,lwd=4,cex.axis=3, labels=NA)
> axis(side=2,lwd=0,cex.axis=3, line=0)
>
>
> #Prov2rutinTPK vs aPTT
> cor.test(epi$Prov2rutinTPK, epi$Prov2rutinAPTT, use="complete.obs",
method="pearson")

	Pearson's product-moment correlation

data:  epi$Prov2rutinTPK and epi$Prov2rutinAPTT
t = -0.6246, df = 29, p-value = 0.5371
alternative hypothesis: true correlation is not equal to 0
95 percent confidence interval:
 -0.4511352  0.2493055
sample estimates:
       cor
-0.1152124

> cor.test(epi$Prov2rutinTPK, epi$Prov2rutinAPTT, use="complete.obs",
method="spearman")

	Spearman's rank correlation rho

data:  epi$Prov2rutinTPK and epi$Prov2rutinAPTT
S = 5473.7, p-value = 0.5793
alternative hypothesis: true rho is not equal to 0
sample estimates:
       rho
-0.1035621

Warning message:
In cor.test.default(epi$Prov2rutinTPK, epi$Prov2rutinAPTT, use =
"complete.obs",  :
  Cannot compute exact p-value with ties
> #RESULT:
> plot(epi$Prov2rutinTPK, epi$Prov2rutinAPTT, xlab="", ylab = "", cex.axis=3,
cex=2.5, pch=19, axes=F)
> axis(side=1,lwd=4,cex.axis=3, labels=NA)
> axis(side=1,lwd=0,cex.axis=3, line=1)
> axis(side=2,lwd=4,cex.axis=3, labels=NA)
> axis(side=2,lwd=0,cex.axis=3, line=0)
>
>
> #Prov2rutinTPK vs PIVKA
> cor.test(epi$Prov2rutinTPK, epi$PIVKAMpostop, use="complete.obs",
method="pearson")

	Pearson's product-moment correlation
```

```
data:  epi$Prov2rutinTPK and epi$PIVKAMpostop
t = -0.55884, df = 27, p-value = 0.5809
alternative hypothesis: true correlation is not equal to 0
95 percent confidence interval:
 -0.4555833  0.2701612
sample estimates:
       cor
-0.1069325

> cor.test(epi$Prov2rutinTPK, epi$PIVKAMpostop, use="complete.obs",
method="spearman")

    Spearman's rank correlation rho

data:  epi$Prov2rutinTPK and epi$PIVKAMpostop
S = 4468.1, p-value = 0.6039
alternative hypothesis: true rho is not equal to 0
sample estimates:
      rho
-0.100505

Warning message:
In cor.test.default(epi$Prov2rutinTPK, epi$PIVKAMpostop, use =
"complete.obs",  :
  Cannot compute exact p-value with ties
> #RESULT:
> plot(epi$Prov2rutinTPK, epi$PIVKAMpostop, xlab="", ylab = "", cex.axis=3,
cex=2.5, pch=19, axes=F,ylim=c(0,130))
>
>
>
> #Prov2rutinTPK vs CRP
> cor.test(epi$Prov2rutinTPK, epi$Prov2rutinCRP, use="complete.obs",
method="pearson")

    Pearson's product-moment correlation

data:  epi$Prov2rutinTPK and epi$Prov2rutinCRP
t = -1.4718, df = 28, p-value = 0.1522
alternative hypothesis: true correlation is not equal to 0
95 percent confidence interval:
 -0.5729300  0.1021591
sample estimates:
       cor
-0.2679728

> cor.test(epi$Prov2rutinTPK, epi$Prov2rutinCRP, use="complete.obs",
method="spearman")

    Spearman's rank correlation rho

data:  epi$Prov2rutinTPK and epi$Prov2rutinCRP
S = 4873.1, p-value = 0.6585
alternative hypothesis: true rho is not equal to 0
sample estimates:
```

```
        rho
-0.08412151

Warning message:
In cor.test.default(epi$Prov2rutinTPK, epi$Prov2rutinCRP, use =
"complete.obs",  :
  Cannot compute exact p-value with ties
> #RESULT:
> plot(epi$Prov2rutinTPK, epi$Prov2rutinCRP, xlab="", ylab = "", cex.axis=3,
cex=2.5, pch=19, axes=F)
> axis(side=1,lwd=4,cex.axis=3, labels=NA)
> axis(side=1,lwd=0,cex.axis=3, line=1)
> axis(side=2,lwd=4,cex.axis=3, labels=NA)
> axis(side=2,lwd=0,cex.axis=3, line=0)
>
>
> #Prov2extemMCF vs PIVKA
> cor.test(epi$Prov2extemMCF, epi$PIVKAMpostop, use="complete.obs",
method="pearson")

    Pearson's product-moment correlation

data:  epi$Prov2extemMCF and epi$PIVKAMpostop
t = -0.89872, df = 29, p-value = 0.3762
alternative hypothesis: true correlation is not equal to 0
95 percent confidence interval:
 -0.4903501  0.2014808
sample estimates:
       cor
-0.1646114

> cor.test(epi$Prov2extemMCF, epi$PIVKAMpostop, use="complete.obs",
method="spearman")

    Spearman's rank correlation rho

data:  epi$Prov2extemMCF and epi$PIVKAMpostop
S = 6448.9, p-value = 0.1009
alternative hypothesis: true rho is not equal to 0
sample estimates:
       rho
-0.3001744

Warning message:
In cor.test.default(epi$Prov2extemMCF, epi$PIVKAMpostop, use =
"complete.obs",  :
  Cannot compute exact p-value with ties
> #RESULT:
> plot(epi$Prov2extemMCF, epi$PIVKAMpostop, xlab="", ylab = "", cex.axis=3,
cex=2.5, pch=19, axes=F)
> axis(side=1,lwd=4,cex.axis=3, labels=NA)
> axis(side=1,lwd=0,cex.axis=3, line=1)
> axis(side=2,lwd=4,cex.axis=3, labels=NA)
> axis(side=2,lwd=0,cex.axis=3, line=0)
>
> #Correlation between fibrinogen and Fibtem
```

```
> dev.new(width=5)
> # plot(epi$FIBpreop, epi$Prov2fibtemMCF, xlab="P-fibrinogen (g/L)", ylab =
"ROTEM FIBTEM MCF (mm)", abline(lm(epi$Prov2fibtemMCF~ epi
$FIBpreop)),cex.main=1.3, ylim=c(10,52),cex.lab=1.4, main="FIBTEM-MCF vs
Fibrinogen")
> plot(epi$FIBpreop, epi$Prov2fibtemMCF, xlab="P-fibrinogen (g/L)", ylab =
"ROTEM FIBTEM MCF (mm)",cex.main=1.3, ylim=c(10,52),cex.lab=1.4, main="FIBTEM-
MCF vs Fibrinogen")
> cor.test(epi$FIBpreop, epi$Prov2fibtemMCF, use="complete.obs",
method="pearson")

    Pearson's product-moment correlation

data:  epi$FIBpreop and epi$Prov2fibtemMCF
t = 1.3124, df = 30, p-value = 0.1993
alternative hypothesis: true correlation is not equal to 0
95 percent confidence interval:
 -0.1259042  0.5379999
sample estimates:
      cor
0.2330198

> cor.test(epi$FIBpreop, epi$Prov2fibtemMCF, use="complete.obs",
method="spearman")

    Spearman's rank correlation rho

data:  epi$FIBpreop and epi$Prov2fibtemMCF
S = 4097, p-value = 0.1692
alternative hypothesis: true rho is not equal to 0
sample estimates:
      rho
0.2490805

Warning message:
In cor.test.default(epi$FIBpreop, epi$Prov2fibtemMCF, use = "complete.obs",  :
  Cannot compute exact p-value with ties
> #result: NS
> # text(x=6, y=41, "N/S", pos=3, cex=2)
> arrows(1.85,9,,25,code=0,lwd=2,lty=3)
> arrows(2,9,4,,code=0,lwd=2,lty=3)
>
>
> plot(epi$FIBpostop, epi$Prov2fibtemMCF, xlab="", ylab = "", cex.axis=3,
cex=2.5, pch=19, axes=F)
> axis(side=1,lwd=4,cex.axis=3, labels=NA)
> axis(side=1,lwd=0,cex.axis=3, line=1)
> axis(side=2,lwd=4,cex.axis=3)
> #axis(side=2,lwd=4,cex.axis=3)
>
>
> #Correlation between fibrinogen and withdrawal time
>
> plot(epi$withdrawaltime, epi$FIBpreop, xlab="Day of epidural withdrawal",
ylab = "P-fibrinogen (g/L)",abline(lm(epi$FIBpreop~epi
$withdrawaltime)),cex.main=1.3, cex.lab=1.4)
```

```
> cor.test(epi$withdrawaltime, epi$FIBpreop, use="complete.obs",
method="pearson")

    Pearson's product-moment correlation

data:  epi$withdrawaltime and epi$FIBpreop
t = 0.50375, df = 30, p-value = 0.6181
alternative hypothesis: true correlation is not equal to 0
95 percent confidence interval:
 -0.2655909  0.4266535
sample estimates:
       cor
0.09158469

> cor.test(epi$withdrawaltime, epi$FIBpreop, use="complete.obs",
method="spearman")

    Spearman's rank correlation rho

data:  epi$withdrawaltime and epi$FIBpreop
S = 4996.4, p-value = 0.6467
alternative hypothesis: true rho is not equal to 0
sample estimates:
      rho
0.0842434

Warning message:
In cor.test.default(epi$withdrawaltime, epi$FIBpreop, use = "complete.obs",  :
  Cannot compute exact p-value with ties
> text(x=14, y=4.5, "N/S", pos=3, cex=2)
> #text(x=13, y=640, "r=0.56", pos=3, cex=2)
>
> #Correlation between aPTT and INTEM-MCF
>
> plot(epi$Prov2rutinAPTT, epi$Prov2intemMCF, xlab="aPTT (s)", ylab = "ROTEM
INTEM MCF (mm)", abline(lm(epi$Prov2intemMCF~epi$Prov2rutinAPTT)),cex.main=1.3,
cex.lab=1.4)
> cor.test(epi$Prov2rutinAPTT, epi$Prov2intemMCF, use="complete.obs",
method="pearson")

    Pearson's product-moment correlation

data:  epi$Prov2rutinAPTT and epi$Prov2intemMCF
t = -0.34078, df = 31, p-value = 0.7356
alternative hypothesis: true correlation is not equal to 0
95 percent confidence interval:
 -0.3960925  0.2882640
sample estimates:
        cor
-0.06109077

> cor.test(epi$Prov2rutinAPTT, epi$Prov2intemMCF, use="complete.obs",
method="spearman")

    Spearman's rank correlation rho
```

```
data:  epi$Prov2rutinAPTT and epi$Prov2intemMCF
S = 5773.4, p-value = 0.8459
alternative hypothesis: true rho is not equal to 0
sample estimates:
       rho
0.03518698

Warning message:
In cor.test.default(epi$Prov2rutinAPTT, epi$Prov2intemMCF, use =
"complete.obs",  :
  Cannot compute exact p-value with ties
> text(x=40, y=62, "N/S", pos=3, cex=2)
> #text(x=13, y=640, "r=0.56", pos=3, cex=2)
>
> #Correlation between aPTT and INTEM-CT
>
> plot(epi$Prov2rutinAPTT, epi$Prov2intemCT, xlab="aPTT (s)", ylab = "ROTEM
INTEM CT (s)", abline(lm(epi$Prov2intemCT~epi$Prov2rutinAPTT)),cex.main=1.3,
cex.lab=1.4)
> cor.test(epi$Prov2rutinAPTT, epi$Prov2intemCT, use="complete.obs",
method="pearson")

	Pearson's product-moment correlation

data:  epi$Prov2rutinAPTT and epi$Prov2intemCT
t = -0.89483, df = 31, p-value = 0.3778
alternative hypothesis: true correlation is not equal to 0
95 percent confidence interval:
 -0.4760559  0.1952656
sample estimates:
       cor
-0.1586808

> cor.test(epi$Prov2rutinAPTT, epi$Prov2intemCT, use="complete.obs",
method="spearman")

	Spearman's rank correlation rho

data:  epi$Prov2rutinAPTT and epi$Prov2intemCT
S = 6583.8, p-value = 0.5789
alternative hypothesis: true rho is not equal to 0
sample estimates:
      rho
-0.100237

Warning message:
In cor.test.default(epi$Prov2rutinAPTT, epi$Prov2intemCT, use =
"complete.obs",  :
  Cannot compute exact p-value with ties
> text(x=39, y=170, "N/S", pos=3, cex=2)
> #text(x=13, y=640, "r=0.56", pos=3, cex=2)
>
> #Correlation between PT-INR and EXTEM-CT
> plot(epi$Prov2rutinPK, epi$Prov2extemCT, xlab="PT-INR", ylab = "ROTEM EXTEM
CT (s)", abline(lm(epi$Prov2extemCT~epi$Prov2rutinPK)),cex.main=1.3,
cex.lab=1.4)
```

```
> cor.test(epi$Prov2rutinPK, epi$Prov2extemCT, use="complete.obs",
method="pearson")

    Pearson's product-moment correlation

data:  epi$Prov2rutinPK and epi$Prov2extemCT
t = 1.1177, df = 31, p-value = 0.2723
alternative hypothesis: true correlation is not equal to 0
95 percent confidence interval:
 -0.1571117  0.5059370
sample estimates:
      cor
0.1968129

> cor.test(epi$Prov2rutinPK, epi$Prov2extemCT, use="complete.obs",
method="spearman")

    Spearman's rank correlation rho

data:  epi$Prov2rutinPK and epi$Prov2extemCT
S = 5462.4, p-value = 0.6296
alternative hypothesis: true rho is not equal to 0
sample estimates:
       rho
0.08716727

Warning message:
In cor.test.default(epi$Prov2rutinPK, epi$Prov2extemCT, use =
"complete.obs",  :
  Cannot compute exact p-value with ties
> text(x=1.5, y=45, "N/S", pos=3, cex=2)
> #text(x=13, y=640, "r=0.56", pos=3, cex=2)
>
>
>
> #Correlation between PT-INR and EXTEM-MCF
> plot(epi$Prov2rutinPK, epi$Prov2extemMCF, xlab="PT-INR", ylab = "ROTEM EXTEM
MCF (mm)", abline(lm(epi$Prov2extemMCF~epi$Prov2rutinPK)),cex.main=1.3,
cex.lab=1.4)
> cor.test(epi$Prov2rutinPK, epi$Prov2extemMCF, use="complete.obs",
method="pearson")

    Pearson's product-moment correlation

data:  epi$Prov2rutinPK and epi$Prov2extemMCF
t = -1.4426, df = 31, p-value = 0.1592
alternative hypothesis: true correlation is not equal to 0
95 percent confidence interval:
 -0.5470235  0.1012061
sample estimates:
       cor
-0.2508175

> cor.test(epi$Prov2rutinPK, epi$Prov2extemMCF, use="complete.obs",
method="spearman")
```

```
	Spearman's rank correlation rho

data:  epi$Prov2rutinPK and epi$Prov2extemMCF
S = 6546.7, p-value = 0.6027
alternative hypothesis: true rho is not equal to 0
sample estimates:
        rho 
-0.09402775 

Warning message:
In cor.test.default(epi$Prov2rutinPK, epi$Prov2extemMCF, use = 
"complete.obs",  :
  Cannot compute exact p-value with ties
> text(x=1.5, y=65, "N/S", pos=3, cex=2)
> #text(x=13, y=640, "r=0.56", pos=3, cex=2)
> 
> 
> 
> 
> #Correlation between FII and PIVKA
> #Preop
> plot(epi$F2preop, epi$PIVKAMpreop, xlab="Preoperative FII (kIE/L)", ylab = 
"Preoperative mean PIVKA (µg/L)", abline(lm(epi$PIVKAMpreop~epi
$F2preop)),cex.main=1.3, cex.lab=1.4)
> cor.test(epi$F2preop, epi$PIVKAMpreop, use="complete.obs", method="pearson")

	Pearson's product-moment correlation

data:  epi$F2preop and epi$PIVKAMpreop
t = 0.81739, df = 36, p-value = 0.4191
alternative hypothesis: true correlation is not equal to 0
95 percent confidence interval:
 -0.1930289  0.4358591
sample estimates:
     cor 
0.134984 

> cor.test(epi$F2preop, epi$PIVKAMpreop, use="complete.obs", method="spearman")

	Spearman's rank correlation rho

data:  epi$F2preop and epi$PIVKAMpreop
S = 7512.8, p-value = 0.2852
alternative hypothesis: true rho is not equal to 0
sample estimates:
     rho 
0.177936 

Warning message:
In cor.test.default(epi$F2preop, epi$PIVKAMpreop, use = "complete.obs",  :
  Cannot compute exact p-value with ties
> text(x=1, y=10, "Spearman: Rho=0.17//P=0.029", pos=3, cex=2)
> text(x=1, y=20, "Pearson: t=0.81//P=0.042", pos=3, cex=2)
> 
> #Postop
> plot(epi$postop, epi$PIVKAMpostop, xlab="Postoperative FII (kIE/L)", ylab = 
```

```
"Postoperative mean PIVKA (µg/L)", abline(lm(epi$PIVKAMpostop~epi
$F2postop)),cex.main=1.3, cex.lab=1.4)
Error in xy.coords(x, y, xlabel, ylabel, log) :
  'x' and 'y' lengths differ
> cor.test(epi$F2postop, epi$PIVKAMpostop, use="complete.obs",
method="pearson")

	Pearson's product-moment correlation

data:  epi$F2postop and epi$PIVKAMpostop
t = 0.62829, df = 29, p-value = 0.5347
alternative hypothesis: true correlation is not equal to 0
95 percent confidence interval:
 -0.2486658  0.4516782
sample estimates:
      cor
0.1158852

> cor.test(epi$F2postop, epi$PIVKAMpostop, use="complete.obs",
method="spearman")

	Spearman's rank correlation rho

data:  epi$F2postop and epi$PIVKAMpostop
S = 3200, p-value = 0.05082
alternative hypothesis: true rho is not equal to 0
sample estimates:
      rho
0.3548387

> text(x=1.2, y=300, "Spearman: Rho=0.35//P=0.051", pos=3, cex=2)
> text(x=1, y=200, "Pearson: t=0.62//P=0.53", pos=3, cex=2)
>
> #####
> #Correlation between postop extem MCF och F13.
> plot(epi$Prov2extemMCF, epi$F13postop, xlab="postop extem MCF", ylab =
"postop F13", abline(lm(epi$F13postop~epi$Prov2extemMCF)),cex.main=1.3,
cex.lab=1.4)
> cor.test(epi$Prov2extemMCF, epi$F13postop, use="complete.obs",
method="pearson")

	Pearson's product-moment correlation

data:  epi$Prov2extemMCF and epi$F13postop
t = 2.6074, df = 29, p-value = 0.01426
alternative hypothesis: true correlation is not equal to 0
95 percent confidence interval:
 0.09631896 0.68443758
sample estimates:
      cor
0.4357859

> cor.test(epi$Prov2extemMCF, epi$F13postop, use="complete.obs",
method="spearman")

	Spearman's rank correlation rho
```

```
data:  epi$Prov2extemMCF and epi$F13postop
S = 3359.7, p-value = 0.07668
alternative hypothesis: true rho is not equal to 0
sample estimates:
      rho
0.3226419

Warning message:
In cor.test.default(epi$Prov2extemMCF, epi$F13postop, use = "complete.obs",  :
  Cannot compute exact p-value with ties
>
> #Correlation between postop intem MCF och F13.
> plot(epi$Prov2intemMCF, epi$F13postop, xlab="postop intem MCF", ylab =
"postop F13", abline(lm(epi$F13postop~epi$Prov2intemMCF)),cex.main=1.3,
cex.lab=1.4)
> cor.test(epi$Prov2intemMCF, epi$F13postop, use="complete.obs",
method="pearson")

    Pearson's product-moment correlation

data:  epi$Prov2intemMCF and epi$F13postop
t = 1.7327, df = 29, p-value = 0.09377
alternative hypothesis: true correlation is not equal to 0
95 percent confidence interval:
 -0.05389246  0.59595588
sample estimates:
      cor
0.3062968

> cor.test(epi$Prov2intemMCF, epi$F13postop, use="complete.obs",
method="spearman")

    Spearman's rank correlation rho

data:  epi$Prov2intemMCF and epi$F13postop
S = 3332.6, p-value = 0.07155
alternative hypothesis: true rho is not equal to 0
sample estimates:
      rho
0.3281047

Warning message:
In cor.test.default(epi$Prov2intemMCF, epi$F13postop, use = "complete.obs",  :
  Cannot compute exact p-value with ties
>
> #Correlation between postop fibtem MCF och F13.
> plot(epi$Prov2fibtemMCF, epi$F13postop, xlab="postop fibtem MCF", ylab =
"postop F13", abline(lm(epi$F13postop~epi$Prov2fibtemMCF)),cex.main=1.3,
cex.lab=1.4)
> cor.test(epi$Prov2fibtemMCF, epi$F13postop, use="complete.obs",
method="pearson")

    Pearson's product-moment correlation

data:  epi$Prov2fibtemMCF and epi$F13postop
```

```
t = 2.8328, df = 29, p-value = 0.008309
alternative hypothesis: true correlation is not equal to 0
95 percent confidence interval:
 0.1331763 0.7037890
sample estimates:
      cor
0.4655473

> cor.test(epi$Prov2fibtemMCF, epi$F13postop, use="complete.obs",
method="spearman")

    Spearman's rank correlation rho

data:  epi$Prov2fibtemMCF and epi$F13postop
S = 2650.5, p-value = 0.008296
alternative hypothesis: true rho is not equal to 0
sample estimates:
      rho
0.4656307

Warning message:
In cor.test.default(epi$Prov2fibtemMCF, epi$F13postop, use = "complete.obs",  :
  Cannot compute exact p-value with ties
>
```
