# Supplementary material for: Coagulative safety of epidural catheters after major upper gastrointestinal surgery: advanced and routine coagulation analysis in 38 patients
Source: Perioper Med (Lond). 2016 Oct 18;5:28. doi: 10.1186/s13741-016-0053-0 (PMC5067910; doi:10.1186/s13741-016-0053-0)
Supplement: Additional file 4: — Case of patient with thrombocytopenia and low factor XIII. Table showing pre- and postoperative laboratory results for this patient. (PDF 170 kb) [file 13741_2016_53_MOESM4_ESM.pdf]

Supplementary File 4: Test results from the patient with thrombocytopenia and low factor XIII at inadvertent withdrawal of their epidural catheter.

	Preoperative	Postoperative	Reference Range
Hb (g/L)	129	133	117-170
PT-INR	1.1	1.5	0.9-1.2
aPTT (s)	27	32	26-33
Plc ($x10^6$/L)	175	35	145–387
Multiplate® ADP AUC	60	29	57-113
ROTEM® EXTEM MCF (mm)	58	54	50-72
Creatinine (µmol/L)	77	77	45-105
Bilirubin (µmol/L)	8	8	5-25
ALP (µkat/L)	0.75	0.91	0.6-1.8
Fibrinogen (g/L)	2.05	4.50	2.0-4.0
FVII (kIE/L)	0.84	0.77	0.6-1.6
FXII (kIE/L)	0.92	0.72	1.07-1.5
FXIII (kIE/L)	1.06	0.38	0.7-1.4
PIVKA (mg/L)	**2.03**	2.62	<2.0

Results are underlined to show values outside the reference range.

His preoperative questionnaire and preoperative test results were unremarkable other that the patient reported tablet treated hypertension and a subjective tendency to develop bruises after minor trauma, which had begun around two years ago. The duration of his gastrectomy was 9 hours; 300ml haemorrhage was recorded during the operation and 1130 ml fluid was collected from his drainage during the first 24 hours postoperatively. His Hb decreased from 145 to 122g/L during this period but his first postoperative Plc was $47x10^6$/L. The led to suspicion of infection and he was given a prolonged course of antibiotics with cefuroxime but he did not subsequently develop a clinical infection. His epidural catheter was inadvertently withdrawn on the third postoperative day: shortly afterwards, Plc was measured as $35x10^6$/L; ROTEM results were within the normal range but he had a low FXIII of 0.38 kIE/L. He did not develop any neuraxial complications. At follow up two years after the operation he had returned to subjectively normal health. Underlined values indicate results outside the reference range.
